# Consecutive Marcus Electron and Proton Transfer in Heme Peroxidase Compound II-Catalysed Oxidation Revealed by Arrhenius Plots

**DOI:** 10.1038/s41598-019-50466-9

**Published:** 2019-10-01

**Authors:** Audrius Laurynėnas, Marius Butkevičius, Marius Dagys, Sergey Shleev, Juozas Kulys

**Affiliations:** 1 Life Sciences Center, Vilnius University, Saulėtekio al. 7, LT-10257 Vilnius, Lithuania; 2 Malmö University, Jan Waldenströmsgata 25, SE-214 28 Malmö, Sweden

**Keywords:** Biocatalysis, Biocatalysis, Biophysical chemistry, Enzyme mechanisms, Metalloproteins

## Abstract

Electron and proton transfer reactions in enzymes are enigmatic and have attracted a great deal of theoretical, experimental, and practical attention. The oxidoreductases provide model systems for testing theoretical predictions, applying experimental techniques to gain insight into catalytic mechanisms, and creating industrially important bio(electro)conversion processes. Most previous and ongoing research on enzymatic electron transfer has exploited a theoretically and practically sound but limited approach that uses a series of structurally similar (“homologous”) substrates, measures reaction rate constants and Gibbs free energies of reactions, and analyses trends predicted by electron transfer theory. This approach, proposed half a century ago, is based on a hitherto *unproved* hypothesis that pre-exponential factors of rate constants are similar for homologous substrates. Here, we propose a novel approach to investigating electron and proton transfer catalysed by oxidoreductases. We demonstrate the validity of this new approach for elucidating the kinetics of oxidation of “non-homologous” substrates catalysed by compound II of *Coprinopsis cinerea* and *Armoracia rusticana* peroxidases. This study – using the Marcus theory – demonstrates that reactions are not only limited by electron transfer, but a proton is transferred *after* the electron transfer event and thus both events control the reaction rate of peroxidase-catalysed oxidation of substrates.

## Introduction

Oxidation and reduction reactions are the most common biochemical transformations in biology. They are catalysed by oxidoreductases, a diverse class of enzymes evolved to facilitate electron transfer (ET). Therefore, an improved qualitative and quantitative understanding of ET catalysed by these enzymes is pivotal to advance practical applications, such as the selection of mediators for bioconversion systems or the rational engineering of enzymes for bioelectrochemical sensors, biofuel cells, and other technological processes. For example, peroxidases have applications in feedstock delignification^1^, dye decolorization^2^, xenobiotics degradation^3^, and biosensing^4^. As a result, ET is a common subject of scientific research, especially for enzymes of current practical importance, such as heme peroxidase (HP).

HP is a diverse class of oxidoreductases found in fungi, plants, and animals. These enzymes catalyse oxidation reactions via the following reaction scheme^5^:

1 $$\begin{array}{lll}({\rm{I}}){E}_{{\rm{red}}}+{{\rm{H}}}_{2}{{\rm{O}}}_{2} & \mathop{\to }\limits^{{k}_{{{\rm{H}}}_{2}{{\rm{O}}}_{2}}} & {\rm{CpdI}}+{{\rm{H}}}_{2}{\rm{O}}\\ ({\rm{II}}){\rm{CpdI}}+S+{H}^{+} & \mathop{\to }\limits^{{k}_{1}} & {\rm{CpdII}}+{S}^{\cdot +}\\ (\mathrm{III})\,\mathrm{CpdII}+S+{H}^{+} & \mathop{\to }\limits^{{k}_{2}} & {E}_{{\rm{red}}}+{S}^{\cdot +}+{{\rm{H}}}_{2}{\rm{O}}\end{array}$$

Here, the reduced form of enzyme *E*_red_ is oxidized by hydrogen peroxide. The resultant intermediate form compound I (CpdI) is reduced back to *E*_red_ via CpdII with two reduction steps (each consisting of one electron and one proton transfer). Possibly the most remarkable feature of HP is its wide range of possible substrates, S, that can be oxidised. These include phenols, indoleacetic acids, phenylendiamines, phenoxazines,and phenothiazines^6–10^. Notably, the most comprehensive data are available for the rate constant *k*_2_and a range of substrates, because it represents a much slower step than *k*_1_ ^6–10^.

On one hand, this lack of specificity allows for a wide range of applications of HP enzymes, e.g. horseradish peroxidase (HRP) or with the lesser known, but more active peroxidase from *Coprinopsis cinerea* (CIP). On the other hand, it may appear puzzling how these enzymes are able to oxidise such a variety of substrates. There have been various attempts resolve this apparent paradox^6–10^, all using Marcus theory, created in the late 1960s and laid out in seminal papers relating the bimolecular ET reaction rate constant with properties of reactants via the famous equation^11–18^:

2 $${k}_{{\rm{ET}}}={{\rm{K}}}_{{\rm{eq}}}\frac{\sqrt{\pi }}{\hslash \sqrt{\lambda {k}_{{\rm{B}}}T}}{V}_{{\rm{if}}}^{2}{{\rm{e}}}^{-\frac{{(\Delta {G}^{0}+\lambda )}^{2}}{4\lambda {k}_{{\rm{B}}}T}}$$

where *V*_if_ is the electronic coupling between the initial and final state electronic wave functions, T the temperature, *k*_B_ the Boltzmann constant, *ħ* the reduced Planck constant, *λ* the solvent and inner reorganisation energy of a reaction, Δ*G*^0^ Gibbs energy of the reaction, and *K*_eq_ the equilibrium constant of transitional complex formation from reactants.

Previous studies attempted to explain the observed oxidation rate constants,*k*_1_ and *k*_2_ from Eq. 1, based on the physicochemical properties of substrates^6–10^, measuring rate constants using different series of *homologous* compounds. This led to the axiom that homologous compounds should have similar reaction reorganisation energies and pre-exponential factors in Eq. 2. Therefore, the only variable that changes for different sets of homologous compounds is Δ*G*^0^ and there should exist a parabolic relation between ln(*k*_ET_) and Δ*G*^0^. However, this relation can only exist if ET limits the reaction rate. This type of reasoning has also been applied to other oxidoreductases^19–27^. In fact, semi-parabolic dependencies between both *k*_1_ and *k*_2_ and Δ*G*^0^ were found for both CpdI and CpdII of HRP and CIP and a variety of substrates, which led to the conclusion that both *k*_1_ and *k*_2_ are ET^6–10^. This conclusion appears surprising because the substrate oxidation steps II and III in Eq. 1 involve both ET and proton transfer (PT). Due to the greater weight of a proton, the PT is generally much slower than an ET, suggesting that *k*_1_ and *k*_2_ are PT rather than ET limited, which calls the previous research on HP catalysis into question.

We believe that this contradiction arises from the use of homologous series of compounds, which limits the range of Δ*G*^0^ and makes it impossible to investigate the entire range of catalytic properties of HPs and other oxidoreductases. Therefore, we suggest that the investigation of *non-homologous* series of compounds can yield an explanation that is in better agreement with the points raised above. To test this, we selected CpdII from HRP and CIP and a set of substrates that had been used in previous publications, which differ in reduction potential, structure, and the number of electrons and protons donated during the oxidation process (12 substrates, 13 oxidation reactions in total). To provide a picture of the CpdII reduction mechanism that is as complete as possible, we also measured the temperature dependencies of *k*_2_ for each reaction, calculated the quantum chemical self-exchange solvent and inner reorganisation energies (*λ*_s_), estimated the change of *λ*_s_ for compounds in enzyme substrate complexes, and measured the kinetic isotope effects for the oxidation rate of the chosen compounds.

## Results and Discussion

### Kinetics and thermodynamics of substrates catalysed by CpdII

We measured the rate constants (*k*_2_) and activation free energies (Δ*G*^‡^) with pre-exponential factors (*A*) for all 12 substrates at pH 7.00 (experimental details, structures, and names of substrates are available in the Supporting Information (SI)). At 25 °C, the rate constants ranged from about 2 × 10^6^ to 5 × 10^8^ M^−1^ s^−1^ for CIP and from about 1 × 10^5^ to 3 × 10^8^ M^−1^ s^−1^ for HRP (concerns about possible diffusion-limited kinetics are addressed in the SI). These values are comparable with those published previously^9,10^. Measured Δ*G*^‡^ values varied from 0 ± 1 to 11.6 ± 2 kcal mol^−1^ for CIP and from 4.7 ± 0.9 to 9.7 ± 0.8 kcal mol^−1^ for HRP. To the best of our knowledge, there are no published values to which we could compare these results.

**Figure 1:**
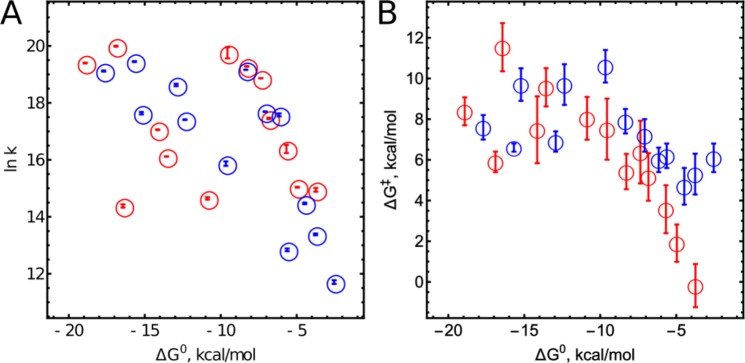
(**A**) Dependence of logarithms of apparent bimolecular CpdII reduction rate constants on Δ*G*° at 25 °C; (**B**) Dependence of Δ*G*^‡^ on Δ*G*°; Red circles indicate results related to CIP, blue circle indicate those related to HRP.

The reaction free energies were calculated from $\Delta {G}^{0}=-F({E}_{{\rm{CpdII}}}^{1}-{E}_{s}^{1})$, using the relevant one-electron reduction potentials (RP) of CpdII (${E}_{{\rm{CpdII}}}^{1}$ = 0.982 V for CIP^28^and 0.93 V for HRP^29^ relevance of these reduction potentials to the ET is discussed in the SI (Page S68). One can expect the reduction potentials of enzymes and substrates to be different in the enzyme-substrate complex. However, since the reduction potentials were measured in previous work using redox titration with different substrates, we assume that these effects have already been included in the published redox potentials of both CpdIIs. The measurements and calculations of ${E}_{s}^{1}$ RP and *λ*_*s*_ for substrates are described in the SI. The results for CIP are summarised in Table 1 (HRP results are presented in the SI, Table S3) and illustrated in Fig. 1. As expected for non-homologous substrates, *k*_2_ depends on Δ*G*^0^.

**Table 1:** Kinetic and thermodynamic parameters of CIP-catalysed substrate oxidation.

Substrate	*k*_2_, M^−1^ s^−1^, at 25 *C*^○^	${{\boldsymbol{E}}}_{{\boldsymbol{s}}}^{{\bf{1}}}$, V vs. NHE	*λ*_*s*_, kcal/mol^a^	ln *A*	Δ*G*^‡^, kcal/mol	*θ*_s_^b^
ABTS	3.8 ± 0.1 × 10^7^	0.686 ± 0.006	3.42	26.8 ± 2.	5.5 ± 1.1	0.43
AMB	1.003 ± 0.003 × 10^7^	0.394 ± 0.005	11.17	31.5 ± 1.9	9.1 ± 1.1	0.36
CPZ	3.1 ± 0.2 × 10^6^	0.79 ± 0.01	12.04	14.6 ± 1.7	0 ± 1.	0.41
DCPIP(I)	3.8 ± 0.8 × 10^8^	0.568^c^	12.47	32.2 ± 2.1	7.4 ± 1.2	0.36
DCPIP(II)	1.3 ± 0.2 × 10^7^	0.736 ± 0.01	3.77	21.9 ± 0.6	3.3 ± 0.3	0.39
DMB	2.3 ± 0.1 × 10^6^	0.511 ± 0.004	11.04	28.7 ± 2.0	8.0 ± 1.2	0.37
HEPX	1.56 ± 0.01 × 10^8^	0.663 ± 0.003	3.10	29.3 ± 2.0	6.2 ± 1.2	0.35
MB	2.66 ± 0.03 × 10^8^	0.162^c^	9.12	33.5 ± 0.7	8.4 ± 0.4	0.41
PPSA	2.37 ± 0.03 × 10^8^	0.623 ± 0.003	2.65	28.3 ± 1.3	5.3 ± 0.8	0.37
PZ	3.40 ± 0.05 × 10^6^	0.767 ± 0.006	11.82	18.7 ± 1.3	2.2 ± 0.8	0.39
TH	4.80 ± 0.07 × 10^8^	0.250^c^	8.85	30.0 ± 0.9	5.9 ± 0.8	0.36
TMPD	1.76 ± 0.01 × 10^7^	0.270 ± 0.006	11.68	36.3 ± 3.4	11.6 ± 2.	0.36
VB	2.56 ± 0.03 × 10^7^	0.369^c^	8.15	29.3 ± 2.7	7.2 ± 1.6	0.38

^a^Theoretically calculated self-exchange (inner and solvent) reorganization energies; ^b^ratio of solvent-accessible surface in a docked enzyme-substrate complex; ^c^theoretical values(details in SI).

The reduction potentials, ${E}_{s}^{1}$, of the substrates vary from 0.162 to 0.790 V – a broad range that is unattainable by using any kind of series of homologous compounds. The self-exchange (solvent and inner) reorganisation energies, *λ*_*s*_, were estimated quantum chemically by using optimised structures from reduction potential calculations (*cf*., Table 1 with more details in the SI)^30–36^. The calculated values of *λ*_*s*_ range from 3 to 12 kcal mol^−1^ (*i.e*., from 0.13 to 0.52 eV).The *λ*_*s*_ values are comparable for structurally similar compounds; *e.g*., the self-exchange (solvent and inner) reorganisation energies of phenylenediamines and phenoxazines are 11 and 3 kcal mol^−1^, respectively. In contrast to *homologous* series of substrates, our *non-homologous* set allows for probing a broad range of Δ*G*^0^ and *λ*_*s*_.

For comparison, a previous publication, also using the framework of Marcus theory but assuming a limiting ET step, reported the reorganization energy for the oxidation of phenothiazines and phenoxazines as 7 kcal mol^−1^(0.3 eV)^10^. The reorganisation energy of the overall reaction (assuming additivity) can be approximated using^18^:

3 $$\lambda =({\lambda }_{s}{\theta }_{s}+{\lambda }_{{\rm{heme}}})/2$$

where *λ*_*s*_ are the inner and solvent self-exchange reorganisation energies calculated in water and listed in Table 1. In order to account for reorganisation energy in an enzyme-substrate complex, an additional factor, *θ*_*s*_, is used (see Table 1 for CIP values;values for HRP are in Table S3). *θ*_*s*_ is calculated as the ratio of solvent-accessible surface (SAS) of the substrate in docked enzyme-substrate complexes to the SAS of free substrate (docking methodology, structures, and calculation details are provided in the SI). This relation can be justified from the large difference between static and optical dielectric constants in water and protein^37,38^. Possible uncertainties in *θ*_*s*_ are due to uncertainties in the docking procedures.

Using existing in the literature values of *λ*_*s*_, *θ*_*s*_ and ${\lambda }_{{\rm{heme}}}$ (0.5 eV) for cytochrome *c* and HRP, we obtained similar values (~0.3 eV) from Eq. 3 to those published for the overall reaction reorganisation energies for phenothiazines and pheoxazines^39,40^. Therefore, we conclude that the assumed additivity of reorganisation energies (Eq. 3) as well as the scaling of the reorganisation energy with *θ*_*s*_ and the quantum-mechanically calculated values of *λ*_*s*_, accurately summarises observations made by other authors for the overall reorganization energies of oxidation reactions catalysed by CIP. In Eq. 3, there is no dependence of the reorganisation energy on the distance between electron donor and electron acceptor. The analysis of docked complexes revealed that the centres of mass of all susbtrates are at similar distances from the centres of mass ofheme. We therefore conclude that the distance-related correction factor will be similar for all compounds and will reside in theheme reorganisation energy,$\,{\lambda }_{{\rm{heme}}}$, which is calculated from measurements of *k*_2_.

### Calculation of activation free energies Δ*G*^‡^ and *λ*_*s*_ does not support ET-limited CpdII catalysis

**Figure 2:**
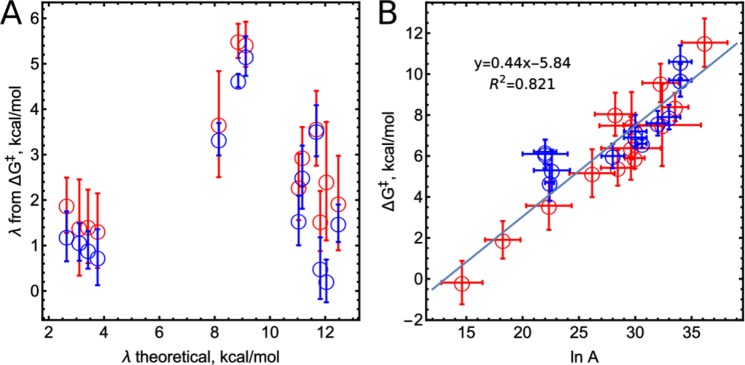
(**A**) Comparison of theoretically calculated *λ* with *λ* calculated from Δ*G*^‡^ and Δ*G*° for all substrates. (**B**) Dependence of Δ*G*^‡^ on the pre-exponential factor ln(*A*). CIP and HRP results are shown as red and blue circles, respectively.

It has been hypothesised that the CpdII-catalysed oxidation of substrates is ET-limited^6–9,41^. We tested this hypothesis by calculating the reorganisation energies, *λ*, from the following form of the Marcus equation:$\,\Delta {G}^{\ddagger }=\frac{{({\Delta G}^{0}+\lambda )}^{2}}{4\lambda }$, which should apply if the ET were the rate-limiting step in the reaction. According to Eq. 3, there should be a correlation between the reorganisation energies calculated from this equation and theoretical computations of *λ*_*s*_. However, we found no such correlation (Fig. 2A), which, in the light of the similarities between the calculated and experimentally found reorganisation energies for phenoxazines and phenothiazines (*vide supra*), appears even more worrying.

Therefore, we hypothesise that the observed value for*k*_2_ is actually a synthetic rate constant that accounts for both the ET and PT rates. This is supported by the linear relation between the measured free energies of activation and the logarithm of pre-exponential factors of the rate constants (Fig. 2B). It seems unlikely that such a relation would exist merely by chance (the probability of such an event for rate parameters of CIP is 1.4 × 10^−5^). A similar correlation could be plotted from the values for formation kinetics of salts of organic amines, although the authors do not claim a linear relation of Δ*G*° and *A*^42^. The existing correlation could be explained by assuming that *k*_2_ is a synthetic rate constant composed of both ET and PT steps.

### *k*_2_ consists of PT and ET rates in CpdII catalysis

**Scheme 1:**
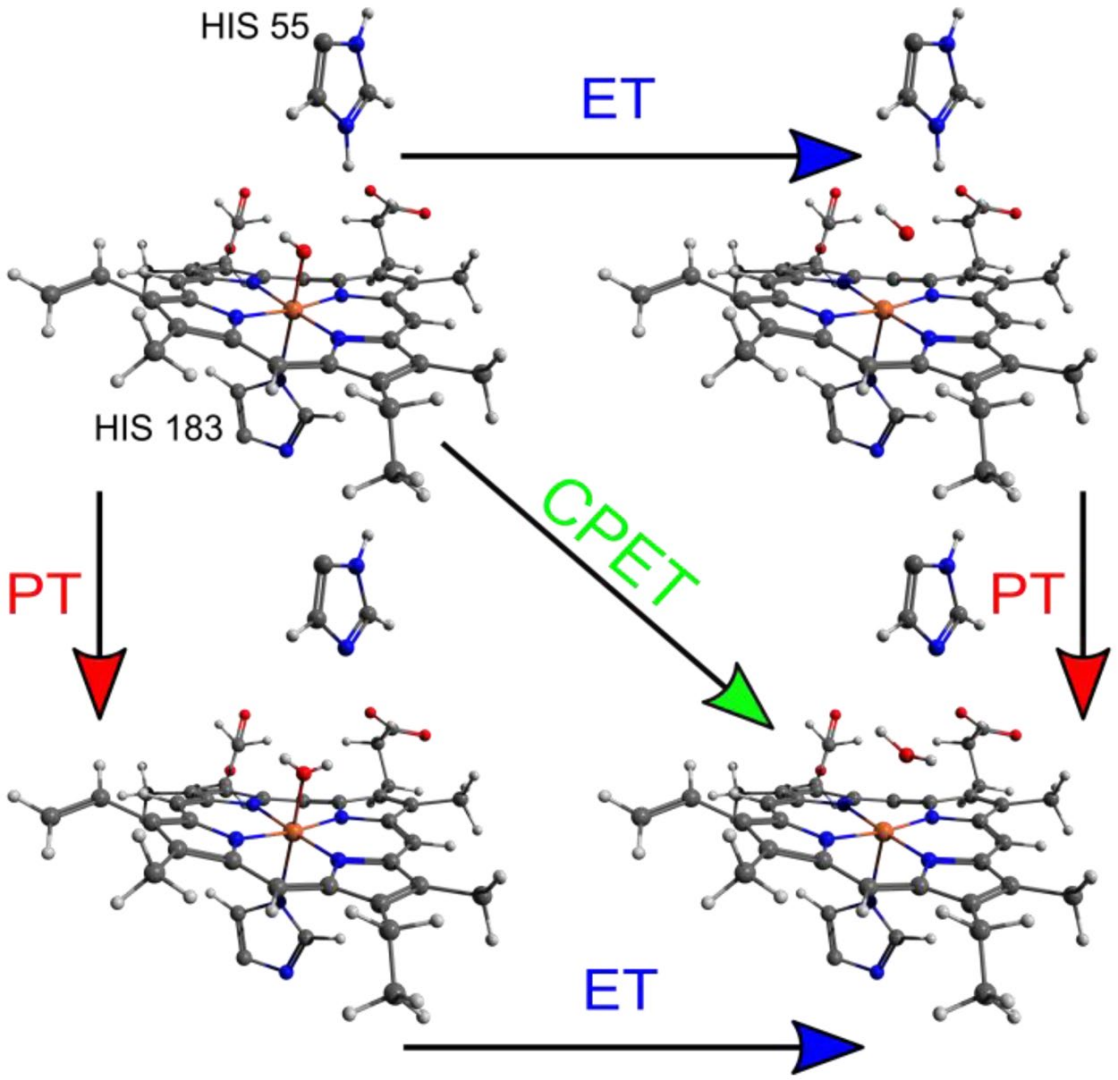
Schematic of possible mechanisms for the CpdII-catalysed oxidation reaction.

There are only few alternatives to the hypothesis that ET is the limiting step in CpdII reduction (Scheme 1). If PT is the limiting step, a proton can be transferred either from the substrate or from a nearby protonated amino acid (*i.e*., internal PT, from HIS 55 for CIP) to the CpdII active centre (heme). However, there can be no PT from the substrate as radical cations are formed in the 9 observed reactions and substrates do not donate protons to the overall reaction (pK_a_ values and additional discussion can be found in the SI, page S67). Instead, water self-ionisation is the proton source for HIS 55. Internal PT (from HIS 55 to heme) can be ruled out as the limiting step because the rate constants for all substrates would have to be the *same*. However, experimental values of *k*_2_ vary by several orders of magnitude for different substrates; Table 1 and Fig. 1).

In the recent literature illustrated pathways in Scheme 1 are collectively termed as proton coupled ET (PCET)^43^. The term, however, was intended to be used only for the reactions where a proton and electron are transferred simultaneously, without intermediate states^44^. Nowadays, it is commonly termed as concerted proton electron transfer (CPET)^43^. The CPET mechanism can be ruled out as a mechanism due to the small kinetic isotope effect (KIE), which has previously been measured for indoleacetic acids as *k*_h_/*k*_d_≈ 1.3 - typical KIEs are much larger in CPET reactions^45–47^. However, the CPET mechanism sometimes exhibits KIE values close to unity^48^,yet theoretical predictions of the dependence of KIE on Δ*G*° indicate that KIE should be maximal for a zero driving force^45^. We observed an entirely different behaviour, where KIE was minimal for a near zero driving force and became maximal for a large driving force (*cf*., the SI section on KIE)^45^. In addition, most substrates lack a labile proton that could accompany ET in the CPET mechanism^48,49^. Therefore, the only remaining mechanism is a consecutive ET-PT. The ET-limited mechanism should be independent on the driving force and exhibits KIE close to 1. The PT mechanism can exhibit nearly any KIE value above 1, depending on many factors, such as barrier height, PT distance, vibronic coupling, etc. Yet, for the internal PT it should be independent on the driving force of the reaction, because previously mentioned factors should be constant. Our finding above and KIE measurements (*cf*. SI section on KIE) dismiss these cases. Therefore, the only alternative is ET-PT mechanism, where oxidation rate of substrates is governed both by ET and by PT rates. This mechanism can be modelled using apparent bimolecular ET and consecutive internal PT schemes that are indistinguishable under quasi-steady state conditions:

5 $$\begin{array}{c}{\rm{CpdII}}+S\mathop{\to }\limits^{{k}_{{\rm{ET}}}}{\rm{CpdIIS}}\\ {\rm{CpdIIS}}\mathop{\to }\limits^{{k}_{{\rm{PT}}}}{E}_{{\rm{red}}}+{S}^{\cdot +}\end{array}\,\,{\rm{and}}\,\,\begin{array}{c}{\rm{CpdII}}\mathop{\to }\limits^{{k}_{{\rm{PT}}}}\mathrm{CpdI}{\rm{I}}^{\prime} \\ \mathrm{CpdI}{\rm{I}}^{\prime} +S\mathop{\to }\limits^{{k}_{{\rm{PT}}}}{E}_{{\rm{red}}}+{S}^{\cdot +}\end{array}$$

The apparent bimolecular rate constant, *k*_2,_ for the total process under these quasi-steady state conditions is dependent on the concentration of substrate (see the SI for a derivation):

6 $${k}_{2}=\frac{{k}_{{\rm{PT}}}{k}_{{\rm{ET}}}}{[S]{k}_{{\rm{ET}}}+{k}_{{\rm{PT}}}}$$

where the rate constant, *k*_ET_, is defined as in Eq. 2 (using the average initial substrate concentrations [*S*] for the analysis;Table S2). The pre-exponential multipliers from Eq. 2, *K*_eq_ and *V*_*if*_, contain the dependence of electronic coupling on the distance between donor and acceptor. The equilibrium constants of the transitional complexes are combined to *apparent electronic coupling* ${V}_{if}^{\text{'}}\,$(therefore, its new dimension is cal mol^−1^ M^−1/2^), which is calculated for every substrate type^11,17,18^. One could argue that these factors should be estimated in order to apply ET theory. However, any uncertainty or error in estimating the equilibrium constants of the formation of transitional complexes, the dependence of electronic coupling on distance, etc., will ultimately reside in the electronic coupling factor, *V*_*if*_, giving a false impression of certainty in the pre-exponential factor, which is actually composed of uncertain multipliers.

The rate constant *k*_PT_ is based on Marcus theory as well. The PT step in Eqs (6) and (7) is a separate internal charge-transfer reaction and depends on pK_a_ of the proton-donor and the proton-acceptor in the CpdII active centre. Therefore, the PT driving force is constant and the free energy of activation of the PT step can be approximated with a constant, *E*_a_, as suggested by Hammes-Schiffer^49^:

7 $${k}_{PT}=\frac{\sqrt{\pi }}{\hslash }{V}^{{}^{{\rm{^{\prime} }}}2}\frac{{{\rm{e}}}^{\frac{-{E}_{a}}{{k}_{B}T}}}{\sqrt{{k}_{B}T}}\,{\rm{w}}{\rm{i}}{\rm{t}}{\rm{h}}\,{V}^{{}^{{\rm{^{\prime} }}}2}={V}_{if,PT}^{2}/\sqrt{{\lambda }_{PT}}$$

**Figure 3:**
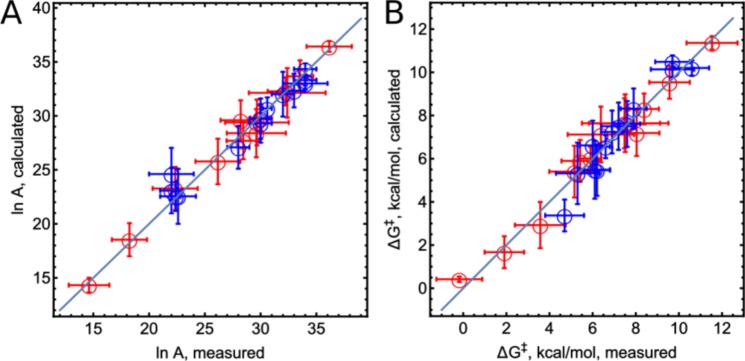
Correlation between calculated and measured values of (**A**) pre-exponential factors and (**B**) free energies of activation Δ*G*^‡^ with confidence intervals, for all investigated CpdII-catalyzed oxidation reactions.The straight line represents a perfect correlation. Red and blue circles indicate results from CIP and HRP, respectively.

The substitution of Eqs (2) and (7) into Eq. (6) results in an explicit expression of *k*_2_. This expression in its linearized form was used to simultaneously fit the pre-exponential factors,*A*, and the free energies of activation, Δ*G*^‡^ (Eqs S10 to S15 in the SI) for all substrates. In the calculations, *λ*_*s*_, *θ*_*s*_, and Δ*G*° are used to calculate the parameters *E*_*a*_, *V*′, *λ*_heme_, and ${V}_{if}^{\text{'}}$ (Table 2). The correlations between the calculated and measured values of *A* and Δ*G*^‡^ (Fig. 3) show that Eq. 6 (involving consecutive ET and PT) is in good agreement with experimental results for both CIP and HRP. Therefore, we conclude that CpdII reduction is a consecutive ET and PT process, where PT also influences the rate constant *k*_2_. This conclusion is further supported by the fitted reorganisation energy of heme (Table 2, bottom line). Values of 0.44 eV (10.2 ± 0.3 kcal/mol) and 0.47 eV (11.0 ± 0.2 kcal/mol) for CIP and HRP, respectively, are both close to the reorganisation energy of ET of heme (0.5 eV) found in cytochrome *c* by Bortolotti *et al*.^39^ and in HRP by Andreu *et al*.^40^. The undefined ${V}_{if}^{^{\prime} }$ value of TMPD (*undefined* means that it was fitted as an unbound value) indicates that the rate of CpdII-catalyzed oxidation of TMPD is exclusively *limited by a PT step*.

**Table 2:** Fitted parameters of the ET/PT model and calculated Δ*G*^‡^_ET_ for the ET step.

Parameter	Value for CIP	Value for HRP	Δ*G*^‡^_*ET*_, kcal ⁄ mol (CIP)	Δ*G*^‡^_*ET*_, kcal ⁄ mol (HRP)
*V* ^ABTS^_*if*_	0.76 ± 0.08 cal M^−1/2^mol^−1^	0.19 ± 0.02 cal M^−1/2^mol^−1^	0.04 ± 0.01	0.02 ± 0.01
*V* ^AMB^_*if*_	3.5 ± 0.4 cal M^−1/2^mol^−1^	4.0 ± 0.5 cal M^−1/2^mol^−1^	1.5 ± 0.1	0.8 ± 0.1
*V* ^CPZ^_*if*_	0.19 ± 0.02 cal M^−1/2^mol^−1^	1.0 ± 0.1 cal M^−1/2^mol^−1^	0.5 ± 0.03	1.0 ± 0.04
*V* ^DCPIP(I)^_*if*_	4.0 ± 0.5 cal M^−1/2^mol^−1^	2.8 ± 0.3 cal M^−1/2^mol^−1^	0.16 ± 0.02	0.01 ± 0.006
*V* ^DCPIP(II)^_*if*_	1.4 ± 0.2 cal M^−1/2^mol^−1^	0.8 ± 0.07 cal M^−1/2^mol^−1^	0.001 ± 0.002	0.12 ± 0.02
*V* ^DMB^_*if*_	2.0 ± 0.2 cal M^−1/2^mol^−1^	4.8 ± 0.6 cal M^−1/2^mol^−1^	0.5 ± 0.05	0.14 ± 0.03
*V* ^HEPX^_*if*_	0.83 ± 0.09 cal M^−1/2^mol^−1^	0.62 ± 0.06 cal M^−1/2^mol^−1^	0.13 ± 0.02	0.001 ± 0.002
V^MB^_if_	260 ± 30 cal M^−1/2^mol^−1^	75 ± 9 cal M^−1/2^mol^−1^	5.1 ± 0.2	3.8 ± 0.2
*V* ^PPSA^_*if*_	1.7 ± 0.1 cal M^−1/2^mol^−1^	1.3 ± 0.1 cal M^−1/2^mol^−1^	0.3 ± 0.04	0.06 ± 0.02
V^PZ^_if_	0.44 ± 0.05 cal M^−1/2^mol^−1^	0.3 ± 0.05 cal M^−1/2^mol^−1^	0.2 ± 0.02	0.57 ± 0.03
V^TH^_if_	76 ± 8 cal M^−1/2^mol^−1^	33 ± 4 cal M^−1/2^mol^−1^	3.9 ± 0.2	2.7 ± 0.2
*V* ^TMPD^_*if*_	undefined	Undefined	3.0 ± 0.2	2.1 ± 0.1
V^VB^_if_	4.7 ± 0.5 cal M^−1/2^mol^−1^	3.4 ± 0.4 cal M^−1/2^mol^−1^	2.1 ± 0.1	1.3 ± 0.1
*V*’	4.3 ± 0.5 cal mol^−1^	1.3 ± 0.07 cal mol^−1^		
E_a_	11.7 ± 0.2 kcal⁄mol	10.7 ± 0.1 kcal⁄mol		
*λ*_heme_	10.2 ± 0.3 kcal⁄mol	11.0 ± 0.2 kcal⁄mol

In contrast to the widely held assumption regarding similarity of *V*_*if*_ for *homologous* compounds (which, to the best of our knowledge, has never been discussed, yet is frequently used in applying Marcus ET theory to series of homologous substrates^6–10,19–25,50^), ${V}_{if}^{^{\prime} }$ (Table 2) varies by up to 5 times between homologous compounds (*e.g*., TH and MB, or CPZ and PZ). Therefore, traditional investigations of the dependence of enzymatic ET rate constants on potentials of homologous substrates only partially reveal the trends predicted by Marcus theory. We measured the rate constants at different temperatures and the results suggested a new way to estimate ${V}_{if}^{^{\prime} }$. This approach is general and does not depend on the assumption of similar ${V}_{if}^{^{\prime} }$ values for a set of homologous substrates.

The proposed model (Eq. 6) splits the consecutive ET-PT process into separate steps and allows estimating of the Marcus free energy of activation of ET $\Delta {G}_{{\rm{ET}}}^{\ddagger }$ for each compound. $\Delta {G}_{\mathrm{ET}\,}^{\ddagger }$ is small and ranges from 0 to 5 kcal/mol. The fitted *E*_*a*_ of the PT step is 11.7 ± 0.3 kcal/mol and the rate constant *k*_PT_ = 520 ± 40 s^−1^ at 298 K for CIP. For HRP, this becomes 10.7 ± 0.1 kcal/mol with a rate constant of *k*_PT_ = 200 ± 30 s^−1^ at 298 K. Thus, the PT step is the main factor controlling Δ*G*^‡^. In this context the Marcus quadratic dependence of activation free energy on the driving force is only a first approximation of a much more complicated ET. Based on the driving forces, four of the investigated compounds appear to fall into the inverted region, where the dependence of activation energy on driving force becomes asymmetric. In such a case, the original Marcus law greatly overestimates the activation energy of the reaction. This asymmetry is more pronounced when the range of driving forces is greater than 2 eV and reactants change their structures appreciably upon ET (which is not applicable here)^51–54^. Then, the vibrational modes start to participate in ET and lower the activation energies. This would affect the compounds VB, TMPD, MB, and TH and could lower their activation free energies of ET by up to several kilocalories per mole (which is within the margins of measurement error). In this context, the experimental free energy of activation for TMPD (11.6 ± 2 kcal/mol) appears high and can only be explained by invoking ET-PT or PT-ET mechanism rather than by pure ET step.

### Physical constraints of PT reveal that ET precedes PT step in CpdII catalysed oxidation

**Figure 4:**
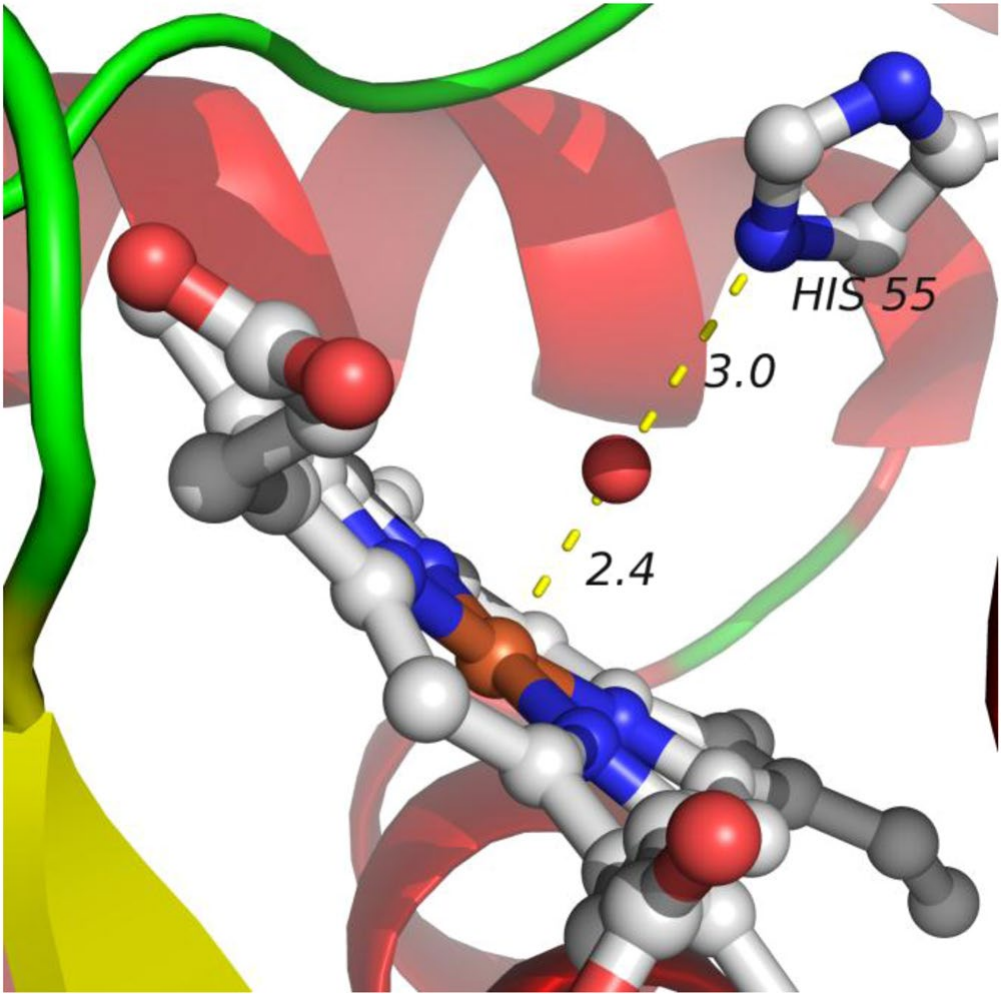
Geometry of the CIP active centre (PDBID:1h3j).

The calculated values of PT rate constants and *E*_*a*_ can be directly related to known structures of peroxidases. It is commonly accepted that in CpdII reduction to ferric peroxidase, the proton is transferred from a protonated HIS 55 (in case of CIP) to a heme-bound oxygen atom^5,55–58^. This step can precede or follow the ET. We assume that the main difference between the ET-PT and PT-ET mechanisms comes from the distances involved in PT. These distances are controlled by the oxidation state of the heme iron and the position of bound oxygen, either in the form of a hydroxyferryl or hydroxide complex. The geometry of ferric (reduced) CIP is known (PDBID:1h3j), and its active centre is illustrated in Fig. 4 ^59^. If ET precedes PT, the positions of heavy atoms before and after the PT step should not change significantly. Therefore, the distance of PT (1.0 ± 0.2 Å) can be estimated by subtracting the bond lengths of N-H and O-H from the distance between the HIS 55 nitrogen and the oxygen atom (3 Å) (Fig. 4). Similarly, by assuming an iron – oxygen distance of about 1.7 Å for CpdII^60^, we estimate the PT distance in the PT-ET mechanism as about 1.7 Å. The PT rate constants are calculated using the multi-state non-adiabatic proton theory by Borgis and Hynes (seethe SI)^61^.

*E*_*a*_of the PT step (11.2 ± 0.3 kcal/mol) and *k*_PT_ (520 ± 40 s^−1^ at 298 K) over a distance of 1.7 Å (PT-ET) can be reproduced with ${\lambda }_{p}=3.72\,{\rm{eV}}$ and $\Delta {G}^{0}=-\,48.3\,{\rm{kcal}}/{\rm{mol}}$. *k*_*PT*_ and *E*_*a*_ over a distance of 0.95 Å (ET-PT) are reproduced with *λ*_*P*_ = 2.01 eV and $\Delta {G}^{0}=-\,3.54\,{\rm{kcal}}/{\rm{mol}}$ whichappear to be more consistent with published values of PT reorganisation energy(around 2 eV)and *pK*_*a*_ ^62–65^. For PT-ET, *pK*_a_ of the proton acceptor in heme should be about 42, which would be too large. Published *pK*_a_ values for CpdII are about 7 for the histidine side-chain and about 9 for heme-bound water^64,65^. The reduction of CpdII should therefore proceed via the ET-PT mechanism for both CIP and HRP (as both have similar catalysis centres and rate parameters). CpdI and CpdII compounds are quite similar in nature, therefore, histidine side-chain *pK*_a_ values of CpdI can partially reflect *pK*_a_ of CpdII. In the literature these values cover a range from 4 to 8^66–68^. However, even taken this information into account, ET-PT mechanism is still much more reasonable compared to PT-ET.

### KIE estimations for CIP lend support to the ET-PT hypothesis

If we assume that Δ*G*^0^, *λ*_p,_ and the deuteron transfer (DT) distance are the same as in the ET-PT mechanism, we obtain a DT rate constant of 295 s^−1^ and a KIE of 1.8 ± 0.2 for the PT step for CIP. The KIE of the full ET-PT process differs for each substrate (Table S2). The lowest KIE value (about 1) is predicted for CPZ, while the largest value (about 1.8) is calculated for phenylenediamines TMPD and AMB. We found the average KIE value to be about 1.4 which appears in good agreement with previously published values for indoleacetic acids^6^. The KIE values for all three CpdII-catalysed oxidation reactions and substrates TMPD, CPZ, and ABTS for CIP were measured to be 1.6 ± 0.2, 1.0 ± 0.1, and 1.3 ± 0.1, respectively (for HRP-CpdII we found a similar behaviour; see the SI). They are in good agreement with calculated values (*cf*. values in the Supporting Table S2) and further support our hypothesis that *ET is followed by the PT* mechanism during the oxidation of substrates catalysed by CpdII.

## Conclusions

We demonstrated a general approach for the investigation of electron and proton transfers in oxidoreductase-catalysed reactions. The measured dependence of the reaction rate constants on temperature (*i.e*., Arrhenius plots) yielded the reaction-free energies of activation as well as the pre-exponential factors needed for the rigorous comparison of theoretical predictions and experimental observations. The approach was applied to the oxidation of non-homologous substrates catalysed by *Coprinopsis cinerea* and *Armoracia rusticana* (horseradish) peroxidases’ compound II. By combining the above approach with quantum chemical calculations and docking studies we were able to compare the experiments with theory which led to new insights about the catalysis mechanism of CpdII. Contrary to commonly held assumptions^8,9,19,22–27^, the apparent electronic coupling constant ${V}_{if}^{\text{'}}$ for different series of homologous substrates *differ considerably*. Moreover, ET is *not* the rate-limiting step for CpdII-catalysed oxidation. It was found that a PT step follows the initial ET step described by Marcus theory. We could explain the value of the PT rate constant based on molecular geometry and solvent reorganisation energies of PT using the harmonic oscillator approximation and non-adiabatic proton transfer theory. The proposed model correctly predicts the observed kinetic isotope effects and further supports that *a PT follows ET* in CpdII catalysis.

## Methods

### Reaction rate constants and free energies of activation

All experiments were carried out in 50 mM phosphate buffer solution, with a pH of 7.00, using a thermostated stopped-flow spectrophotometer (Otsuka RA-401) cell at temperatures ranging from 10 to 30 °C. Kinetic curves were measured for five different initial concentrations for each substrate reaction, and experiments were repeated 5–10 times. Further details on the materials used and the experiments are available in the SI.

### Electrochemical measurements

Electrochemical experiments were performed with a Series G 750 Potentiostat/Galvanostat/ZRA from Gamry Instruments, Inc. (Warminster, PA, USA), controlled by dedicated PHE200 software. All measurements were carried out in a three-electrode glass cell using an Ag|AgCl|3 M KCl reference electrode (210 mV vs. NHE) and a platinum wire counter electrode from BASi (West Lafayette, IN, USA). All electrochemical measurements were performed in 50 mM phosphate buffer solution, with a pH of 7.00, using glassy carbon (3 mm diameter) or gold (1.6 mm diameter) working electrodes, obtained from BASi. Prior to any measurement, the electrodes were polished with diamond paste (particle size of 1 µM, BASi), alumina slurry (particle size of 0.3 µM, BASi) and then sonicated. Gold electrodes were also cleaned electrochemically by running 40 potential sweep cycles from 0 to 1.9 V vs. NHE in 0.5 M H_2_SO_4_ solution. Additional experimental details are presented in the SI.

### Structural and standard reduction potential calculations

All quantum chemical calculations were performed using GAMESS^32^. Specifically, the molecular structures were first optimized by using the MINI basis set and then used as a starting structure for HF/6–31 + G(d,p) energy optimization. After optimization, the Hessians were evaluated and all structures were determined to be at their energetic minima with no imaginary frequencies. Final optimizations were carried out using DFT^33^ with the hybrid B3LYP functional^34^ and the 6–31 + G(d,p) basis set. Energies of solvated species were evaluated at the same level. Restricted open-shell Hartree-Fock wave function (ROHF) was used for radicals; for other structures, the restricted Hartree-Fock wave functions (RHF) were utilized. All calculations were performed using the solvation model density solvent model method^35^ as implemented in GAMESS. Zero point energies were calculated at the HF/6–31 + G(d,p) level. Further details about the calculation are available in the SI.

### Docking studies and calculations of solvent-accessible surface areas

For docking studies, the rCip structure (PDBID:1h3j)^69^ and the HRP (PDBID:1h55)^5^ were used. Crystallized water molecules were removed from the peroxidase structure and the oxygen–iron distance was reduced in order to reflect the existence of the compound II state rather than the reduced state of the peroxidase active centre containing complexed water. The docking of substrates was performed using AutodockVina^70^. Solvent accessible substrate surface areas in the substrate-enzyme complexes were estimated from the docked structures that exhibited the best energy score when using the MSMS method^71^, implemented into USCF Chimera^72^. The docking results are available in the SI.

## Supplementary information

Supplementary Information

## References

[1] Falade Ayodeji O., Nwodo Uchechukwu U., Iweriebor Benson C., Green Ezekiel, Mabinya Leonard V., Okoh Anthony I. (2016). Lignin peroxidase functionalities and prospective applications. MicrobiologyOpen.

[2] Bilal M (2016). Horseradish peroxidase-assisted approach to decolorize and detoxify dye pollutants in a packed bed bioreactor. J. Environ. Manage..

[3] Bilal M, Rasheed T, Iqbal HMN, Yan Y (2018). Peroxidases-assisted removal of environmentally-related hazardous pollutants with reference to the reaction mechanisms of industrial dyes. Sci. Total Environ..

[4] Bollella P (2018). Highly sensitive, stable and selective hydrogen peroxide amperometric biosensors based on peroxidases from different sources wired by Os-polymer: A comparative study. Solid State Ionics..

[5] Berglund GI (2002). The catalytic pathway of horseradish peroxidase at high resolution. Nature..

[6] Candeias LP, Folkes LK, Porssa M, Parrick J, Wardman P (1996). Rates of reaction of indoleacetic acids with horseradish peroxidase compound I and their dependence on the redox potentials. Biochemistry..

[7] Candeias LP, Folkes LK, Wardman P (1997). Factors controlling the substrate specificity of peroxidases: Kinetics and thermodynamics of the reaction of horseradish peroxidase compound I with phenols and indole-3-acetic acids. Biochemistry..

[8] Folkes LK, Candeias LP (1997). Interpretation of the reactivity of peroxidase compounds I and II with phenols by the Marcus equation. FEBS Lett..

[9] Krikstopaitis K (1998). N-Substituted p-Phenylenediamines as Peroxidase and Laccase Substrates. Acta Chem. Scand..

[10] Kulys J, Krikstopaitis K, Ziemys A (2000). Kinetics and thermodynamics of peroxidase- and laccase-catalyzed oxidation of N-substituted phenothiazines and phenoxazines. J. Biol. Inorg. Chem..

[11] Marcus RA (2005). Theoretical relations among rate constants, barriers, and Broensted slopes of chemical reactions. J. Phys. Chem..

[12] Marcus RA (1957). On the theory of oxidation-reduction reactions involving electron transfer. II. Applications to data on the rates of isotopic exchange reactions. J. Chem. Phys..

[13] Marcus RA (1957). On the theory of oxidation-reduction reactions involving electron transfer. III. applications to data on the rates of organic redox reactions. J. Chem. Phys..

[14] Marcus RA (1959). On the Theory of Electrochemical and Chemical Electron Transfer Processes. Can. J. Chem..

[15] Marcus RA (1956). On the theory of oxidation-reduction reactions involving electron transfer. I. J. Chem. Phys..

[16] Marcus RA (1956). Electrostatic free energy and other properties of states having nonequilibrium polarization. I. J. Chem. Phys..

[17] Sutin N (1982). Nuclear, Electronic, and Frequency Factors in Electron-Transfer Reactions. Acc. Chem. Res..

[18] Marcus RA, Sutin N (1985). Electron transfers in chemistry and biology, BBA Rev. Bioenerg..

[19] Xu F (2000). Redox chemistry in laccase-catalyzed oxidation of N-hydroxy compounds. Appl. Environ. Microbiol..

[20] Tetianec L, Kulys J (2009). Kinetics of N-substituted phenothiazines and N-substituted phenoxazines oxidation catalyzed by fungal laccases. Cent. Eur. J. Biol..

[21] Kulys J (1994). Study of the new electron transfer mediators in glucose oxidase catalysis. J. Mol. Catal..

[22] Limitnas N, Limit A, Nivinskas H, Anusevičius I, Šarlauskas J (2001). Quantitative structure-activity relationships in enzymatic single-electron reduction of nitroaromatic explosives: Implications for their cytotoxicity. Biochim. Biophys. Acta - Gen. Subj..

[23] Anusevičius Ž, Šarlauskas J, Čenas N (2002). Two-electron reduction of quinones by rat liver NAD(P)H:quinone oxidoreductase: Quantitative structure-activity relationships. Arch. Biochem. Biophys..

[24] Čènas N, Anusevičius Ž, Nivinskas H, Misevičiene L, Šarlauskas J (2004). Structure-Activity Relationships in Two-Electron Reduction of Quinones. Methods Enzymol..

[25] Anusevičius Ž (1997). Electron transfer reactions of Anabaena PCC 7119 ferredoxin:NADP + reductase with nonphysiological oxidants. Biochim. Biophys. Acta - Bioenerg..

[26] Tsuruoka Nozomu, Sadakane Takuya, Hayashi Rika, Tsujimura Seiya (2017). Bimolecular Rate Constants for FAD-Dependent Glucose Dehydrogenase from Aspergillus terreus and Organic Electron Acceptors. International Journal of Molecular Sciences.

[27] Teresa Bes M, de Lacey AL, Fernandez VM, Gomez-Moreno C (1995). Electron transfer between viologen derivatives and the flavoprotein ferredoxin-NADP + reductase. Bioelectrochemistry Bioenerg..

[28] Farhangrazi ZS (1994). Oxidation-Reduction Properties of Compounds I and II of Arthromyces ramosus Peroxidase. Biochemistry..

[29] Hayashi Y, Yamazaki I (1979). The oxidation-reduction potentials of compound I/compound II and compound II/ferric couples of horseradish peroxidases A2 and C. J. Biol. Chem..

[30] Roy LE, Jakubikova E, Graham Guthrie M, Batista ER (2009). Calculation of one-electron redox potentials revisited. Is it possible to calculate accurate potentials with density functional methods?. J. Phys. Chem. A..

[31] Schmidt MW (1993). General atomic and molecular electronic structure system. J. Comput. Chem..

[32] Gordon Mark S., Schmidt Michael W. (2005). Advances in electronic structure theory. Theory and Applications of Computational Chemistry.

[33] Kohn W., Sham L. J. (1965). Self-Consistent Equations Including Exchange and Correlation Effects. Physical Review.

[34] Becke AD (1993). A new mixing of Hartree-Fock and local density-functional theories. J. Chem. Phys..

[35] Marenich AV, Cramer CJ, Truhlar DG (2009). Universal solvation model based on solute electron density and on a continuum model of the solvent defined by the bulk dielectric constant and atomic surface tensions. J. Phys. Chem. B..

[36] Bader JS, Cortis CM, Berne BJ (1997). Solvation and reorganization energies in polarizable molecular and continuum solvents. J. Chem. Phys..

[37] Krishtalik, L.I., Kuznetsov, A. M. & Mertz, E. L. Electrostatics of proteins: Description in terms of two dielectric constants simultaneously, *Proteins Struct. Funct. Genet*. **28** 174–182, 10.1002/(SICI)1097-0134(199706)28:2<174::AID-PROT6>3.0.CO;2-F (1997). 9188735

[38] Tavernier HL, Fayer MD (2002). Distance Dependence of Electron Transfer in DNA: The Role of the Reorganization Energy and Free Energy. J. Phys. Chem. B..

[39] Bortolotti CA (2011). The reorganization energy in cytochrome c is controlled by the accessibility of the heme to the solvent. J. Phys. Chem. Lett..

[40] Andreu R, Ferapontova EE, Gorton L, Calvente JJ (2007). Direct electron transfer kinetics in horseradish peroxidase electrocatalysis. J. Phys. Chem. B..

[41] Hammel KE, Cullen D (2008). Role of fungal peroxidases in biological ligninolysis. Curr. Opin. Plant Biol..

[42] Eigen, M. & Johnson, J. The kinetics of reactions in solution, Oxford Clarendon Press, http://www.getcited.org/pub/101167442 (1959).

[43] Savéant J-M (2014). Concerted Proton-Electron Transfers: Fundamentals and Recent Developments. Annu. Rev. Anal. Chem..

[44] Huynh MHV, Meyer TJ (2007). Proton-coupled electron transfer. Chem. Rev..

[45] Edwards SJ, Soudackov AV, Hammes-Schiffer S (2009). Analysis of Kinetic Isotope Effects for Proton-Coupled Electron Transfer Reactions^†^. J. Phys. Chem. A..

[46] Soudackov AV, Hammes-Schiffer S (2016). Proton-coupled electron transfer reactions: Analytical rate constants and case study of kinetic isotope effects in lipoxygenase. Faraday Discuss..

[47] Knapp MJ, Rickert K, Klinman JP (2002). Temperature-dependent isotope effects in soybean Lipoxygenase-1: Correlating hydrogen tunneling with protein dynamics. J. Am. Chem. Soc..

[48] Decornez H, Hammes-Schiffer S (2002). Model Proton-Coupled Electron Transfer Reactions in Solution: Predictions of Rates, Mechanisms, and Kinetic Isotope Effects. J. Phys. Chem. A..

[49] Hammes-Schiffer S, Stuchebrukhov AA (2010). Theory of coupled electron and proton transfer reactions. Chem. Rev..

[50] Reifler RG, Smets BF (2000). Enzymatic reduction of 2,4,6-trinitrotoluene and related nitroarenes: Kinetics linked to one-electron redox potentials. Environ. Sci. Technol..

[51] Ulstrup J, Jortner J (1975). The effect of intramolecular quantum modes on free energy relationships for electron transfer reactions. J. Chem. Phys..

[52] Søndergaard NC, Ulstrup J, Jortner J (2006). The effect of anharmonic intramolecular quantum modes on free energy relationships for electron transfer reactions. Chem. Phys..

[53] Van Duyne RP, Fischer SF (1974). A nonadiabatic description of electron transfer reactions involving large free energy changes. Chem. Phys..

[54] Fischer SF, Duyne RPV (1977). On the theory of electron transfer reactions. The naphthalene-·/TCNQ system. Chem. Phys..

[55] Yamazaki, I., Tamura, M. & Nakajima, R. Horseradish peroxidase C, In: *Mol. Cell. Biochem*., Academic Press, pp. 143–153, 10.1007/BF00224608 (1981). 10.1007/BF00224608 7322114

[56] Derat E, Shaik S (2006). Two-state reactivity, electromerism, tautomerism, and “surprise” isomers in the formation of compound II of the enzyme horseradish peroxidase from the principal species, compound I. J. Am. Chem. Soc..

[57] Wong DWS (2009). Structure and action mechanism of ligninolytic enzymes. Appl. Biochem. Biotechnol..

[58] Henriksen A, Smith AT, Gajhede M (1999). The structures of the horseradish peroxidase C-ferulic acid complex and the ternary complex with cyanide suggest how peroxidases oxidize small phenolic substrates. J. Biol. Chem..

[59] Kjalke M (1992). Comparison of structure and activities of peroxidases from Coprinus cinereus, Coprinus macrorhizus and Arthromyces ramosus. Biochim. Biophys. Acta (BBA)/Protein Struct. Mol..

[60] Derat E, Shaik S (2006). An efficient proton-coupled electron-transfer process during oxidation of ferulic acid by horseradish peroxidase: Coming full cycle. J. Am. Chem. Soc..

[61] Borgis D, Hynes JT (1996). Curve crossing formulation for proton transfer reactions in solution. J. Phys. Chem..

[62] Takada T, Kawai K, Fujitsuka M, Majima T (2005). Contributions of the distance-dependent reorganization energy and proton-transfer to the hole-transfer process in DNA. Chem. - A Eur. J..

[63] Smedarchina Z, Enchev V (1994). Comparative theoretical study of intramolecular proton transfer in the photochemical cycles of 2-(2′-hydroxyphenyl)benzoxazole and 5,8-dimethyl-1-tetralone. J. Photochem. Photobiol. A Chem..

[64] Hashimoto S, Tatsuno Y, Kitagawa T (1986). Resonance Raman evidence for oxygen exchange between the FeIV = O heme and bulk water during enzymic catalysis of horseradish peroxidase and its relation with the heme-linked ionization. Proc. Natl. Acad. Sci..

[65] Abelskov AK, Smith AT, Rasmussen CB, Dunford HB, Welinder KG (1997). pH dependence and structural interpretation of the reactions of Coprinus cinereus peroxidase with hydrogen peroxide, ferulic acid, and 2,2′- azinobis(3-ethylbenzthiazoline-6-sulfonic acid). Biochemistry..

[66] Casadei CM (2014). Neutron cryo-crystallography captures the protonation state of ferryl heme in a peroxidase. Science (80-.)..

[67] Chreifi G (2016). Crystal structure of the pristine peroxidase ferryl center and its relevance to proton-coupled electron transfer. Proc. Natl. Acad. Sci..

[68] Chreifi G (2015). Enzymatic mechanism of leishmania major peroxidase and the critical role of specific ionic interactions. Biochemistry..

[69] Houborg K (2003). Impact of the physical and chemical environment on the molecular structure of Coprinus cinereus peroxidase. Acta Crystallogr. - Sect. D Biol. Crystallogr..

[70] Gime X, Bofill JM, Gonza J (2007). Algorithm to Evaluate Rate Constants for Polyatomic Chemical Reactions. II. Applications. J. Comput. Chem..

[71] Sanner, M. F., Olson, A. J. & Spehner, J. Reduced surface: An efficient way to compute molecular surfaces, *Biopolymers*. **38** 305–320, 10.1002/(sici)1097-0282(199603)38:3<305::aid-bip4>3.3.co;2-8 (1996). 10.1002/(SICI)1097-0282(199603)38:3%3C305::AID-BIP4%3E3.0.CO;2-Y 8906967

[72] Pettersen EF (2004). UCSF Chimera - A visualization system for exploratory research and analysis. J. Comput. Chem..

