# Supplementary material for: Consecutive Marcus Electron and Proton Transfer in Heme Peroxidase Compound II-Catalysed Oxidation Revealed by Arrhenius Plots
Source: Sci Rep. 2019 Oct 1;9:14092. doi: 10.1038/s41598-019-50466-9 (PMC6773748; doi:10.1038/s41598-019-50466-9)
Supplement: Supplementary file 1 — Supplementary Information [file 41598_2019_50466_MOESM1_ESM.docx]

Supporting Information

Consecutive Marcus Electron and Proton Transfer in Heme Peroxidase Compound II-Catalysed Oxidation Revealed by Arrhenius plots

Audrius Laurynenas^*^,^†^ Marius Butkevicius,^†^ Marius Dagys,^†^ Sergey Shleev, ^‡^ and Juozas Kulys^†^

^†^ Life Sciences Center, Vilnius University, Saulėtekio al. 7, LT-10257, Vilnius, Lithuania

^‡^ Malmö University, Jan Waldenströmsgata 25, SE-214 28, Malmö, Sweden

^*^corresponding author

Contents

[1. Materials and experimental methods S2](#_Toc14778755)

[1.1. Materials S2](#_Toc14778756)

[1.2. Spectrophotometric methods S3](#_Toc14778757)

[1.3. Electrochemical methods S5](#_Toc14778758)

[1.4. Preparations of buffer solutions with viscogens, measurements of viscosity, and dependence of reduced TH rate constant on viscosity S5](#_Toc14778759)

[1.5. Preparations of deuterium oxide solutions used in measurements of kinetic isotope effect S6](#_Toc14778760)

[2. Theoretical models S6](#_Toc14778761)

[2.1. Calculations of compounds’ structure and reduction potentials. S6](#_Toc14778762)

[2.2. Calculations of self-exchange (solvent and inner) reorganization energies of substrates in the solvent. S7](#_Toc14778763)

[3. Linearization of dependence of reaction rate constant on temperature S10](#_Toc14778764)

[4. Calculations of redox potentials S11](#_Toc14778765)

[5. Proton transfer rate constant S13](#_Toc14778766)

[6. Oxidation rate constants and kinetic isotope effect S13](#_Toc14778767)

[7. Kinetics, thermodynamics and electrochemistry S15](#_Toc14778768)

[7.1. Activation energy of compound I formation and kinetic isotope effect S15](#_Toc14778769)

[7.2. Activation energy of HEPX oxidation and its reduction potential S16](#_Toc14778770)

[7.3. Activation energy of ABTS oxidation, its reduction potential and kinetic isotope effect S18](#_Toc14778771)

[7.4. Activation energy of AMB oxidation and its reduction potential S20](#_Toc14778772)

[7.5. Activation energy of PZ oxidation and its reduction potential S21](#_Toc14778773)

[7.6. Activation energy of DMB oxidation and its reduction potential S23](#_Toc14778774)

[7.7. Activation energy of TMPD oxidation, its reduction potential and kinetic isotope effect S25](#_Toc14778775)

[7.8. Activation energy of PPSA oxidation and its reduction potential S26](#_Toc14778776)

[7.9. Activation energy of CPZ oxidation, its reduction potential and kinetic isotope effect S28](#_Toc14778777)

[7.10. Activation energy of MB oxidation and its reduction potential S30](#_Toc14778778)

[7.11. Activation energy of TH oxidation and its reduction potential S31](#_Toc14778779)

[7.12. Activation energy of DCPIP oxidation and its reduction potential S33](#_Toc14778780)

[7.13. Activation energy of VB oxidation and its reduction potential S35](#_Toc14778781)

[9. Docking studies and calculations of solvent accessible surface areas S63](#_Toc14778782)

[10. Derivation of the apparent bimolecular rate constant S66](#_Toc14778783)

[11. Discussion about substrates’ pKa and its relevance to the oxidation *via* radical pathway S67](#_Toc14778784)

[12. Discussion about standard reduction potentials of CIP and HRP S68](#_Toc14778785)

[Supporting references S69](#_Toc14778786)

# 1. Materials and experimental methods

## 1.1. Materials

Recombinant *Coprinopsis cinerea* peroxidase (CIP) was acquired from Novo Nordisk A/S, Denmark. It was additionally purified by anion-exchange chromatography to a Reinheit Zahl (A405/A208) of 2.61. The enzyme was homogeneous as assessed by SDS-PAGE. Horseradish peroxidase (HRP) was purchased from Sigma-Aldrich. It was additionally purified by dialysis against 10 mM potassium phosphate buffer solution, pH 7.0. Promazine hydrochloride (PZ, 98% purity), chlorpromazine hydrochloride (CPZ, 98% purity), sulfuric acid (97%), glycerol, PEG 4000, N,N,N,N-tetramethyl-p-phenylenediaminedihydrochloride (TMPD, 97% purity), deuterium oxide. sucrose, and glucose were purchased from Sigma-Aldrich; potassium dihydrogenphosphate and di-potassium hydrogen phosphate, hydrogen peroxide 30% solution – from Roth; acetonitrile used in chromatography, 2,6-dichlorophenolindophenol sodium salt (DCPIP, 98% purity) were purchased from Merck; 2,2’-azino-bis-(3-ethylbenzthiazoline-6-sulfonic acid) disodium salt (ABTS, 98% purity) was purchased from Fluka; variamine blue (VB), thionine (TH) and methylene blue (MB) were obtained from Reachim Ltd, Russia, were purified further with activated charcoal (Sigma), and recrystallized before use [1]. 1-(N,N-dimethylamine)-4-(4-morpholinyl)benzene (AMB) and 1,4-di(4-morpholinyl) benzene (DMB) were synthesized in University of Copenhagen, Denmark. 10-(2-hydroxyethyl) phenoxazine (HEPX) and 3-(4a,10a-dihydro- phenoxazin-10-yl)-propane-1-sulfonic acid (PPSA) were synthesized in Novo Nordisk A/S, Denmark. Chemical structures of the substrates are presented in Figure S1. PQQ-dependent glucose dehydrogenase was a gift kindly provided by Dr. Rolandas Meškys from Life Sciences Center of Vilnius University, Lithuania.


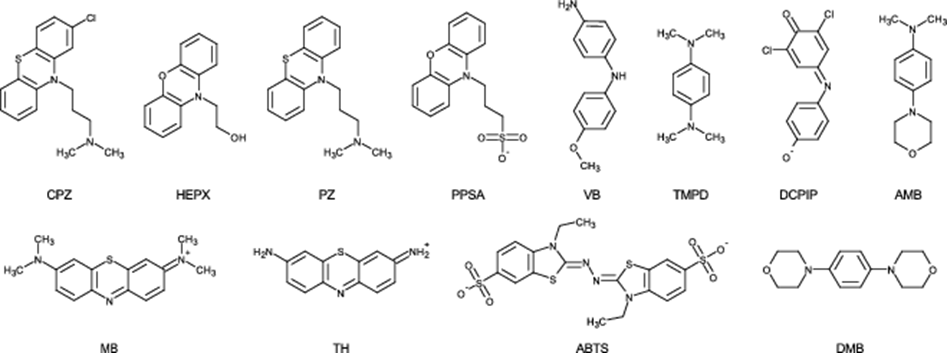


Figure S1. Chemical structures of the substrates used in CIP compound II-catalyzed reduction experiments.

## 1.2. Spectrophotometric methods

**Peroxidase substrate solutions.** Substrates for enzymatic reactions were dissolved in methanol (AMB, DMB, HEPX, VB) or deionized water (DCPIP, ABTS, PPSA, TMPD, CPZ, PZ, TH, MB). Hydrogen peroxide solutions were prepared from a stock solution (30 %) and verified spectrophotometrically at 240 nm using molar absorbance equal to 43.6 M^-1^cm^-1^ [2]. The concentration of CIP was determined spectrophotometrically at 405 nm using molar absorbance 109 mM^-1^cm^-1^ [3]. Molar absorbances for oxidized substrates AMB, DMB, HEPX, DCPIP, ABTS, PPSA, TMPD, CPZ, PZ were found in the literature. In case of VB, TH, MB, molar absorbances were determined after the purification by spectrophotometrical titration. All the molar absorbances used in the study are listed in Table S1. Buffer solutions for peroxidase experiments were prepared by mixing 50 mM K_2_HPO_4_ and 50 mM KH_2_PO_4_ solutions and checking the resultant pH. All the solutions were prepared by using deionized water.

**Peroxidase** concentration in experiments varied from nanomolar to micromolar levels, hydrogen peroxide concentrations in experiments with other substrates were around 110 µM and in all cases they were determined spectrophotometrically after the solutions were prepared. Experiments were carried out with Otsuka RA-401 stopped-flow spectrophotometer from Otsuka Electronics Co., Ltd. (Tokyo, Japan).

Kinetics of CIP and HRP compound I (CpdI) formation from reduced CIP and HRP was measured directly in peroxide reaction without additional reducing substrates. Measurements were carried spectrophotometrically at 405 nm and each experiment was repeated for at least 10 times. An averaged curve of absorbance change over time was fitted directly into the reaction scheme (equation (S22)), using extinction coefficients 109, 109 and 66 M^-1^cm^-1^ for reduced, intermediate and oxidized enzyme forms, respectively. Initial enzyme concentrations were calculated from the first curve point (~0.850 µM), hydrogen peroxide concentration was 4.09 µM. Reaction temperatures varied from 10 to 30 °C.

Experiments of AMB, DMB, HEPX, DCPIP, ABTS, PPSA, TMPD, CPZ, PZ, VB, TH, MB oxidation with CIP and HRP were carried out in 50 mM phosphate buffer solution, pH 7.00, at temperatures in the range from 10 to 30 °C. The data from these experiments were used to estimate the dependence of the reaction rate constant on temperature, and to calculate the free energy of activation. In order to eliminate effects of additional side reactions, concentrations of CIP and substrates were chosen so that a reaction would complete in less than 30 s. This restriction was not applied for HRP kinetics due to much slower reactions. The measurements were repeated up to 20 times, the outliers arising due to faults in initial cuvette filling or other random glitches were discarded, and the average kinetic curve was analyzed. In the cases of AMB, DMB, HEPX, ABTS, PPSA, TMPD, CPZ, PZ, VB, substrate and peroxide solutions were contained in one syringe, and the solution of CIP or HRP in another. The experiments with DCPIP, TH and MB were more complicated due to the oxidized state of these compounds, and the reactions were investigated under anaerobic conditions. The reduced forms of DCPIP, TH and MB were firstly reduced with glucose, catalyzed by PQQ-dependent glucose dehydrogenase (GDH). The GDH concentration was selected so that the presence of enzyme in the reaction mixture would not influence CIP or HRP reaction over short periods of time – typical reduction half-times were 3 to 4 minutes. In Table S1 the wavelengths at which CIP and HRP reaction kinetic curves were measured and the molar absorption coefficients used for data fitting are presented.

Table S1. Molar absorption coefficients and absorbance maxima used for investigation of CIP-catalyzed oxidation of listed compounds. The values were either experimentally determined (marked by asterisk) or taken from the literature (referred accordingly).

| **Compound** | **Absorbance maximum, nm** | **Molar absorbance coefficient, M^-1^cm^-1^** | **Oxidation state of reaction product** |
| --- | --- | --- | --- |
| ABTS [4] | 420 | 3.6×10^4^ | Partial, one-electron |
| AMB [5] | 614 | 9.8×10^3^ | Partial, one-electron |
| MB^*^ | 665 | 6.41×10^4^ | Full, two-electron |
| CPZ [6] | 514 | 8.9×10^3^ | Partial, one-electron |
| DCPIP [7] | 600 | 1.83 × 10^4^ | Full, two-electron |
| DMB [5] | 618 | 9.8×10^3^ | Partial, one-electron |
| HEPX [6] | 524 | 1.6×10^4^ | Partial, one-electron |
| PPSA [6] | 524 | 1.6×10^4^ | Partial, one-electron |
| PZ [6] | 514 | 8.9×10^3^ | Partial, one-electron |
| TMPD [8] | 611 | 1.2×10^4^ | Partial, one-electron |
| TH^*^ | 600 | 6.07×10^4^ | Full, two-electron |
| VB^*^ | 455 | 5.8 ×10^3^ | Full, two-electron |

## 1.3. Electrochemical methods

All the electrochemical experiments were performed using a Series G 750 potentiostat from Gamry Instruments, Inc. (Warminster, PA, USA) in a thermostatted (25 ± 0.1 ^○^*C*) three electrode cell (5 mL), under anaerobic conditions and in a Faraday cage. Reference electrode was Ag|AgCl|3 M KCl (0.210 V vs. NHE) and a platinum wire from BASi (West Lafayette, IN, USA) served as auxiliary electrode. Before each measurement the solutions were purged with argon. 50 mM phosphate buffer solution, pH 7.00, was used as the electrolyte. Glassy carbon (GC, 3 mm diameter) and gold (Au, 1.6 mm diameter) disk electrodes from BASi were used as working electrodes.

Gold electrodes were polished with an alumina paste (0.3 µm diameter from Buehler (Lake Bluff, IL, USA)), sonicated in deionized water for 10 min, and rinsed with water. An additional cleaning step was performed by running 40 electrochemical oxidation-reduction cycles in 0.5 M sulfuric acid between 0 and 1.9 V vs. NHE potentials, at a scan rate of 0.3 Vs^-1^ [9]. Glassy carbon electrodes were polished with the alumina paste, sonicated in acetonitrile for 10 min, then in water for another 10 min, and finally rinsed with water. The electrodes were cleaned prior to all measurements.

All cyclic voltammetry (CV) experiments were performed at various scan rates and potential boundaries. Concentrations of compounds were in the range of tens of micromoles to milimoles, depending on their solubility. ABTS, PZ, CPZ, HEPX, VB, TH, MB, and DCPIP CVs were recorded with glassy carbon, and SYR, AMB, TMPD, DMB, PPSA with gold working electrodes. Measured CVs were used to estimate reduction potentials vs. Ag|AgCl|3 M KCl (0.210 V vs. NHE). In the case of quasi-reversible process with clearly formed oxidation and reduction current peaks, the reduction potential $E_{0}^{'}$was estimated as:

$E_{0}^{'}=\frac{E_{c}+E_{a}}{2}$ (S1)

Where $E_{c}$ is a maximum potential of cathodic peak and $E_{a}$– maximum potential of anodic peak. In the case when oxidation or reduction reactions were irreversible, the reduction potential was calculated from relevant peak [10].

***1.4. Preparations of buffer solutions with viscogens, measurements of viscosity, and dependence of reduced TH rate constant on viscosity***

The buffers with viscogens were prepared by dissolving appropriate amounts of glycerol (from 0% to 50 % w/w), sucrose (from 0% to 50 % w/w) and PEG 4000 (from 0% to 20 % w/w) in minimal amount of deionized water, adding an appropriate amount of stock 200 mM potassium phosphate buffer solution at pH 7.00 and diluting the resulted solution by respective viscogens to target concentration of 50 mM of potassium phosphate at pH 7.00. The viscosities of these solutions were measured using the calibrated capillary straight tube viscometer. The rate constants of CpdI formation and oxidation of reduced TH were measured in each of buffer solutions. The kinetic and viscosity measurements were carried out at 25 °C.

***1.5. Preparations of deuterium oxide solutions used in measurements of kinetic isotope effect***

Kinetic isotope effects (KIE) were measured for CpdI formation and oxidation of TMPD, CPZ, and ABTS for CIP, as well as TMPD, CPZ, ABTS, and HEPX for HRP. The measurements were carried identically to the measurements of the free energy of activation at 25 °C. For these measurements the deuterium oxide (D_2_O) buffer solutions were prepared from the stock solution of 200 mM phosphate buffer solution at pH 6.59 to get a D_2_O-based buffer solution at pD 7.00 (observed pD is recalculated as pH + 0.41) [11]. The stock buffer solution was dried under vacuum. The residue were dissolved in the minimal amount of D_2_O and left to equilibrate for 1 h, then dried under vacuum again. This procedure was repeated three times and the resulting residues were dissolved in D_2_O to obtain 50 mM phosphate buffer solution at pD 7.00, which was used in measurements. All the solutions of the enzymes and substrates (hydrogen peroxide, TMPD, CPZ, ABTS) were prepared in D_2_O and left to equilibrate for at least 3 h.

# 2. Theoretical models

***2.1. Calculations of compounds’ structure and reduction potentials.***

All quantum chemical calculations were performed by using GAMESS [12,13]. Specifically, the molecular structures were firstly optimized using the MINI basis set and further used as a starting structure for HF/6-31+G(d,p) energy optimization. After optimization the Hessians were evaluated and all the structures were at energetic minima with no imaginary frequencies. Final optimizations were carried out using DFT [14] with hybrid B3LYPfunctional [15] and 6-31+G(d,p) basis set. Energies of solvated species were evaluated at the same level. Restricted open-shell Hartree-Fock wave function (ROHF) was used for radicals; for other structures the restricted Hartree-Fock wave functions (RHF) were utilized. All calculations were performed using the solvation model density (SMD) solvent model method [16], as implemented in GAMESS. Zero point energies were calculated at HF/6-31+G(d,p) level. Calculations were performed using simplified thermodynamic cycle in the form of isodesmic reaction [17]:

$A_{\mathrm{solv}}^{\mathrm{ox}}+Ref_{\mathrm{solv}}^{\mathrm{red}}\overset{\Delta G_{vs.ref}^{0}}{\to}A_{\mathrm{solv}}^{\mathrm{red}}+Ref_{\mathrm{solv}}^{\mathrm{ox}}$ (S2)

The Gibbs free energy of the process, described by Equation S2, is expressed as:

$\Delta G_{vs.ref}^{0}=E(A_{\mathrm{solv}}^{\mathrm{red}})-E(A_{\mathrm{solv}}^{\mathrm{ox}})+E(Ref_{\mathrm{solv}}^{\mathrm{ox}})-E(Ref_{\mathrm{solv}}^{\mathrm{red}})$ (S3)

Here *E* refers to energies with added zero point energy. As for reference, the energy of ABTS was selected. These calculations are referred to 1 atm standard state, and transition to 1 M standard state is guided by:

$\Delta G_{vs.ref}^{0}(1 M)=\Delta G_{vs.ref}^{0}(1 \mathrm{atm})+RT\ln(24.46)$ (S4)

The absolute standard reduction potential is calculated as:

$E_{vs.ref}^{0}=\frac{-\Delta G_{vs.ref}^{0}}{F}$ (S5)

This value is converted to standard reduction potential vs. NHE using the value of 4.43 V [18]:

$E^{0}(vs.NHE)=E_{vs.ref}^{0}-E_{\mathrm{ref}}^{0}-4.43$ (S6)

For the cases of two-electron reduction, standard reduction potential is calculated as an average of standard potentials of two separate one-electron reduction steps.

***2.2. Calculations of self-exchange (solvent and inner) reorganization energies of substrates in the solvent.***

Self-exchange (solvent and inner) reorganization energies of the substrates were calculated using quantum mechanical reorganization energy estimation method with solvation model [17]:

$\begin{matrix} \lambda=(\lambda_{1}+\lambda_{2})\text{/}2, \\ \mathrm{where}\lambda_{1}=E(A \mathrm{at} A^{+)}-E\left( A \mathrm{at} A \right)\mathrm{and} \lambda_{2}=E(A^{+}\mathrm{at} A)-E(A^{+}\mathrm{at} A^{+)} \end{matrix}$ (S7)

Here *λ*_1_ represents solvent and inner reorganization energy of oxidation process and is calculated as energy of reduced molecule at geometry of oxidized molecule; *λ*_2_ represents a reorganization energy of the opposite process.

***2.3. Calculations of rate constants in diffusion-controlled reactions.***

The rate constant of diffusion-controlled reaction can be defined as Smoluchowski limit [19]:

$k_{\mathrm{diff}}=1000 \alpha4\pi(R_{s}+R_{e})(D_{s}+D_{e})N_{A}$ (S8)

Here the dimension of $k_{\mathrm{diff}}$ is expressed in M^-1^ s^-1^. *R*_s_ and *R*_e_ are the radiuses of substrate and enzyme in the spherical approximation, respectively, *D*_s_ and *D*_e_ are the diffusion coefficients of substrate and enzyme, respectively; *N*_A_ is the Avogadro number, and *α* is the ratio between active and total enzyme surface. The diffusion coefficients were calculated from Stokes-Einstein equation [20]:

$D=\frac{k_{B}T}{6\pi R\mu(T)}$ (S9)

Here $\mu(T)$ is a function which describes dependence of viscosity on temperature. Here $\mu\left( T \right)=\frac{2.414\times{10}^{-5}\times{10}^{247.8}}{T-140}\left( \mathrm{kg}m^{-1}s^{-1} \right)$ [21] describes the viscosity of water at different temperatures. Assuming that the radius of enzyme is ~30 Å (calculated from PDBID: 1h3j) [22], the radius of substrates (calculated using molecular structures) is ~5 Å and *α* is from 0.01 to 0.1, the resultant rate constants are from 10^8^ to 10^9^ M^-1^ s^-1^. The free energy of activation for all the cases is 4.6 kcal/mol.

The measured apparent bimolecular rate constants (*k*_cat_/*K*_M_) were from ~2×10^6^ to ~5×10^8^ M^-1^ s^-1^. The oxidation of reduced forms of HEPX, DCPIP, PPSA, MB and TH are fast processes and the rate values are relatively close to ones in diffusion-limited reactions. Calculated values of free energies of activation for some of investigated substrates are larger than 10^8^ M^-1^ s^-1^ and thus are above predicted limit for diffusion-controlled reaction (i.e. >4.6 kcal/mol). However, for the sake of completeness, we measured the dependence of rate constant on reaction medium viscosity for TH, the compound of which the oxidation rate constant was the largest. In this case, if the rate constant would be a diffusion-limited, the linear relationship between the relative viscosity of medium and the relative reaction rate constant should stand true. This relation comes directly from Einstein-Stokes equation (Eq. S9) and Smoluchowski (Eq. S8) limit:

$k_{diff, obs}=\frac{2000 \alpha R T {(R_{E}+R_{S})}^{2}}{3 \mu_{\mathrm{obs}} R_{E}R_{S}}$ (S10)

By denoting the buffer solution viscosity as *µ*_0_ and the rate constant in that buffer solution as $k_{diff, 0}$, the ratio of rate constants on different viscosities is equal to the ratio of viscosities itself:

$\frac{k_{diff, 0}}{k_{diff, obs}}=\frac{\mu_{\mathrm{obs}}}{\mu_{0}}=\mu_{\mathrm{relative}}$ (S11)

There are only a few of commonly used viscogens to increase and control the viscosity of reaction medium, namely glycerol, sucrose and PEG. In case of glycerol, it was found that it completely inhibits the formation of CpdI at glycerol concentrations higher than 20% w/w. In case of sucrose, at concentrations from 0 to 50% w/w, the small but noticeable effects on CpdI formation are observed, as sucrose inhibits the CpdI reaction with TH (data not shown). PEG 4000, on the other hand, at concentrations from 0 to 20% w/w, does not noticeably affect the rate constant of CpdI formation, but affects the limiting rate constant of reduced TH oxidation (Figure S2; also *cf.* Section 6.11).


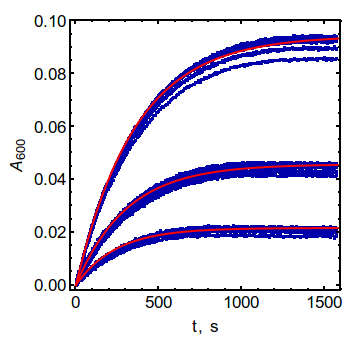

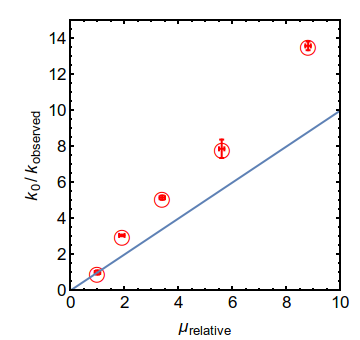


Figure S2. An example of CIP-catalyzed TH oxidation kinetic measurement data at following conditions: PEG content - 10 % w/w, 50 mM phosphate buffer solution, pH 7.00, [CIP] – 21 nM, [H_2_O_2_] – 100 µM, [TH] – from 0.4 to 1.8 µM (*left*); the measurement at each TH concentration was repeated 10 times. The dependence of the ratio of rate constants on viscosity of reaction solution, at PEG 4000 concentrations of 0, 5, 10, 15 and 20 % w/w (*right*); the line represents the expected relation if reaction would be diffusion-controlled.

The established dependence of rate constants on viscosity cannot be attributed to diffusion-controlled reaction, because the data points are well above the theoretical line, whereas a diffusion-controlled reaction should have the slope of such dependence in between 0 and 1. In general, there could be one of the three cases:

1. The reaction rate is limited by solution viscosity (i.e. reaction rate is diffusion-limited). The model describing such case is:

$k_{\mathrm{obs}}=\frac{k_{\mathrm{reaction}}\frac{k_{\mathrm{diffusion}}}{\mu_{\mathrm{relative}}}}{k_{\mathrm{reaction}}+\frac{k_{\mathrm{diffusion}}}{\mu_{\mathrm{relative}}}}$ (S12)

Here $k_{\mathrm{reaction}}$ is the rate constant of enzymatic reaction and $k_{\mathrm{diffusion}}$ is the rate constant of diffusion-limited reaction at relative buffer solution viscosity value of 1.

1. PEG 4000 inhibits peroxidase activity (in reduction of CpdI to CpdII)

The inhibition of peroxidase by PEG 4000 in the limit of small substrate concentration results in the equation presented below:

$k_{\mathrm{obs}}=\frac{k_{\mathrm{reaction}}K_{\mathrm{inhibition}}}{[I]+K_{\mathrm{inhibition}}}$ (S13)

Here $K_{\mathrm{inhibition}}$ is an inhibition constant and [*I*] is a concentration of inhibitor.

1. Reaction rate is affected both by inhibition and by viscosity. The observed rate constant in such case is then affected both by viscosity and inhibition:

$k_{\mathrm{obs}}=\frac{k_{\mathrm{reaction}}K_{\mathrm{inhibition}}k_{\mathrm{diffusion}}}{([I]+K_{\mathrm{inhibition}})(\mu_{\mathrm{relative}}k_{\mathrm{association}}+k_{\mathrm{diffusion}})}$ (S14)

Here $k_{\mathrm{association}}$ is a rate constant of enzyme-substrate complex formation.

In our case, however, data do not fit in a linear dependence expected from diffusion-limited reaction (case 1) nor does it fit in combined viscosity - inhibition effect model (case 3) and could be explained only by PEG 4000 inhibition (case 1). Therefore, we conclude that reaction of the reduced TH oxidation is not limited by diffusion, despite a very large apparent bimolecular rate constant. This conclusion is supported by the fact that the observed free energy of activation for TH oxidation is larger than predicted for diffusion-limited kinetics.


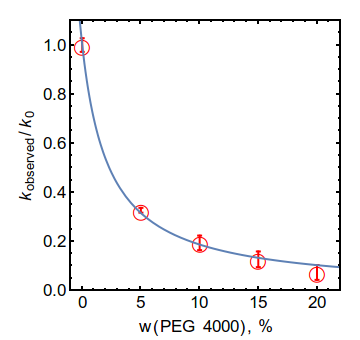

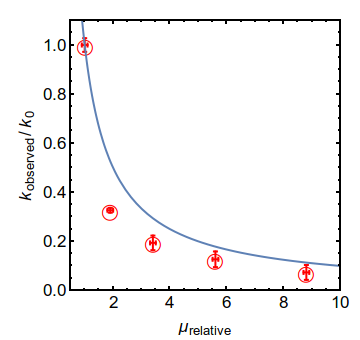


Figure S3. Values of rate constants fitted to the PEG 4000-inhibition model (case 2) (*left*); values of rate constants fitted to the diffusion-limited model (case 1) (*right*).

# 3. Linearization of dependence of reaction rate constant on temperature

The free energy of activation is defined as:

$\Delta G^{\ddagger}=-k_{B}\frac{\delta\ln(k(T))}{\delta(1\text{/}T)}$ (S15)

It is a second term of corresponding Taylor series:

$\ln k(T)=\ln k(T_{0})+\left. \frac{\delta\ln k(T)}{\delta(\frac{1}{T})} \right|_{T=T_{0}}(\frac{1}{T}-\frac{1}{T_{0}})+...$ (S16)

Apparent pre-exponential factor is then calculated as:

$\left. \ln A \right|_{T=T_{0}}=\ln k(T_{0})-\left. \frac{\delta\ln k(T)}{\delta(\frac{1}{T})} \right|_{T=T_{0}}\frac{1}{T_{0}}$ (S17)

and apparent free energy of activation as:

$\left. \Delta G^{\ddagger} \right|_{T=T_{0}}=-k_{B}\left. \frac{\delta\ln k(T)}{\delta(\frac{1}{T})} \right|_{T=T_{0}}$ (S18)

With consideration of Equation 9, the expressions for ln*k*(*T*_0_) and $\left. \frac{\delta\ln k(T)}{\delta(\frac{1}{T})} \right|_{T=T_{0}}$ are calculated as:

$$\ln k(T_{0})=ln(\frac{\sqrt{2\pi}V_{\text{if},a}^{2}V_{p,a}^{2}}{\hbar(\sqrt{2}S\sqrt{T_{0}k_{B}}V_{\text{if},a}^{2}e^{\frac{\text{Ea}_{p}}{T_{0}k_{B}}}+V_{p,a}^{2}\sqrt{T_{0}k_{B}(\lambda_{\text{heme}}+\theta_{s}\lambda_{s})}\exp(\frac{{(2{\Delta\text{G}}_{0}+\lambda_{\text{heme}}+\theta_{s}\lambda_{s})}^{2}}{8T_{0}k_{B}(\lambda_{\text{heme}}+\theta_{s}\lambda_{s})}))})$$

(S19)

And

$$\left. \frac{\delta\ln k(T)}{\delta(\frac{1}{T})} \right|_{T=T_{0}}=(-4T_{0}k_{B}\left( \lambda_{\text{heme}}+\theta_{s}\lambda_{s} \right)\left( \sqrt{2}S\sqrt{T_{0}k_{B}}V_{\text{if},a}^{2}e^{\frac{\text{Ea}_{p}}{T_{0}k_{B}}}+V_{p,a}^{2}\sqrt{T_{0}k_{B}\left( \lambda_{\text{heme}}+\theta_{s}\lambda_{s} \right)}\exp\left( \frac{\left( 2{\Delta\text{G}}_{0}+\lambda_{\text{heme}}+\theta_{s}\lambda_{s} \right)^{2}}{8T_{0}k_{B}\left( \lambda_{\text{heme}}+\theta_{s}\lambda_{s} \right)} \right) \right)$$

$$+8\sqrt{2}S\text{Ea}_{p}\sqrt{T_{0}k_{B}}V_{\text{if},a}^{2}(\lambda_{\text{heme}}+\theta_{s}\lambda_{s})e^{\frac{\text{Ea}_{p}}{T_{0}k_{B}}}+V_{p,a}^{2}\left( 2{\Delta\text{G}}_{0}+\lambda_{\text{heme}}+\theta_{s}\lambda_{s} \right)^{2}\sqrt{T_{0}k_{B}\left( \lambda_{\text{heme}}+\theta_{s}\lambda_{s} \right)}\exp(\frac{\left( 2{\Delta\text{G}}_{0}+\lambda_{\text{heme}}+\theta_{s}\lambda_{s} \right)^{2}}{8T_{0}k_{B}\left( \lambda_{\text{heme}}+\theta_{s}\lambda_{s} \right)}))/$$

$$(8k_{B}(\lambda_{\text{heme}}+\theta_{s}\lambda_{s})(\sqrt{2}S\sqrt{T_{0}k_{B}}V_{\text{if},a}^{2}e^{\frac{\text{Ea}_{p}}{T_{0}k_{B}}}+V_{p,a}^{2}\sqrt{T_{0}k_{B}(\lambda_{\text{heme}}+\theta_{s}\lambda_{s})}\exp(\frac{{(2{\Delta\text{G}}_{0}+\lambda_{\text{heme}}+\theta_{s}\lambda_{s})}^{2}}{8T_{0}k_{B}(\lambda_{\text{heme}}+\theta_{s}\lambda_{s})})))$$

(S20)

**4. Calculations of redox potentials**

A wide range of available substrates have been used in our studies and a well-studied reaction scheme is given as follows [23]:

$\left( I \right)E_{\mathrm{red}}+H_{2}O_{2}\underset{\to}{k_{H_{2}O_{2}}}CpdI+H_{2}O$

$\left( \mathrm{II} \right)\mathrm{CpdI}+S+H^{+}\underset{\to}{k_{1}}\mathrm{CpdII}+S^{\cdot+}$ (S20a)

($III) CpdII+S+H^{+}\underset{\to}{k_{2}}E_{\mathrm{red}}+S^{\cdot+}+H_{2}O$

Here $E_{\mathrm{red}}$ is the reduced enzyme, compound I (CpdI) is an intermediate oxidized by two electrons, and compound II (CpdII) is an intermediate reduced by one electron. It is known that reaction II is usually faster than reaction III [24–27]. Therefore, reaction III is a limiting step in substrate (*S*) oxidation and, in the case of one-electron redox couples, *S* follows a one-electron oxidation pathway to form a stable radical cation, which is not oxidized further and does not inhibit the reaction. The two-electron redox couples involve the oxidation of the substrate to form a radical cation, followed by subsequent disproportionation to form the fully oxidized product (the disproportionation reaction is diffusion-limited, at least for substrates MB and TH) [28]. Thus, in the case of two-electron redox couples, a second electron reduction potential is relevant.

The RP values of substrates were measured electrochemically in buffer solution at pH 7.00 by means of cyclic voltammetry (Table 1). The cyclic voltammograms (CVs) of DCPIP are illustrated as an example in Figure S3A. In this particular case, a two-electron redox couple is indicated as I and a one-electron irreversible oxidation to form a radical cation is indicated as II. Four of the substrates used in the research (TH, MB, DCPIP(I) and VB) are two-electron redox couples, which exhibit electrochemical two-electron redox processes.


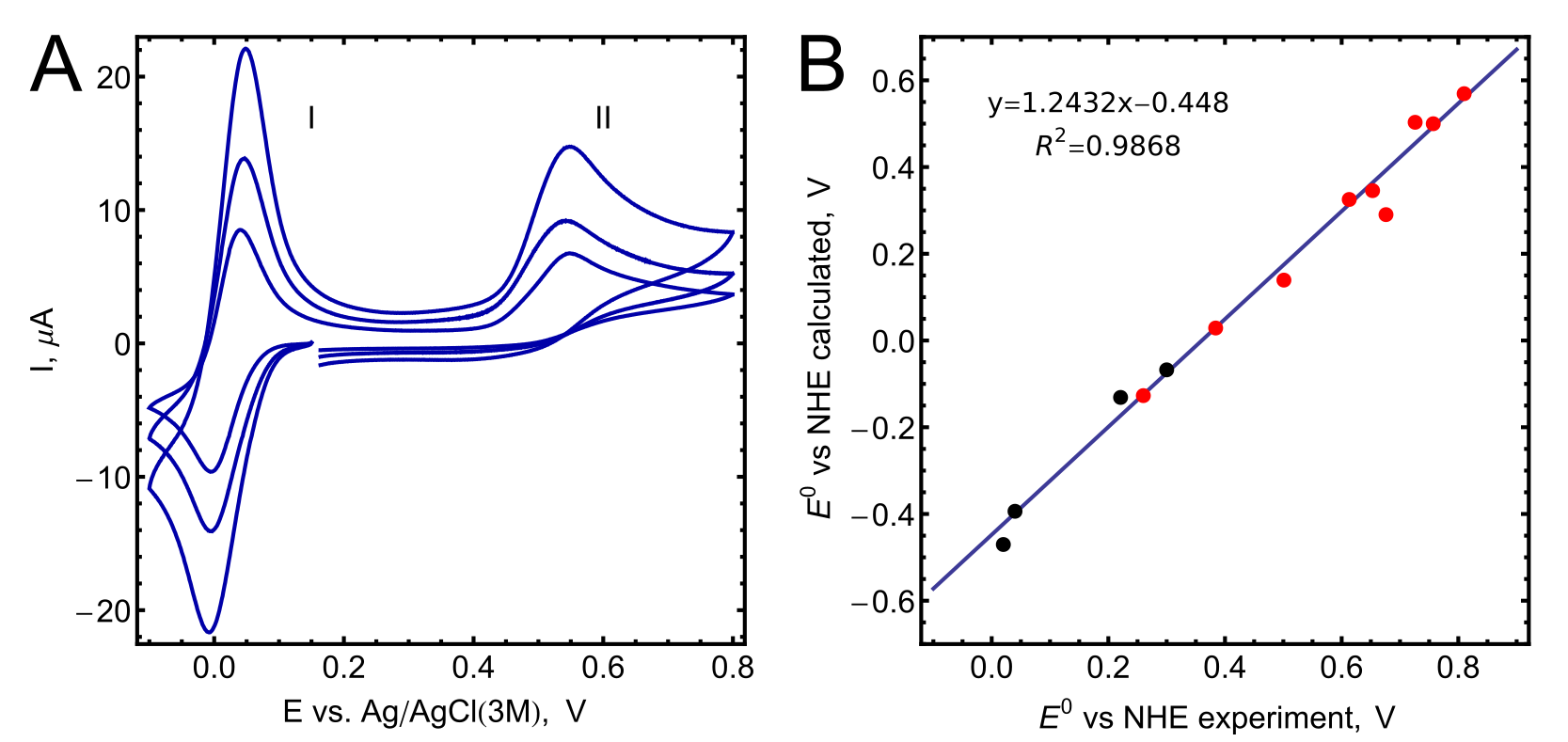


Figure S4. A) Cyclic voltammetry of DCPIP (1 mM) on a glassy carbon electrode in 50 mM phosphate buffer solution at pH 7.00 and 25 °C. B) Reduction potentials, calculated by quantum-chemical methods (*cf.* SI) vs. those measured experimentally. Red dots indicate one-electron and black dots indicate two-electron redox couples. The offset of theoretical RP values are due to the tendency of the DFT-B3LYP method to under-estimate RPs [29].

For these two-electron redox substrates, $E_{s}^{1}$ was estimated by using quantum chemical calculations with the DFT-B3LYP hybrid functional, the 6-31+G(d, p) basis set, and the solvation model density solvent model [12–16]. It was performed by calculating the theoretical RP for all substrates regardless of the number of electron involved. The RP of one-electron redox couples is represented by the following equation:

$S_{\mathrm{ox}}+e\leftrightarrow S_{\mathrm{red}}, E_{s}^{1}$ (S20b)

Therefore, the theoretical $E_{s}^{1}$ for substrate *S* is calculated from the energy difference between reduced ($S_{\mathrm{red}}$) and oxidized ($S_{\mathrm{ox}}$) forms of the substrate (additional details are in relevant SI sections). Similarly, the two-electron redox couples are represented by two separate one-electron reductions, as follows:

$S_{\mathrm{ox}}+e\leftrightarrow S_{\mathrm{red}}^{1}, E_{s}^{2}$

$S_{\mathrm{red}}^{1}+e\leftrightarrow S_{\mathrm{red}}, E_{s}^{1}$ (S20c)

The relevant RP for all the substrates in CpdII-catalyzed oxidation is $E_{s}^{1}$ (as discussed in the Introduction). However, here a two-electron process exhibits a single-wave-peak electrochemical behavior similar to a one-electron process with $E_{s}^{0}={(E}_{s}^{2}+E_{s}^{1})/2$ [10]. Therefore, both $E_{s}^{1}$ and $E_{s}^{2}$ were calculated in order to estimate $E_{s}^{0}$ for two-electron redox couples, and to compare the calculations with experimental data. It was found that theoretical RP values correlate well with RP values measured electrochemically (Figure 4B). Therefore, a linear regression (*cf.* equation in Figure 4B) was used to estimate $E_{s}^{1}$ from theoretically calculated values of one-electron reduction potentials of TH, MB, DCPIP(I) and VB; the results are presented in Table 1 in the manuscript.

# 5. Proton transfer rate constant

The multistate nonadiabatic rate constant of proton transfer is described as [30]:

$k_{p}=\sum_{n} \sum_{m} P_{n}\frac{V_{\mathrm{nm}}^{2}}{\hbar}\sqrt{\frac{\pi}{\lambda_{p}k_{B}T}}e\left( \frac{-\Delta G_{\mathrm{nm}}^{\ddagger}}{k_{B}T} \right)$ (S21)

$\Delta G_{\mathrm{nm}}^{\ddagger}=\frac{{(\Delta G_{p}+E_{m}-E_{n}+\lambda_{p})}^{2}}{4\lambda_{p}}$ (S22)

Here $P_{n}$ is probability (from Boltzmann distribution) of transition from n^th^ reactant state, $V_{\mathrm{nm}}$ vibrational coupling between reactant and product wavefunctions, $\lambda_{p}$ reorganization energy of proton transfer; $E_{n}$ and $E_{m}$ energies of reactants and products, respectively, in states $n$ and $m$. The energies and integrals of overlap can be calculated by using approximation of harmonic oscillator potential with resulting wave functions [31]:

$\psi_{n}(x)=\frac{e^{\frac{-1}{2}x^{2}\sqrt{\frac{km_{p}}{\hbar^{2}}}}\sqrt{\frac{2^{-n}\frac{\sqrt[4]{km_{p}}}{\hbar^{2}}}{n!}}H_{n}\left( x\frac{\sqrt[4]{km_{p}}}{\hbar^{2}} \right)}{\sqrt[4]{\pi}}$ (S23)

Where $k$ is a force constant associated with reorganization energy ($k=2\lambda\text{/}\Delta x^{2}$, we assume, that [32]collective reaction coordinate can be approximated using physical proton transfer coordinate and the variation of $\lambda$ accounts solvent reorganization energy associated with collective behavior of medium) and $m_{p}$ is a mass of proton. The vibronic coupling integrals are calculated as [32]:

$V_{\mathrm{nm}}=\frac{H_{nm}-\frac{S_{nm}(H_{nn}+H_{mm})}{2}}{(1-S_{nm})^{2}}$ (S24)

Where $S_{nm}$ is overlap integral between wave functions $\psi_{n}(x)$ and $\psi_{m}(-x+\Delta x)$ ($S_{nm}=\left\langle\psi_{n}(x)|\psi_{m}(-x+\Delta x) \right\rangle$). The matrix elements are calculated as:

$H_{nm}=\left\langle\psi_{n}(x)|\overset{ˆ}{H}|\psi_{m}(-x+\delta x) \right\rangle$ (S25)

The Hamiltonian for the system is:

$\overset{ˆ}{H}=\frac{-\hbar^{2}}{2m}\nabla^{2}+\frac{k}{2}x^{2}+\frac{k}{2}(x-\Delta x)^{2}$ (S26)

#

# 6. Oxidation rate constants and kinetic isotope effect

Table S2. Bimolecular rate constants (steady state) of ET-PT in CIP-catalyzed oxidation (CpdII reduction) of substrates (at 25 °C), measured in water and deuterium oxide; predicted kinetic isotope effects (KIE_pred_) and average of substrate concentrations used in calculations of rate constants.

| **Substrate** | ***k*_app_, M^-1^s^-1^, measured** | ***k*_e_, M^-1^s^-1^, calculated** | ***k*^H^_2_^O^_app_,**  **M^-1^s^-1^, calculated** | ***k*^D^_2_^O^_app_,**  **M^-1^s^-1^, calculated** | **KIE_pred_** | **[*S*]_av_, M** |
| --- | --- | --- | --- | --- | --- | --- |
| ABTS | 3.8±0.1 × 10^7^ | 3.3±0.6 × 10^7^ | 1.7±0.1 × 10^7^ | 1.25±0.08 × 10^7^ | 1.4±0.1 | 1.47 × 10^−5^ |
| AMB | 1.003±0.003 × 10^7^ | 6.0±0.8 × 10^7^ | 1.05±0.07 × 10^7^ | 6.5±0.4 × 10^6^ | 1.6±0.2 | 4.03 × 10^−5^ |
| CPZ | 3.1±0.2 × 10^6^ | 8±1 × 10^5^ | 8±1 × 10^5^ | 8±1 × 10^5^ | 1.0±0.1 | 1.28 × 10^−5^ |
| DCPIP(I) | 3.8±0.8 × 10^8^ | 7.0±0.9 × 10^8^ | 2.2±0.1 × 10^8^ | 1.5±0.1 × 10^8^ | 1.5±0.1 | 1.56 × 10^−6^ |
| DCPIP(II) | 1.3±0.2 × 10^7^ | 1.3±0.1 × 10^8^ | 9.1±0.7 × 10^7^ | 7.6±0.5 × 10^7^ | 1.2±0.1 | 1.56 × 10^−6^ |
| DMB | 2.3±0.1 × 10^6^ | 1±0.1 × 10^8^ | 3.8±0.3 × 10^7^ | 2.5±0.2 × 10^7^ | 1.5±0.1 | 8.51 × 10^−6^ |
| HEPX | 1.56±0.01 × 10^8^ | 3.5±0.4 × 10^7^ | 1.3±0.1 × 10^7^ | 8.7±0.6 × 10^6^ | 1.5±0.1 | 2.52 × 10^−5^ |
| MB | 2.66±0.03 × 10^8^ | 7±1 × 10^8^ | 3.3±0.3 × 10^8^ | 2.4±0.2 × 10^8^ | 1.4±0.1 | 8.12 × 10^−7^ |
| PPSA | 2.37±0.03 × 10^8^ | 1.0±0.1 × 10^8^ | 5.0±0.3 × 10^7^ | 3.6±0.2 × 10^7^ | 1.4±0.1 | 5.29 × 10^−6^ |
| PZ | 3.40±0.05 × 10^6^ | 8±0.8 × 10^6^ | 6.7±0.6 × 10^6^ | 6.0±0.5 × 10^6^ | 1.12±0.03 | 1.19 × 10^−5^ |
| TH | 4.80±0.07 × 10^8^ | 5.0±0.6 × 10^8^ | 3.4±0.3 × 10^8^ | 2.8±0.2 × 10^8^ | 1.2±0.1 | 4.70 × 10^−7^ |
| TMPD | 1.76±0.01 × 10^7^ | undefined | 3.1±0.2 × 10^7^ | 1.8±0.1 × 10^7^ | 1.8±0.2 | 1.68 × 10^−5^ |
| VB | 2.56±0.03 × 10^7^ | 1.4±0.1 × 10^7^ | 1.4±0.1 × 10^7^ | 9.9±0.6 × 10^6^ | 1.5±0.1 | 2.19 × 10^−5^ |

Experimentally, KIE values were measured for: CpdI formation (1.33±0.05), oxidation of TMPD (1.6±0.2), CPZ (1.0±0.1), and ABTS (1.3±0.1) with CIP. The same was done for CpdI formation (1.6±0.2), oxidation of TMPD (1.9±0.3), CPZ (1.0±0.2), and ABTS (1.0±0.1) with HRP. The KIE were measured under steady state conditions. Additional details regarding KIE measurement are provided further.

Table S3. Kinetic (steady state) and thermodynamic parameters of HRP-catalyzed substrate oxidation (CpdII reduction).

| Substrate | *k*_2_, M^−1^ s^− 1^, at 25 *C*^○^ | $E_{s}^{1}$, V vs. NHE | | *λ_s_*, kcal/mol^a^ | | ln *A* | | Δ*G*^‡^, kcal/mol | | *θ*_s_^b^ | |
| --- | --- | --- | --- | --- | --- | --- | --- | --- | --- | --- | --- |
| ABTS | 1.12±0.03 × 10^5^ | 0.686±0.006 | 3.42 | | 22.±1. | | 6.2±0.6 | | 0.44 | |  |
| AMB | 3.64±0.04 × 10^7^ | 0.394±0.005 | 11.17 | | 34±2 | | 9.7±1.0 | | 0.34 | |  |
| CPZ | 1.2±0.1 × 10^5^ | 0.79±0.01 | 12.04 | | 22±2 | | 6.1±0.7 | | 0.46 | |  |
| DCPIP(I) | 2.10±0.04 × 10^8^ | 0.568^c^ | 12.47 | | 33±1 | | 7.9±0.6 | | 0.36 | |  |
| DCPIP(II) | 1.9±0.5 × 10^6^ | 0.736±0.01 | 3.77 | | 22.4±0.4 | | 4.7±0.9 | | 0.37 | |  |
| DMB | 7.8±0.6 × 10^6^ | 0.511±0.004 | 11.04 | | 34±1 | | 10.6±0.8 | | 0.38 | |  |
| HEPX | 4.3±0.2 × 10^7^ | 0.663±0.003 | 3.10 | | 28±1 | | 6±0.6 | | 0.35 | |  |
| MB | 2.01±0.04 × 10^8^ | 0.162^c^ | 9.12 | | 32±1 | | 7.6±0.6 | | 0.37 | |  |
| PPSA | 4.82±0.04 × 10^7^ | 0.623±0.003 | 2.65 | | 30±1 | | 7.2±0.8 | | 0.33 | |  |
| PZ | 8.24±0.1 × 10^5^ | 0.767±0.006 | 11.82 | | 22.6±1.6 | | 5.3±1.0 | | 0.43 | |  |
| TH | 2.80±0.05 × 10^8^ | 0.250^c^ | 8.85 | | 30.6±0.4 | | 6.6±0.2 | | 0.34 | |  |
| TMPD | 4.6±0.2 × 10^7^ | 0.270±0.006 | 11.68 | | 34±1 | | 9.7±0.8 | | 0.32 | |  |
| VB | 1.23±0.05 × 10^8^ | 0.369^c^ | 8.15 | | 30±1 | | 6.9±0.5 | | 0.36 | |  |

^a^ Theoretically calculated self-exchange (inner and solvent) reorganization energies; b ratio of solvent-accessible surface in a docked enzyme-substrate complex; c values calculated theoretically based on one-electron RP using the linear regression equation shown in Figure S4B.

# 7. Kinetics, thermodynamics and electrochemistry

## 7.1. Activation energy of compound I formation and kinetic isotope effect

For the formation of CpdI from reduced CIP form the following scheme is postulated:

$\begin{matrix} E_{\mathrm{red}}+H_{2}O_{2}\overset{k_{H_{2}O_{2}}}{\to}CpdI \end{matrix}$ (S27)

Measurements were recorded at 405 nm wavelength and experiments were repeated for at least 10 times. Mean absorbance change time course was fitted directly into reaction scheme (equation (S27)) using molar absorption coefficients 109 and 66 mM^-1^cm^-1^, for reduced and oxidized enzyme forms, respectively. Initial concentrations of enzyme were calculated from the first point of kinetic curve (~0.850 µM), concentration of hydrogen peroxide was 4.09 µM. Experiments were performed in 50 mM phosphate buffer solution at pH 7.00; the results are presented in Table S4 and Figure S5. The rate constant of CpdI formation in D_2_O at 25 °C were measured using the same enzyme and substrates’ concentrations in 50 mM phosphate buffer solution at pD 7.00. The same concentrations and procedures were also used for HRP.

Table S4. Apparent bimolecular rate constants of CpdI formation measured at different temperatures.

| *t*, °C | Apparent bimolecular rate  constants (CIP), M^-1^s^-1^ | *t*, °C | Apparent bimolecular rate  constants (HRP), M^-1^s^-1^ |
| --- | --- | --- | --- |
| 15 | 7.12±0.09 × 10^6^ | 10 | 8.5±0.09 × 10^6^ |
| 17.5 | 7.32±0.06 × 10^6^ | 15 | 9.0±0.1 × 10^6^ |
| 20 | 7.9±0.2 × 10^6^ | 20 | 9.9±0.2 × 10^6^ |
| 22.5 | 8.05±0.07 × 10^6^ | 25 | 1.13±0.08 × 10^7^ |
| 25 | 8.8±0.1 × 10^6^ | 30 | 1.2±0.1 × 10^7^ |
| 27.5 | 9.3±0.2 × 10^6^ |  |  |


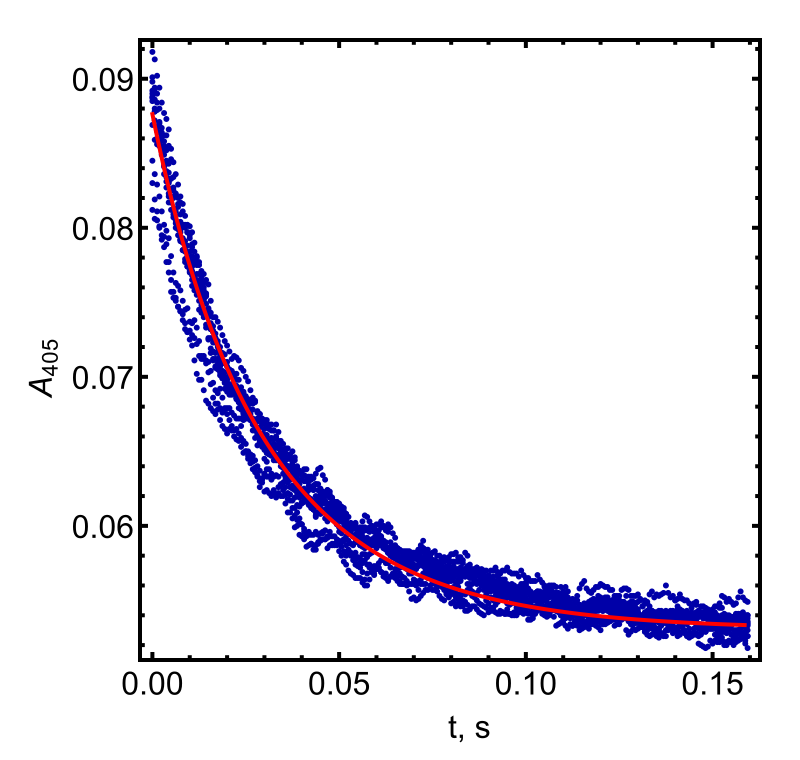

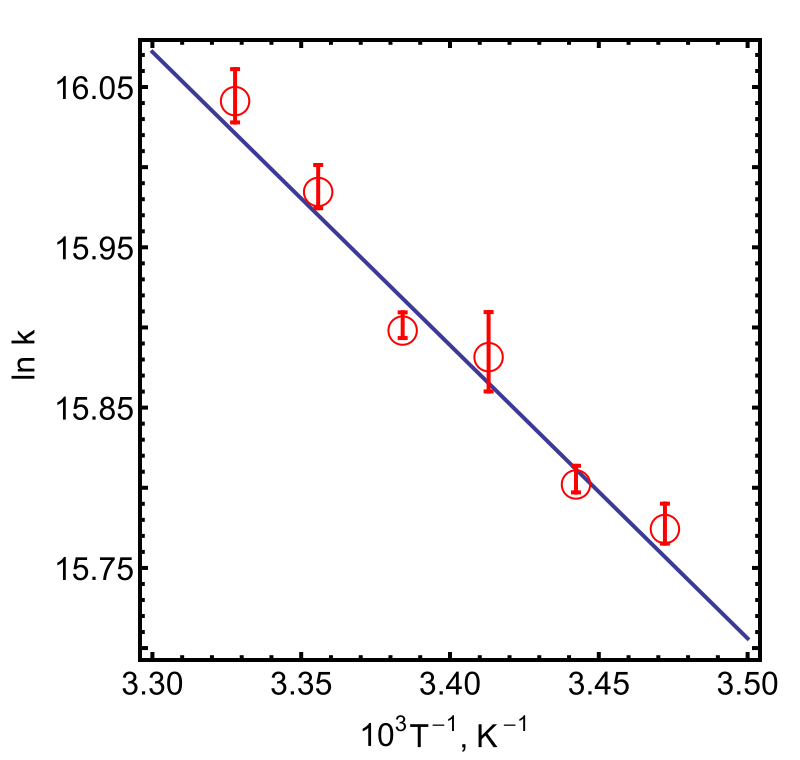

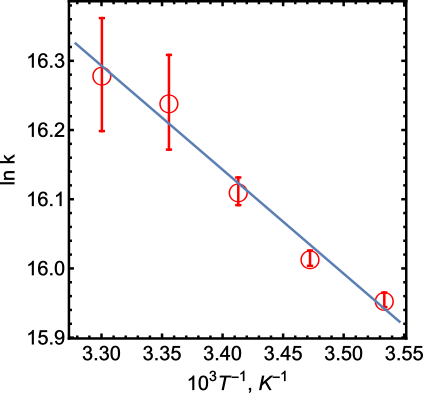


Figure S5. Kinetic curves of CpdI formation (25 °C) (*left*) and the logarithmic correlation of apparent reaction rate constants with temperature (*middle*) for CIP and (right) for HRP.

From the experimental results, the free activation energies were calculated as 3.6±1.0 kcal/mol and 3.0±0.3 kcal/mol, and the pre-exponential factors were found to be 4±18×10^9^ M^-1^s^-1^ and 1±3×10^9^ M^-1^s^-1^ for CIP and HRP, respectively.

For further calculations, the apparent rate constant of CpdI formation at certain temperature was estimated as:

$k_{H_{2}O_{2},CIP}=e^{22.1087-\frac{1829.34}{T}} and k_{H_{2}O_{2},HRP}=e^{21.2519-\frac{1502.7}{T}}$ (S28)

The calculated kinetic isotope effect for CpdI formation is 1.33±0.05 for CIP and 1.6±0.1 for HRP.

## 7.2. Activation energy of HEPX oxidation and its reduction potential

The reaction scheme of HEPX oxidation is postulated as:

$\begin{aligned} E_{\mathrm{red}}+H_{2}O_{2}\overset{k_{H_{2}O_{2}}}{\to}CpdI \\ CpdI+S\underset{k_{-1}}{\overset{k_{1}}{\rightleftharpoons}}CpdI\cdot S \\ CpdI\cdot S\overset{k_{\mathrm{cat},1}}{\to}CpdII+S^{\cdot+} \\ CpdII+S\underset{k_{-2}}{\overset{k_{2}}{\rightleftharpoons}}CpdII\cdot S \\ \begin{matrix} CpdII\cdot S\overset{k_{\mathrm{cat},2}}{\to}E_{\mathrm{red}}+S^{\cdot+} \end{matrix} \end{aligned}$ (S29)

In the fitting process $k_{H_{2}O_{2}}$ was fixed to an apparent CpdI formation rate constant for a relevant temperature (Equation (S28)). Limiting apparent bimolecular rate constant calculated from:

$k_{\lim}=\frac{k_{\mathrm{cat}}}{K_{M}},\mathrm{where}k_{\mathrm{cat}}=\frac{k_{\mathrm{cat},1}k_{\mathrm{cat},2}}{k_{\mathrm{cat},1}+k_{\mathrm{cat},2}} \mathrm{and}K_{M}=\frac{k_{\mathrm{cat},1}K_{M,2}+K_{M,1}k_{\mathrm{cat},2}}{k_{\mathrm{cat},1}+k_{\mathrm{cat},2}}$ (S30)

$K_{M,n}\to\frac{k_{\mathrm{cat},n}+k_{-n}}{k_{n}}.$ (S31)

Where $k_{\lim}$ calculated from fitted $k_{\mathrm{cat}}$ and $K_{M}$. The initial concentrations of HEPX varied from 6.3 to 48 µM, and the initial concentration of CIP was 4.84 nM. Experimental results are presented in Table S5 and Figure S6, where $k_{\lim}$ is calculated from fitted $k_{\mathrm{cat}}$ and $K_{M}$. The initial concentrations of HEPX varied from 2 to 31 µM, and the initial concentration of HRP was 93 nM. Experimental results are presented in Table S5 and Figure S6.

Table S5. Apparent bimolecular rate constants of HEPX oxidation measured at different temperatures.

| *t*, °C | Apparent bimolecular rate  constant (CIP), M^-1^s^-1^ |  | *t*, °C | Apparent bimolecular rate  constant (HRP), M^-1^s^-1^ |
| --- | --- | --- | --- | --- |
| 11 | (9.3±0.2) × 10^7^ |  | 10 | (2.4±0.1) × 10^7^ |
| 15 | (9.9±0.6) × 10^7^ |  | 15.1 | (3.1±0.2) × 10^7^ |
| 20 | (1.24±0.01) × 10^8^ |  | 20 | (3.3±0.3) × 10^7^ |
| 25 | (1.56±0.01) × 10^8^ |  | 25 | (4.3±0.2) × 10^7^ |
| 30 | (1.82±0.02) × 10^8^ |  | 30.1 | (4.3±0.4) × 10^7^ |


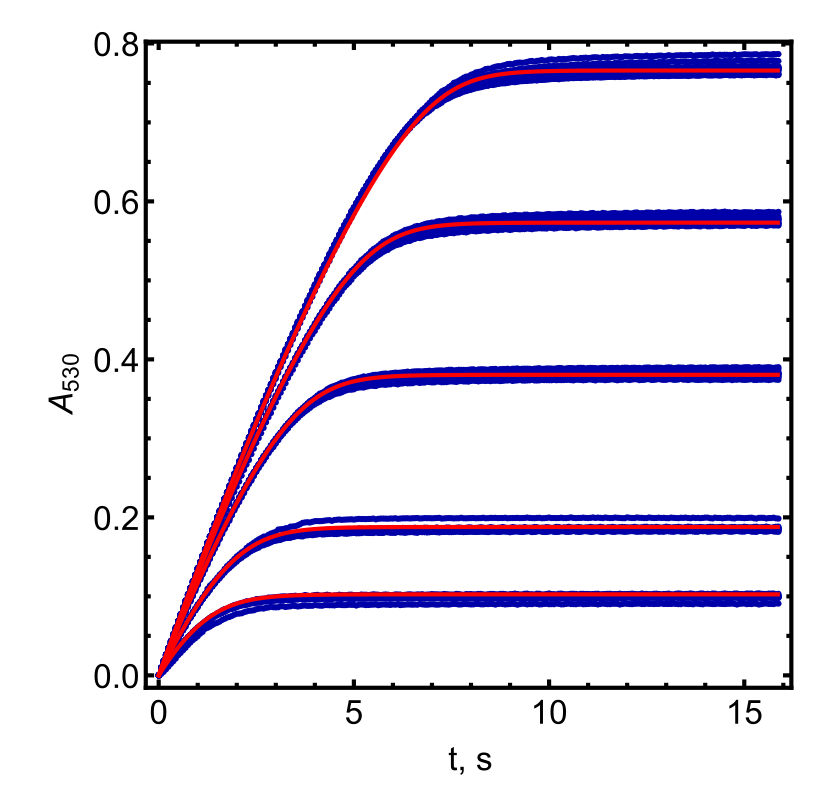

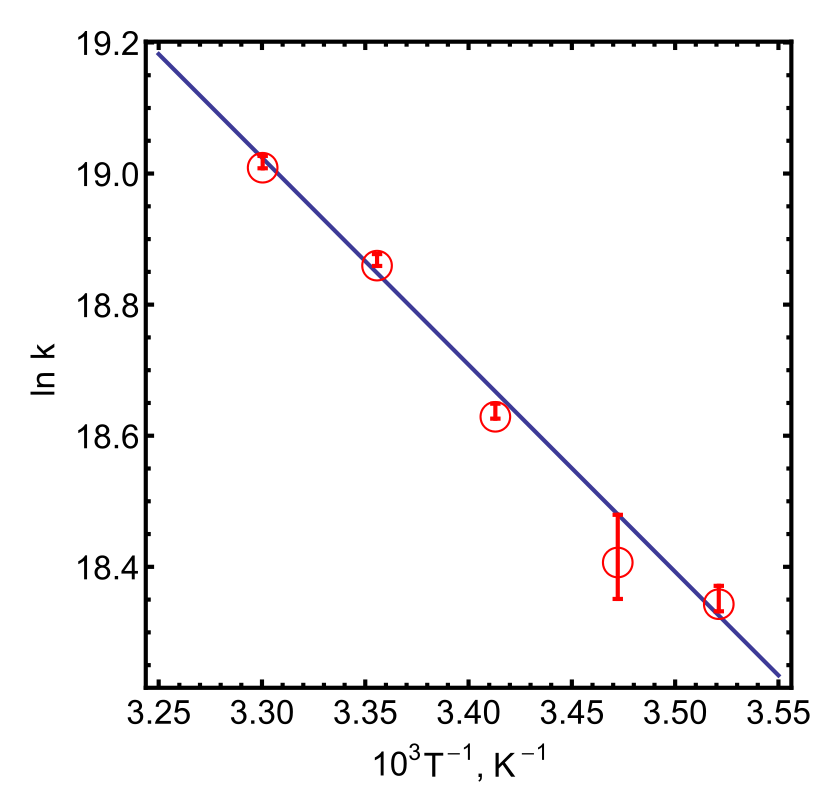

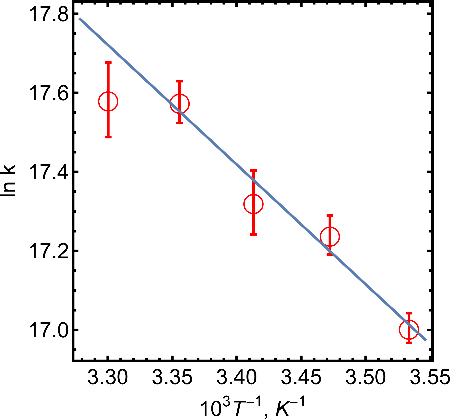


Figure S6. Kinetic curves of HEPX oxidation (25 °C) (*left*) and the logarithmic correlation of apparent reaction rate constants with temperature for CIP (*middle*) and HRP (*right*).

From the experimental results, the free activation energies were calculated as 6.2±1.2 kcal/mol, and 6.0±0.6 kcal/mol, as well as the pre-exponential factors were found to be 6±42 × 10^12^ M^-1^s^-1^ and 2±2 × 10^12^ M^-1^s^-1^ for CIP and HRP, respectively.

Due to limited HEPX solubility in water, CV experiments for determination of the HEPX reduction potential were done by mixing 10 µl of 6.38 mM HEPX solution in acetonitrile with 10 µl of deionized water and dropping the mixture on a clean glassy carbon electrode. Then, the drop on the surface was allowed to evaporate, with HEPX remaining adsorbed on electrode. CV measurements were performed in 50 mM phosphate buffer solution, pH 7.00, t – 25 °C (Figure S7). For each CV experiment, a freshly prepared HEPX-adsorbed electrode was used.


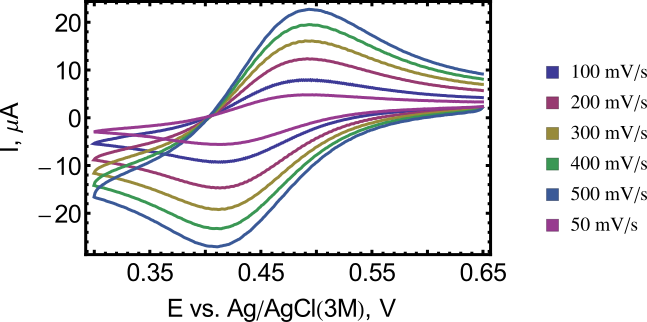


Figure S7. CVs of HEPX adsorbed on the glassy carbon working electrode (potential sweep rates are presented in the right panel).

The reduction potential was estimated to be 0.453±0.003 V vs. Ag/AgCl(3 M).

## 7.3. Activation energy of ABTS oxidation, its reduction potential and kinetic isotope effect

The reaction scheme of ABTS oxidation is postulated as:

$\begin{aligned} E_{\mathrm{red}}+H_{2}O_{2}\overset{k_{H_{2}O_{2}}}{\to}CpdI \\ CpdI+S\underset{k_{-1}}{\overset{k_{1}}{\rightleftharpoons}}CpdI\cdot S \\ CpdI\cdot S\overset{k_{\mathrm{cat},1}}{\to}CpdII+S^{\cdot+} \\ CpdII+S\underset{k_{-2}}{\overset{k_{2}}{\rightleftharpoons}}CpdII\cdot S \\ \begin{matrix} CpdII\cdot S\overset{k_{\mathrm{cat},2}}{\to}E_{\mathrm{red}}+S^{\cdot+} \end{matrix} \end{aligned}$ (S32)

The rate constants were calculated as for the HEPX oxidation experiments, according to Equations S30-S31. In the case of CIP measurements, the initial concentrations of ABTS were varied from 3.2 to 27 µM, and the initial concentration of the enzyme was 14.8 nM. The experimental results are presented in Table S6 and Figure S8. In the case of HRP studies, the initial concentrations of ABTS were varied from 26 to 209 µM, and initial concentration of the enzyme was 29 nM. Experimental results are presented in Table S6 and Figure S8. The limiting rate constants of ABTS oxidation in D_2_O at 25 °C were measured using the H_2_O concentrations of enzymes and substrates in 50 mM phosphate buffer solution at pD 7.00.

Table S6. Apparent bimolecular rate constants of ABTS oxidation measured at different temperatures.

| *t*, °C | Apparent bimolecular rate  Constant (CIP), M^-1^s^-1^ | *t*, °C | Apparent bimolecular rate  Constant (HRP), M^-1^s^-1^ |
| --- | --- | --- | --- |
| 10.2 | (2.36±0.04) × 10^7^ | 10.1 | (5.8±0.4) × 10^4^ |
| 15 | (2.81±0.07) × 10^7^ | 15.2 | (7.5±0.6) × 10^4^ |
| 20 | (3.45±0.08) × 10^7^ | 20.1 | (9.5±0.5) × 10^4^ |
| 25 | (3.8±0.1) × 10^7^ | 25.2 | (1.12±0.03) × 10^5^ |
| 30 | (4.3±0.1) × 10^7^ | 30.2 | (1.34±0.02) × 10^5^ |


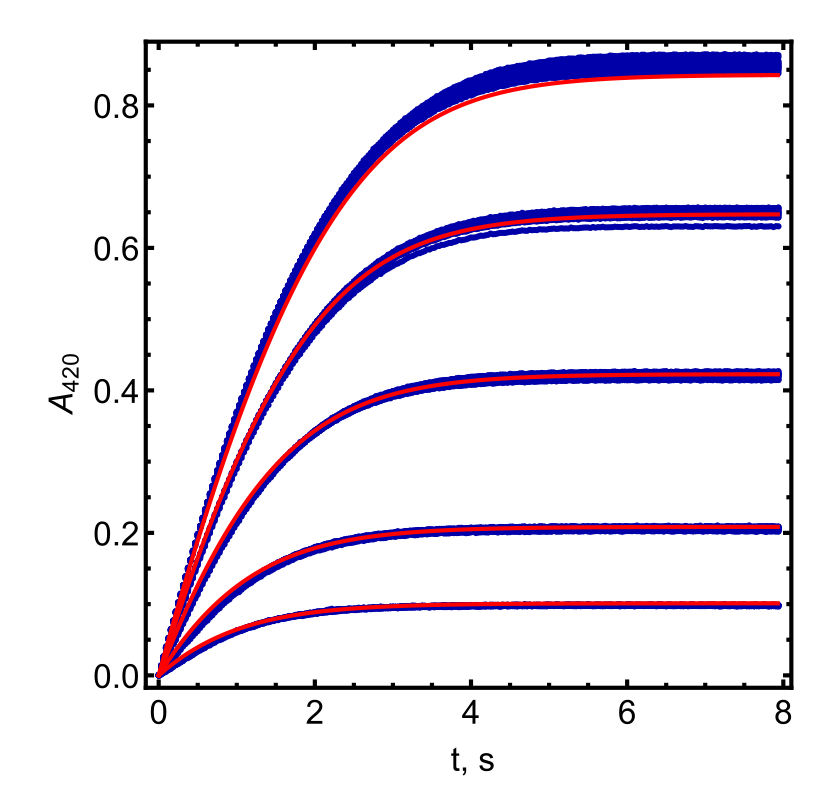

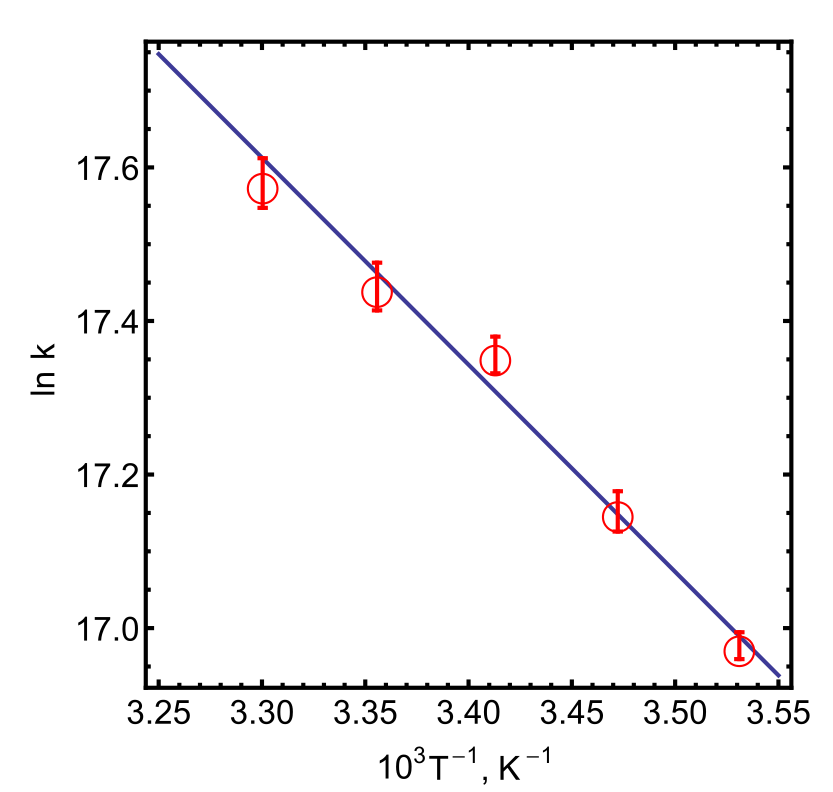

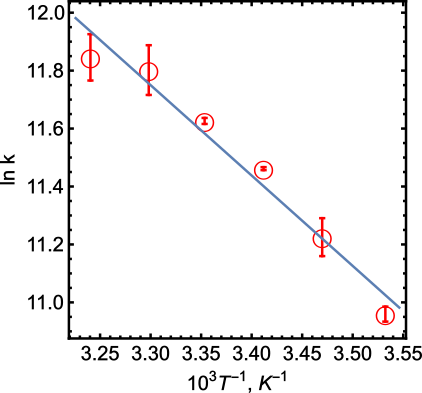


Figure S8. Kinetic curves of ABTS oxidation (25 °C) (*left*) and the logarithmic correlation of apparent reaction rate constants with temperature for CIP (*middle*) and HRP (*right*).

From the experimental results, the free activation energies were calculated as $\text{5.5±1.2 kcal}\text{/}\text{mol}$, and $\text{6.2±1.2 kcal}\text{/}\text{mol}$, and the pre-exponential factors were found to be$\text{3±22×}\text{10}^{\text{11}}\text{M}^{\text{-1}}\text{s}^{\text{-1}}$ and $\text{4±6×}\text{10}^{\text{9}}\text{M}^{\text{-1}}\text{s}^{\text{-1}}$ for CIP and HRP, respectively. The calculated kinetic isotope effects for ABTS oxidation with CpdII from CIP and HRP were 1.3±0.1 and 1.0±0.1, respectively.

CV experiments for determination of the ABTS reduction potential were carried out by using a clean glassy carbon electrode at ABTS concentration of 1 mM. CV measurements were performed in 50 mM phosphate buffer solution at pH 7.00, 25 °C (Figure S9).


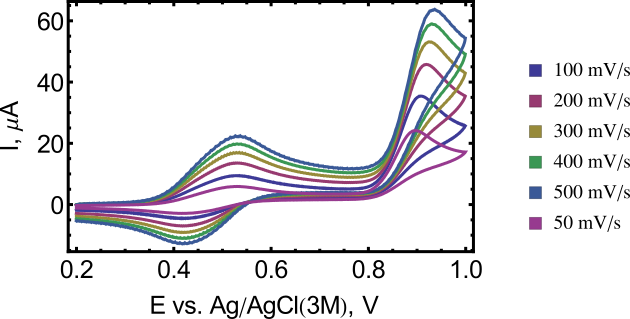


Figure S9. CVs of ABTS on the glassy carbon working electrode (potential sweep rates are presented in the right panel).

The first-electron reduction potential was estimated to be $\text{0.476±0.006 V vs. Ag}\text{/}\text{AgCl(3M)}$.

## 7.4. Activation energy of AMB oxidation and its reduction potential

The reaction scheme of AMB oxidation is postulated as:

$\begin{aligned} E_{\mathrm{red}}+H_{2}O_{2}\overset{k_{H_{2}O_{2}}}{\to}CpdI \\ CpdI+S\overset{k_{1}}{\to}CpdII+S^{\cdot+} \\ \begin{matrix} CpdII+S\overset{k_{2}}{\to}E_{\mathrm{red}}+S^{\cdot+} \end{matrix} \end{aligned}$ (S33)

$S^{\cdot+} + S^{\cdot+}\overset{k_{\mathrm{dis}}}{\to}S+S_{\mathrm{ox}}$

The limiting apparent bimolecular rate constants were calculated as $k_{\lim}=\frac{k_{1}k_{2}}{k_{1}+k_{2}}$. The initial concentrations of AMB were varied from 9.6 to 70 µM for CIP, and the initial concentration of the enzyme was 18 nM. Experimental results are presented in Table S7 and Figure S10. The initial concentrations of AMB were varied from 4 to 51 µM for HRP, and the initial concentration of the enzyme was 200 nM. Experimental results are presented in Table S7 and Figure S10.

Table S7. Apparent bimolecular rate constants of AMB oxidation measured at different temperatures.

| *t*, °C | Apparent bimolecular rate  constant (CIP), M^-1^s^-1^ | *t*, °C | Apparent bimolecular rate  constant (HRP), M^-1^s^-1^ |
| --- | --- | --- | --- |
| 11 | (4.434±0.001) × 10^6^ | 10 | (1.45±0.09) × 10^7^ |
| 15 | (5.555±0.001) × 10^6^ | 15 | (2.57±0.07) × 10^7^ |
| 20 | (7.158±0.001) × 10^6^ | 19.8 | (2.97±0.03) × 10^7^ |
| 25 | (1.003±0.003) × 10^7^ | 25 | (3.64±0.04) × 10^7^ |
| 30 | (1.263±0.004) × 10^7^ | 30 | (5.02±0.04) × 10^7^ |


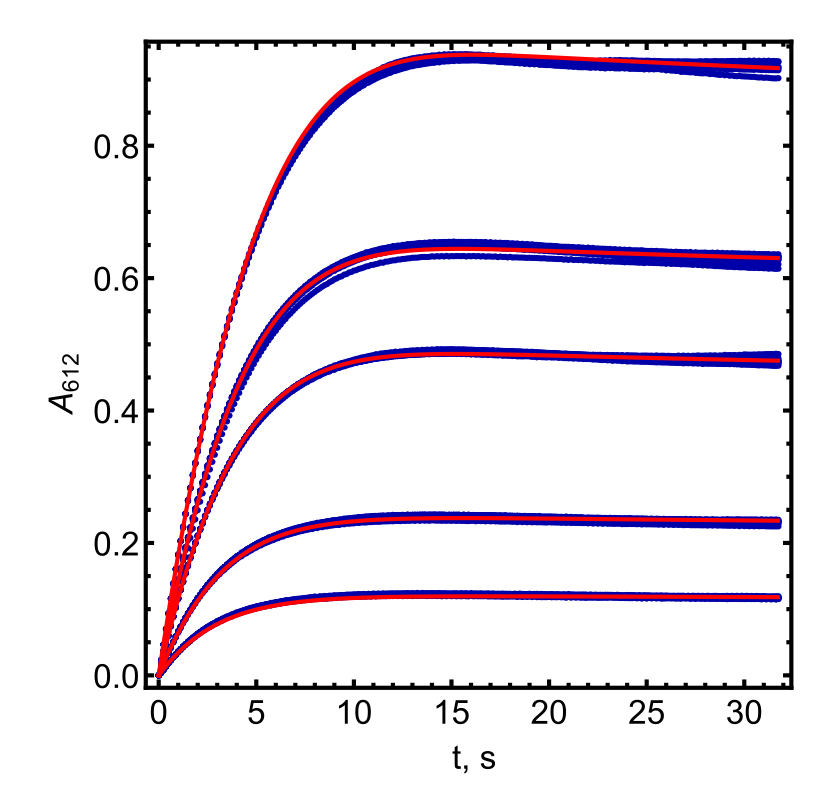

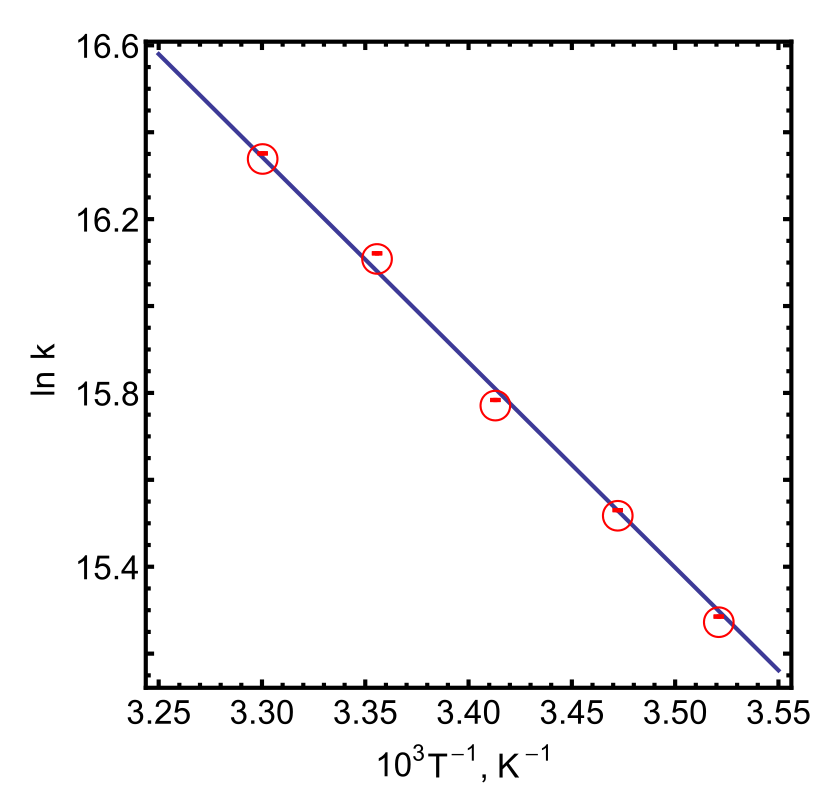

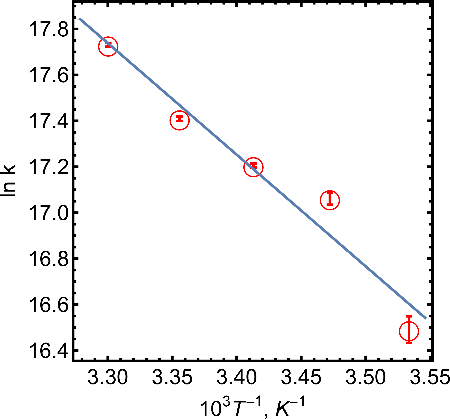


Figure S10. Kinetic curves of AMB oxidation (25 °C) (*left*) and the logarithmic correlation of apparent reaction rate constants with temperature for CIP (*middle*) and HRP (*right*).

From the experimental results, the free activation energies were calculated as $\text{9.1±1.1 kcal}\text{/}\text{mol and}$ $\text{10±1 kcal}\text{/}\text{mol}$, and the pre-exponential factors were found to be$\text{7±37×}\text{10}^{\text{13}}{\text{ }\text{M}}^{\text{-1}}\text{s}^{\text{-1}}$ and $\text{6±37×}\text{10}^{\text{14}}{\text{ }\text{M}}^{\text{-1}}\text{s}^{\text{-1}}$ for CIP and HRP, respectively.

CV experiments for determination of the AMB reduction potential were carried out using the gold electrode, cleaned according to procedure described before, at a AMB concentration of 1 mM. CV measurements were performed in 50 mM phosphate buffer solution at pH 7.00, 25 °C (Figure S11).


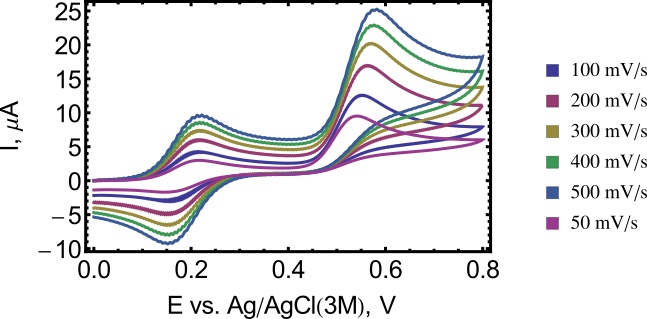


Figure S11. CVs of AMB on the gold working electrode (potential sweep rates are presented in the right panel).

The first-electron reduction potential was estimated to be $\text{0.184±0.005}\text{ }\text{V vs. Ag}\text{/}\text{AgCl(3M)}$.

## 7.5. Activation energy of PZ oxidation and its reduction potential

The reaction scheme of PZ oxidation is postulated as:

$\begin{aligned} E_{\mathrm{red}}+H_{2}O_{2}\overset{k_{H_{2}O_{2}}}{\to}CpdI \\ CpdI+S\underset{k_{-1}}{\overset{k_{1}}{\rightleftharpoons}}CpdI\cdot S \\ CpdI\cdot S\overset{k_{\mathrm{cat},1}}{\to}CpdII+S^{\cdot+} \\ CpdII+S\underset{k_{-2}}{\overset{k_{2}}{\rightleftharpoons}}CpdII\cdot S \\ \begin{matrix} CpdII\cdot S\overset{k_{\mathrm{cat},2}}{\to}E_{\mathrm{red}}+S^{\cdot+} \end{matrix} \end{aligned}$ (S34)

$S^{\cdot+} + S^{\cdot+}\overset{k_{\mathrm{dis}}}{\to}S+S_{\mathrm{ox}}$

The rate constants were calculated as for the HEPX oxidation experiments, according to Equations S30-S31. The initial concentrations of PZ varied from 2.52 to 23 µM for CIP, and the initial concentration of the enzyme was 25 nM. Experimental results are presented in Table S8 and Figure S12. The initial concentrations of PZ varied from 29 to 234 µM for HRP, and the initial concentration of the enzyme was 33 nM. Experimental results are presented in Table S8 and Figure S12.

Table S8. Apparent bimolecular rate constants of PZ oxidation measured at different temperatures.

| *t*, °C | Apparent bimolecular rate  constant (CIP), M^-1^s^-1^ | *t*, °C | Apparent bimolecular rate  constant (HRP), M^-1^s^-1^ |
| --- | --- | --- | --- |
| 10 | (2.75±0.03) × 10^6^ | 10.1 | (5.0±0.5) × 10^5^ |
| 15 | (3.07±0.07) × 10^6^ | 15.1 | (6.7±0.3) × 10^5^ |
| 20 | (3.12±0.06) × 10^6^ | 20.1 | (7.6±0.8) × 10^5^ |
| 25 | (3.40±0.05) × 10^6^ | 25.2 | (8.3±0.9) × 10^5^ |
| 30 | (3.5±0.1) × 10^6^ | 30.2 | (9.8±0.7) × 10^5^ |


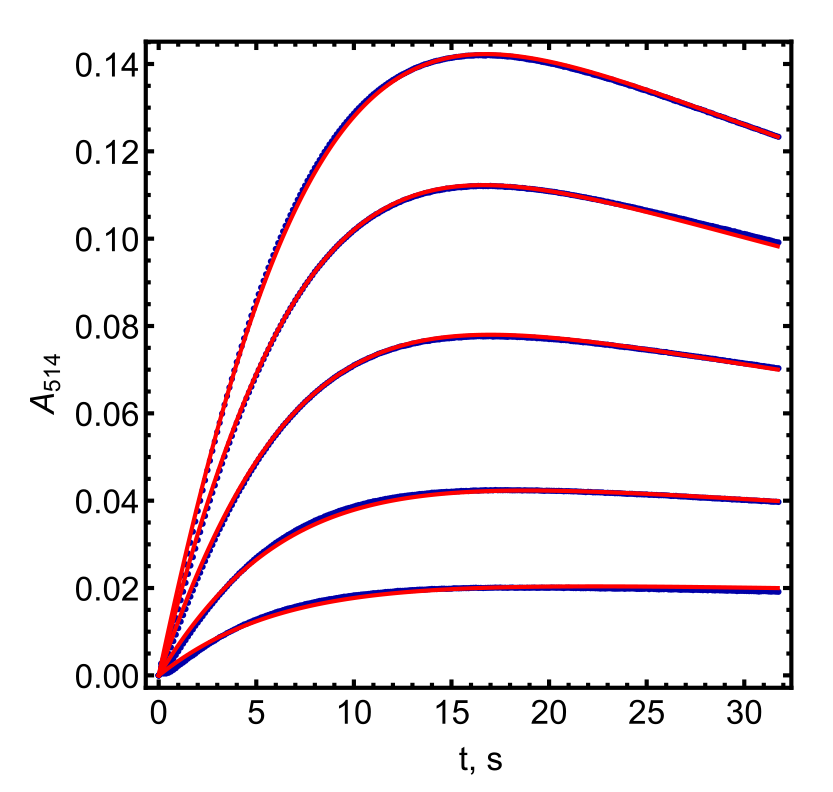

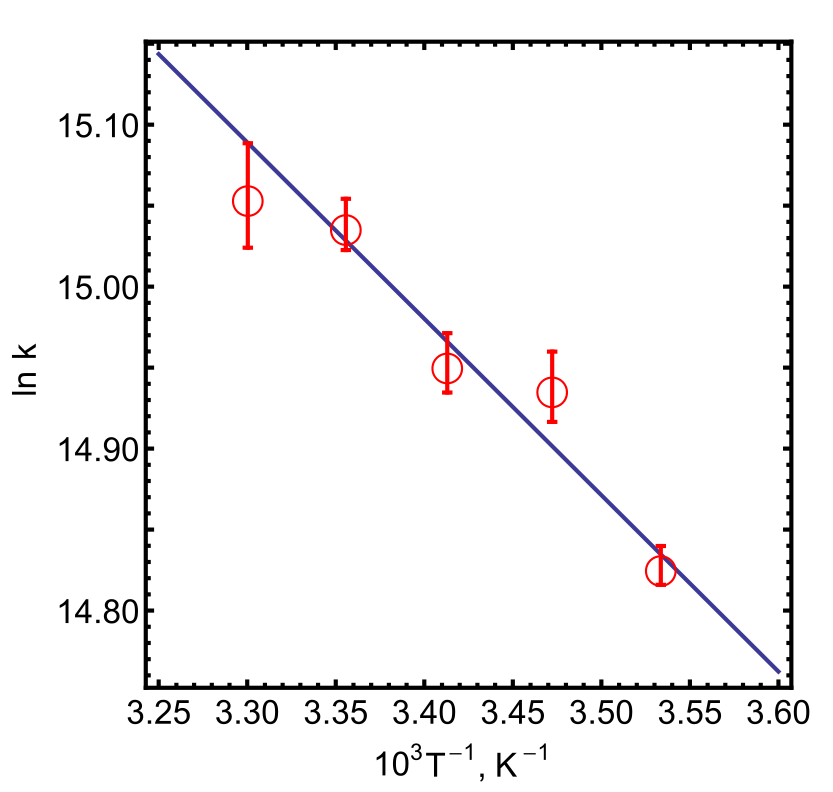

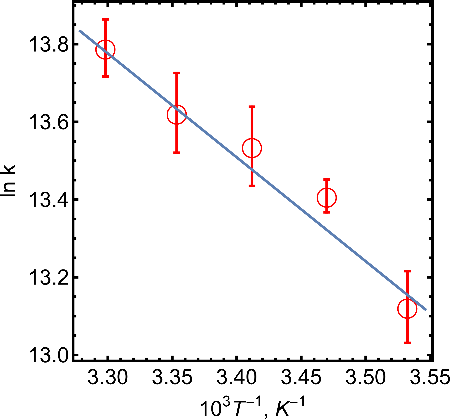


Figure S12. Kinetic curves of PZ oxidation (25 °C) (*left*) and the logarithmic correlation of apparent reaction rate constants with temperature for CIP (*middle*) and HRP (*right*).

From the experimental results, the free activation energies were calculated as $\text{2.2±0.8}\text{ }\text{kcal}\text{/}\text{mol}\text{ and }\text{5±1}\text{ }\text{kcal}\text{/}\text{mol}\text{,}$ and the pre-exponential factors were found to be$\text{1.3±4.7×}\text{10}^{\text{8}}\text{M}^{\text{-1}}\text{s}^{\text{-1}}$ and $\text{7±26×}\text{10}^{\text{9}}\text{M}^{\text{-1}}\text{s}^{\text{-1}}$ for CIP and HRP, respectively.

CV experiments for determination of the PZ reduction potential were carried out by using a clean glassy carbon electrode at a PZ concentration of 1 mM. CV measurements were performed in 50 mM phosphate buffer solution at pH 7.00, 25 °C (Figure S13).


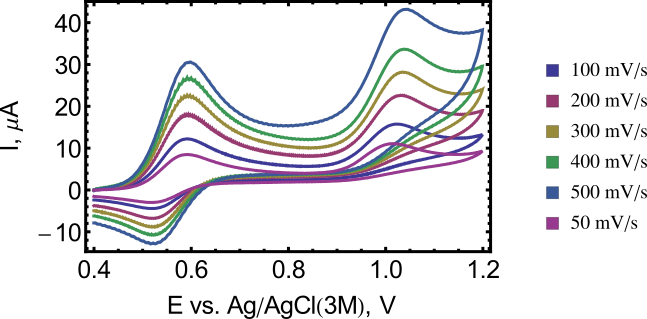


Figure S13. CVs of PZ on the glassy carbon working electrode (potential sweep rates are presented in the right panel).

The first-electron reduction potential was estimated to be $\text{0.557±0.006 V}\text{ }\text{vs. Ag}\text{/}\text{AgCl(3M)}$.

## 7.6. Activation energy of DMB oxidation and its reduction potential

The reaction scheme of DMB oxidation is postulated as:

$\begin{aligned} E_{\mathrm{red}}+H_{2}O_{2}\overset{k_{H_{2}O_{2}}}{\to}CpdI \\ CpdI+S\overset{k_{1}}{\to}CpdII+S^{\cdot+} \\ \begin{matrix} CpdII+S\overset{k_{2}}{\to}E_{\mathrm{red}}+S^{\cdot+} \end{matrix} \end{aligned}$ (S35)

$S^{\cdot+} + S^{\cdot+}\overset{k_{\mathrm{dis}}}{\to}S+S_{\mathrm{ox}}$

The limiting apparent bimolecular rate constants were calculated as $k_{\lim}=\frac{k_{1}k_{2}}{k_{1}+k_{2}}$. The initial concentrations of DMB varied from 2.3 to 16 µM for CIP, and the initial concentration of the enzyme was 31 nM. Experimental results are presented in Table S9 and Figure S14. The initial concentrations of DMB varied from 1.1 to 13.4 µM for HRP, and the initial concentration of the enzyme was 300 nM. Experimental results are presented in Table S9 and Figure S14.

Table S9. Apparent bimolecular rate constants of DMB oxidation measured at different temperatures.

| *t*, °C | Apparent bimolecular rate  constant (CIP), M^-1^s^-1^ | *t*, °C | Apparent bimolecular rate  constant (HRP), M^-1^s^-1^ |
| --- | --- | --- | --- |
| 12.9 | (1.25±0.03) × 10^6^ | 10 | (3.0±0.1) × 10^6^ |
| 15 | (1.43±0.05) × 10^6^ | 15 | (3.7±0.3) × 10^6^ |
| 20 | (1.8±0.2) × 10^6^ | 19.8 | (5.7±0.1) × 10^6^ |
| 25 | (2.3±0.1) × 10^6^ | 25 | (7.8±0.6) × 10^6^ |
| 30 | (2.8±0.3) × 10^6^ | 30 | (9.8±0.9) × 10^6^ |


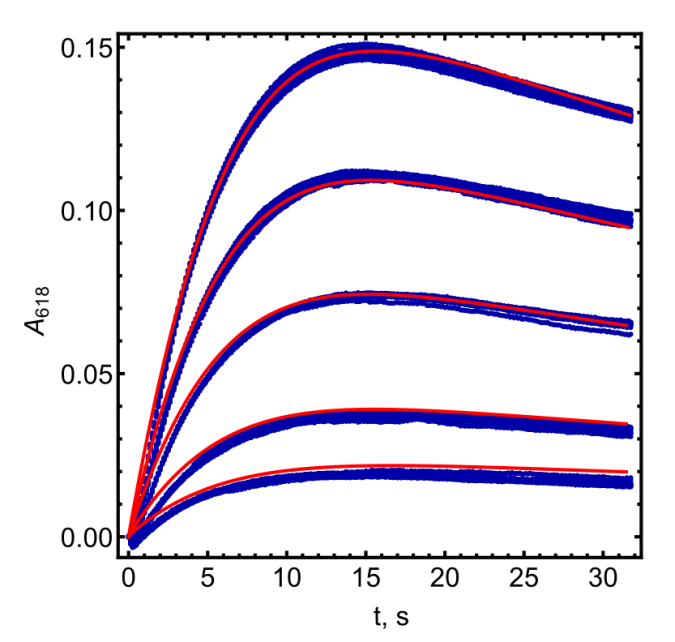

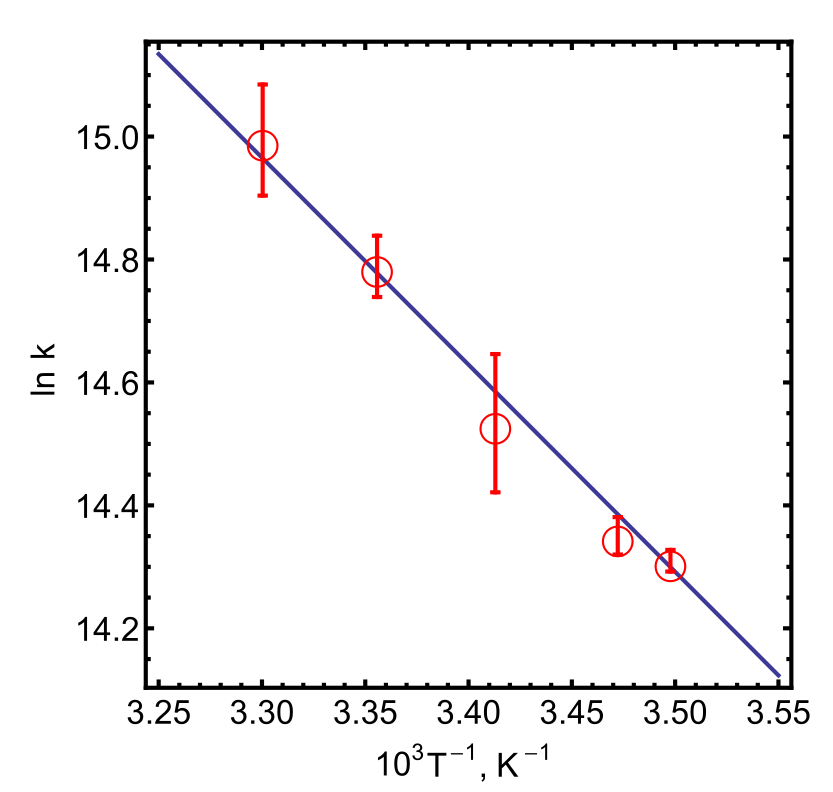

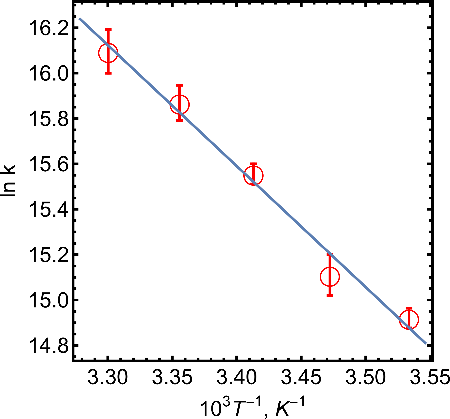


Figure S14. Kinetic curves of DMB oxidation (25 °C) (*left*) and the logarithmic correlation of apparent reaction rate constants with temperature for CIP (*middle*) and HRP (*right*).

From the experimental results, the free activation energies were calculated as $\text{8.0±1.2 kcal}\text{/}\text{mol}\text{ and }\text{10.6±0.8 kcal}\text{/}\text{mol}$, and the pre-exponential factors were found to be $\text{2.1±31×}\text{10}^{\text{11}}\text{M}^{\text{-1}}\text{s}^{\text{-1}}$ and $\text{6±10×}\text{10}^{\text{14}}\text{M}^{\text{-1}}\text{s}^{\text{-1}}$ for CIP and HRP, respectively.

Due to quite limited solubility of DMB in water, CV experiments for determination of the DMB reduction potential were done by mixing 10 µl of 4 mM DMB solution in acetonitrile with 10 µl of deionized water and dropping the mixture on a clean gold electrode. Then, the drop of the surface was then allowed to evaporate, with DMB remaining adsorbed on the electrode. CV measurements were performed in 50 mM phosphate buffer solution at pH 7.00, 25 °C (Figure S15).


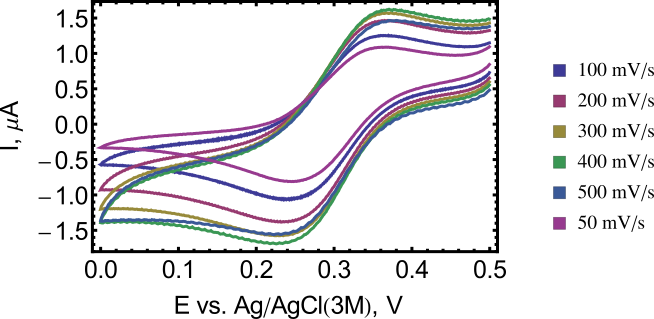


Figure S15. CVs of DMB adsorbed on the gold working electrode (potential sweep rates are presented in the right panel).

The first-electron reduction potential was estimated to be $0.301\pm0.004V vs. Ag\text{/}AgCl(3M)$.

## 7.7. Activation energy of TMPD oxidation, its reduction potential and kinetic isotope effect

The reaction scheme of TMPD oxidation is postulated as:

$\begin{aligned} E_{\mathrm{red}}+H_{2}O_{2}\overset{k_{H_{2}O_{2}}}{\to}CpdI \\ CpdI+S\overset{k_{1}}{\to}CpdII+S^{\cdot+} \\ \begin{matrix} CpdII+S\overset{k_{2}}{\to}E_{\mathrm{red}}+S^{\cdot+} \end{matrix} \end{aligned}$ (S36)

$S^{\cdot+} + S^{\cdot+}\overset{k_{\mathrm{dis}}}{\to}S+S_{\mathrm{ox}}$

The limiting apparent bimolecular rate constants were calculated as $k_{\lim}=\frac{k_{1}k_{2}}{k_{1}+k_{2}}$. The initial concentrations of TMPD varied from 4.2 to 34 µM for CIP, and the initial concentration of the enzyme was 15 nM. Experimental results are presented in Table S10 and Figure S16. The initial concentrations of TMPD varied from 3.9 to 57.7 µM for HRP, and the initial concentration of the enzyme was 100 nM. Experimental results are presented in Table S10 and Figure S16. The limiting rate constants of TMPD oxidation in D_2_O at 25 °C were measured using the H_2_O concentrations of enzyme and substrates in 50 mM phosphate buffer solution, pD 7.00.

Table S10. Apparent bimolecular rate constants of TMPD oxidation measured at different temperatures.

| *t*, °C | Apparent bimolecular rate  constant (CIP), M^-1^s^-1^ | *t*, °C | Apparent bimolecular rate  constant (HRP), M^-1^s^-1^ |
| --- | --- | --- | --- |
| 12.8 | (8.5±0.3) × 10^6^ | 10 | (1.80±0.03) × 10^7^ |
| 15 | (8.8±0.06) × 10^6^ | 15 | (2.30±0.08) × 10^7^ |
| 20 | (1.3±0.1) × 10^7^ | 19.8 | (3.5±0.1) × 10^7^ |
| 25 | (1.76±0.01) × 10^7^ | 25 | (4.6±0.2) × 10^7^ |
| 30 | (2.4±0.1) × 10^7^ | 30 | (5.3±0.2) × 10^7^ |


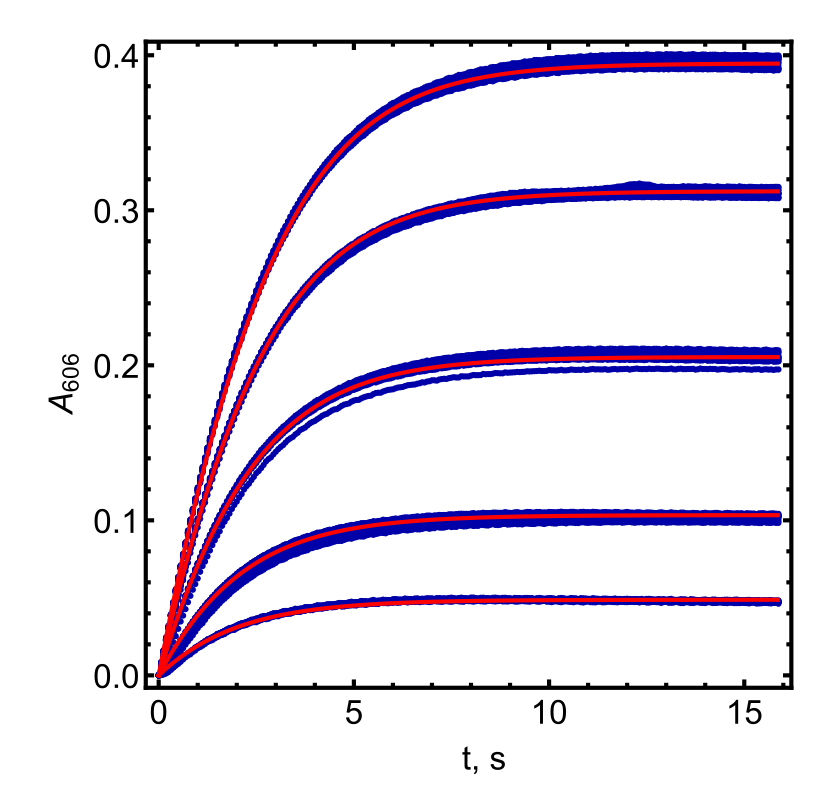

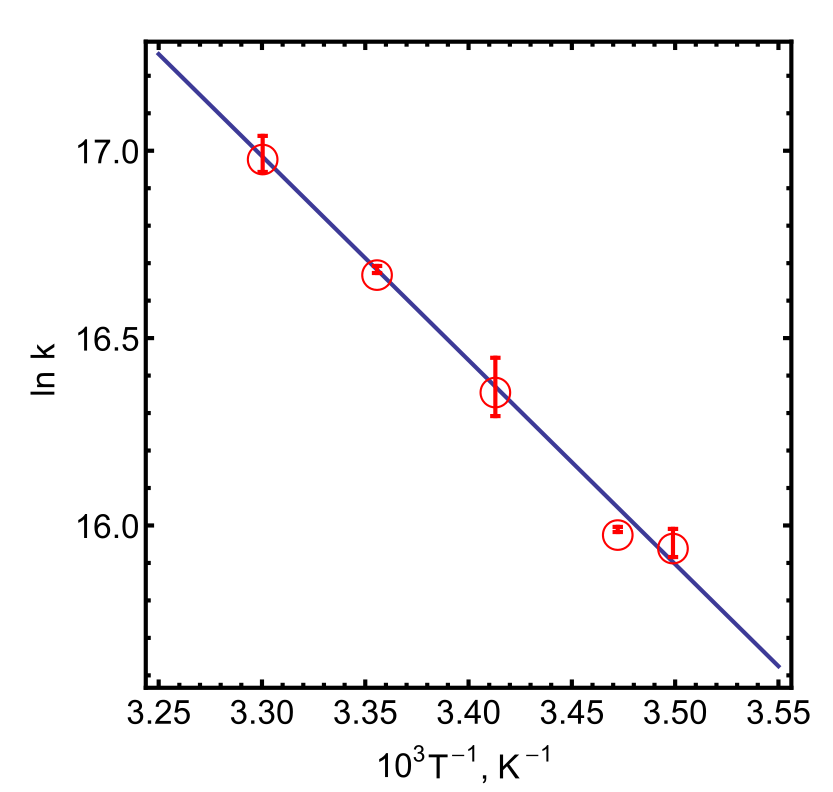

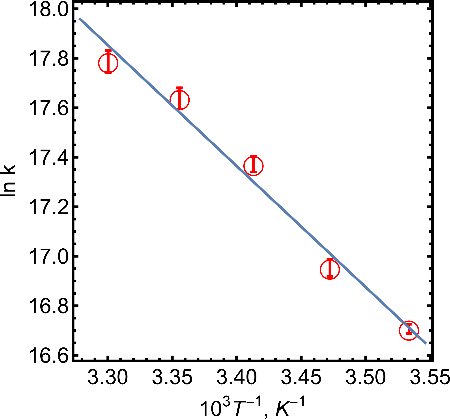


Figure S16. Kinetic curves of TMPD oxidation (25 °C) (*left*) and the logarithmic correlation of apparent reaction rate constants with temperature for CIP (*middle*) and HRP (*right*).

From the experimental results, the free activation energies were calculated as $\text{11.6±2.0 kcal}\text{/}\text{mol}\text{ and }\text{9.7±0.8 kcal}\text{/}\text{mol}$, and the pre-exponential factors were found to be $\text{1.5±14×}\text{10}^{\text{15}}\text{M}^{\text{-1}}\text{s}^{\text{-1}}$ and $\text{6±10×}\text{10}^{\text{14}}\text{M}^{\text{-1}}\text{s}^{\text{-1}}$ for CIP and HRP, respectively. The calculated kinetic isotope effect for TMPD oxidation was about 1.6±0.2. The rate constants were calculated from data taking into account the kinetic isotope effect of CpdI formation as well. The calculated kinetic isotope effect for TMPD oxidation with HRP was 1.9±0.3.

CV experiments for determination of the TMPD reduction potential were carried out by using a clean gold electrode at a TMPD concentration of 1 mM. CV measurements were performed in 50 mM phosphate buffer solution at pH 7.00, 25 °C (Figure S17).


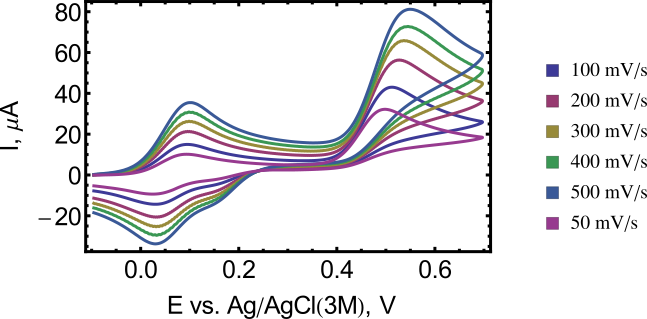


Figure S17. CVs of TMPD on the gold working electrode (potential sweep rates are presented in the right panel).

The first-electron reduction potential was estimated to be $\text{0.060±0.006 V vs. Ag}\text{/}\text{AgCl(3M)}$.

## 7.8. Activation energy of PPSA oxidation and its reduction potential

The reaction scheme of PPSA oxidation is postulated as:

$\begin{aligned} E_{\mathrm{red}}+H_{2}O_{2}\overset{k_{H_{2}O_{2}}}{\to}CpdI \\ CpdI+S\underset{k_{-1}}{\overset{k_{1}}{\rightleftharpoons}}CpdI\cdot S \\ CpdI\cdot S\overset{k_{\mathrm{cat},1}}{\to}CpdII+S^{\cdot+} \\ CpdII+S\underset{k_{-2}}{\overset{k_{2}}{\rightleftharpoons}}CpdII\cdot S \\ \begin{matrix} CpdII\cdot S\overset{k_{\mathrm{cat},2}}{\to}E_{\mathrm{red}}+S^{\cdot+} \end{matrix} \end{aligned}$ (S37)

The apparent bimolecular rate constants were calculated as for the HEPX oxidation experiments, according to Equations (S30-S31). The initial concentrations of PPSA varied from 1.1 to 10 µM for CIP, and the initial concentration of the enzyme was 14.8 nM. Experimental results are presented in Table S11 and Figure S18. The initial concentrations of PPSA varied from 1.5 to 10 µM for HRP, and the initial concentration of the enzyme was 62 nM. Experimental results are presented in Table S11 and Figure S18.

Table S11. Apparent bimolecular rate constants of PPSA oxidation measured at different temperatures.

| *t*, °C | Apparent bimolecular rate  constant (CIP), M^-1^s^-1^ | *t*, °C |  | Apparent bimolecular rate  constant (HRP), M^-1^s^-1^ |
| --- | --- | --- | --- | --- |
| 10 | (1.49±0.05) × 10^8^ | 10.1 |  | (2.60±0.03) × 10^7^ |
| 15 | (1.67±0.06) × 10^8^ | 15.1 |  | (3.0±0.1) × 10^7^ |
| 20 | (2.07±0.04) × 10^8^ | 20 |  | (4.06±0.05) × 10^7^ |
| 25 | (2.37±0.03) × 10^8^ | 25.1 |  | (4.82±0.04) × 10^7^ |
| 30 | (2.78±0.06) × 10^8^ | 30.2 |  | (5.12±0.07) × 10^7^ |


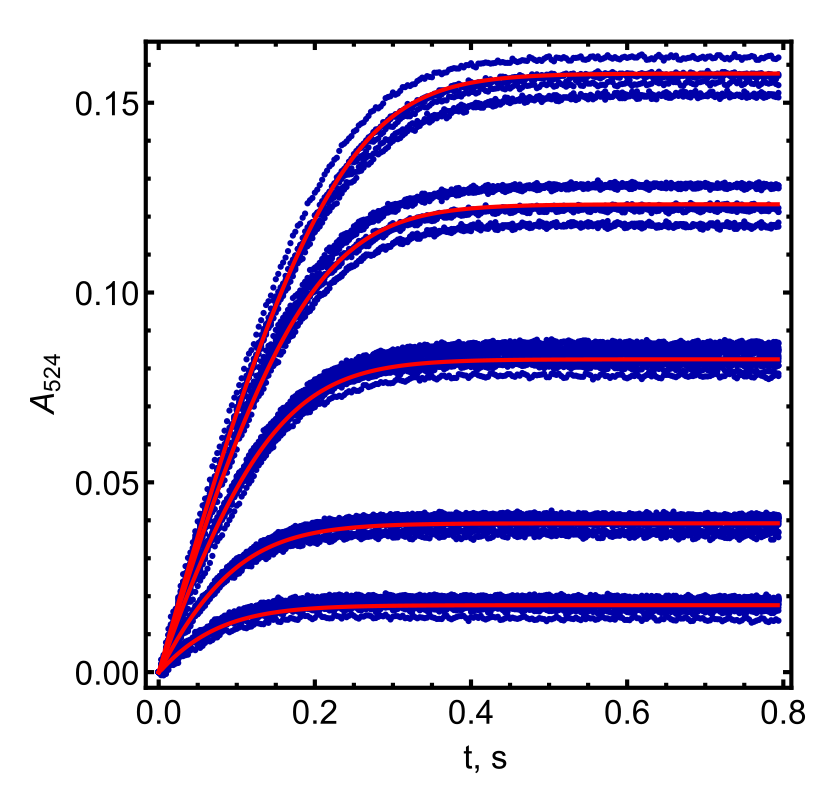

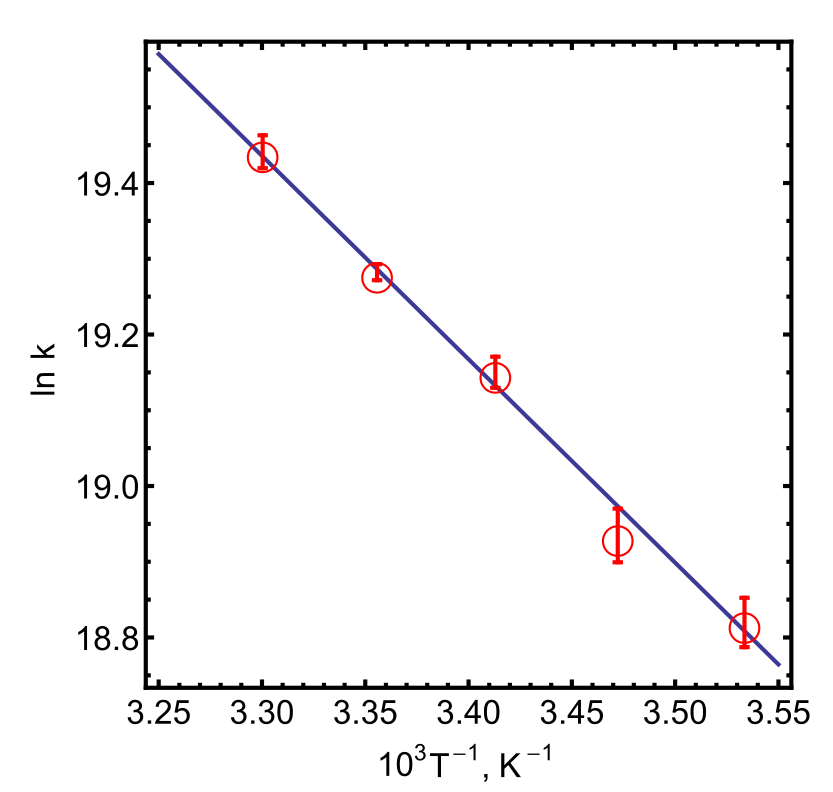

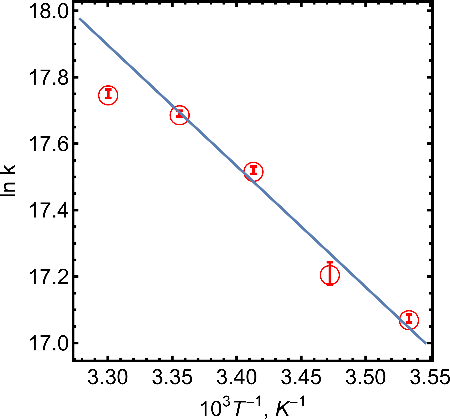


Figure S18. Kinetic curves of PPSA oxidation (25 °C) (*left*) and the logarithmic correlation of apparent reaction rate constants with temperature for CIP (*middle*) and HRP (*right*).

From the experimental results, the free activation energies were calculated as 5.3±0.8 kcal⁄mol and 7.2±0.8 kcal⁄mol, and the pre-exponential factors were found to be 2±5 × 10^12^ M^-1^s^-1^ for CIP and HRP, respectively.

CV experiments for determination of the PPSA reduction potential were carried out using a clean glassy carbon electrode at a PPSA concentration of 1 mM. CV measurements were performed in 50 mM phosphate buffer solution at pH 7.00, 25 °C (Figure S19).


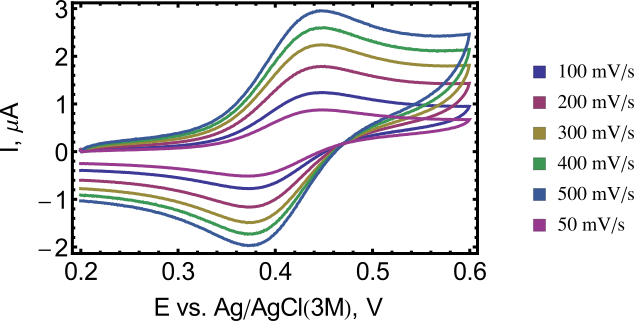


Figure S19. CVs of PPSA on the glassy carbon working electrode (potential sweep rates are presented in the right panel).

The first-electron reduction potential was estimated to be $\text{0.413±0.003 V vs. Ag}\text{/}\text{AgCl(3M)}$.

## 7.9. Activation energy of CPZ oxidation, its reduction potential and kinetic isotope effect

The reaction scheme of CPZ oxidation is postulated as:

$\begin{aligned} E_{\mathrm{red}}+H_{2}O_{2}\overset{k_{H_{2}O_{2}}}{\to}CpdI \\ CpdI+S\underset{k_{-1}}{\overset{k_{1}}{\rightleftharpoons}}CpdI\cdot S \\ CpdI\cdot S\overset{k_{\mathrm{cat},1}}{\to}CpdII+S^{\cdot+} \\ CpdII+S\underset{k_{-2}}{\overset{k_{2}}{\rightleftharpoons}}CpdII\cdot S \\ \begin{matrix} CpdII\cdot S\overset{k_{\mathrm{cat},2}}{\to}E_{\mathrm{red}}+S^{\cdot+} \end{matrix} \end{aligned}$ (S38)

$S^{\cdot+} + S^{\cdot+}\overset{k_{\mathrm{dis}}}{\to}S+S_{\mathrm{ox}}$

The rate constants were calculated as for the HEPX oxidation experiments, according to Equations (S30-S31). The initial concentrations of CPZ varied from 3.0 to 24 µM for CIP, and the initial concentration of the enzyme was 26 nM. The initial concentrations of CPZ were varied from 5.4 to 42.9 µM for HRP, and the initial concentration of the enzyme was 893 nM. Experimental results are presented in Table S12 and Figure S20. The limiting rate constant of CPZ oxidation in D_2_O at 25 °C were measured using the H_2_O concentrations of enzyme and substrates in 50 mM phosphate buffer solution, pD 7.00.

Table S12. Apparent bimolecular rate constants of CPZ oxidation measured at different temperatures.

| *t*, °C |  | Apparent bimolecular rate  constant (CIP), M^-1^s^-1^ | *t*, °C | Apparent bimolecular rate  constant (HRP), M^-1^s^-1^ |
| --- | --- | --- | --- | --- |
| 10 |  | (3.1±0.2) × 10^6^ | 10 | (6.5±0.5) × 10^4^ |
| 15 |  | (3.0±0.1) × 10^6^ | 15 | (7±2) × 10^4^ |
| 20 |  | (2.9±0.2) × 10^6^ | 20 | (8.7±0.5) × 10^4^ |
| 25 |  | (3.1±0.2) × 10^6^ | 24.8 | (1.22±0.08) × 10^5^ |
| 30 |  | (3.0±0.2) × 10^6^ | 30 | (1.2±0.3) × 10^5^ |


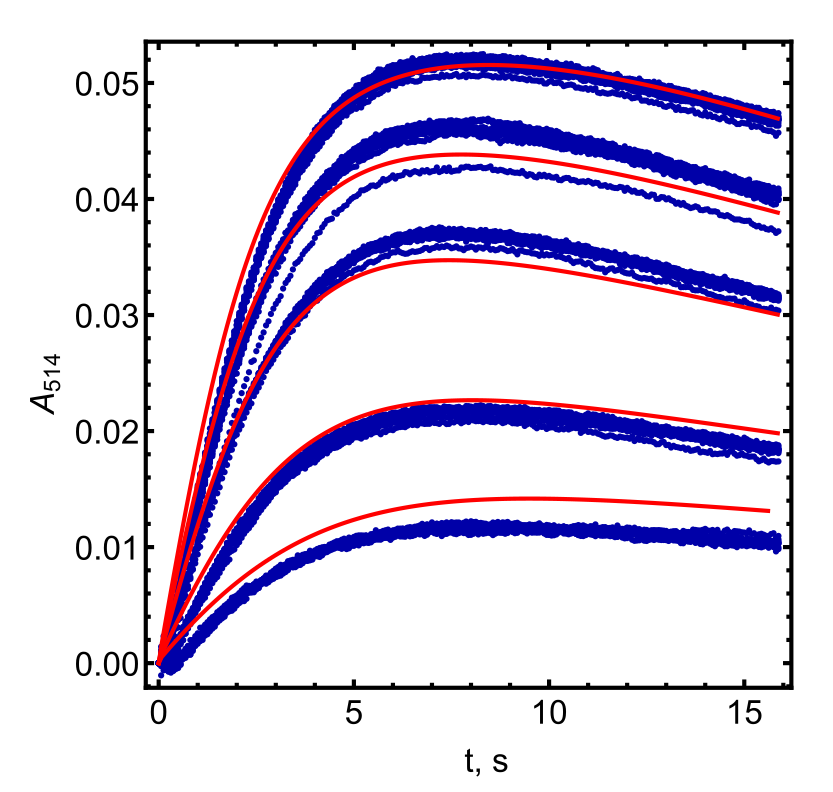

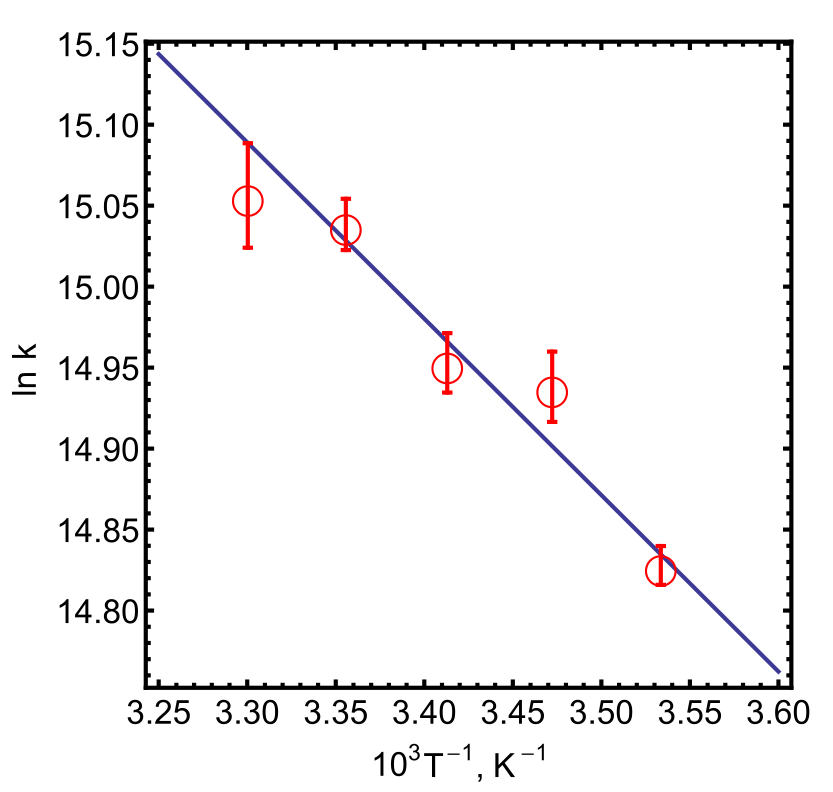

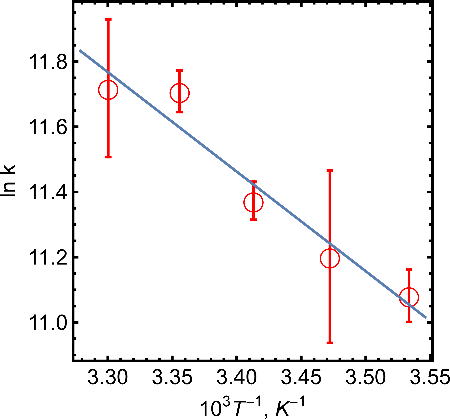


Figure S20. Kinetic curves of CPZ oxidation (25 °C) (*left*) and the logarithmic correlation of apparent reaction rate constants with temperature for CIP (*middle*) and HRP (*right*).

From the experimental results, the free activation energies were calculated as $\text{0±1 kcal}\text{/}\text{mol}\text{ and }\text{6.1±0.7 kcal}\text{/}\text{mol}$, and as the pre-exponential factors were found to be $\text{2±10×}\text{10}^{\text{6}}{\text{ }\text{M}}^{\text{-1}}\text{s}^{\text{-1}}$ and $\text{4±23×}\text{10}^{\text{9}}{\text{ }\text{M}}^{\text{-1}}\text{s}^{\text{-1}}$ for CIP and HRP, respectively. The calculated kinetic isotope effect was 1.0±0.2 for both CIP and HRP. The rate constants were calculated from data taking into account the kinetic isotope effect of CpdI formation as well.

CV experiments for determination of the PZ reduction potential were carried out using a clean glassy carbon electrode at a CPZ concentration of 1 mM. CV measurements were performed in 50 mM phosphate buffer solution at pH 7.00, 25 °C (Figure S21).


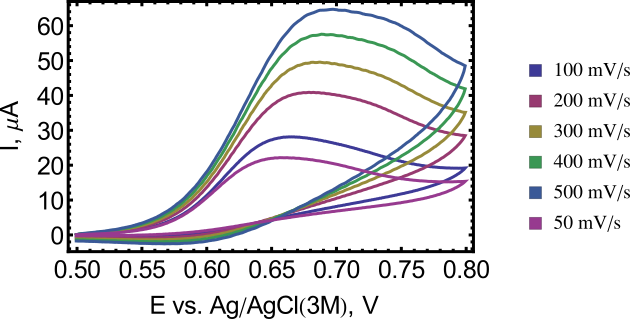


Figure S21. CVs of CPZ on the glassy carbon working electrode (potential sweep rates are presented in the right panel).

The first-electron reduction potential was estimated to be $\text{0.614±0.005 V vs. Ag}\text{/}\text{AgCl(3M)}$. This value was approximated by subtracting 30 mV from the oxidation peak potential.^11^

## 7.10. Activation energy of MB oxidation and its reduction potential

The reaction scheme of MB oxidation is postulated as:

$\begin{aligned} E_{\mathrm{red}}+H_{2}O_{2}\overset{k_{H_{2}O_{2}}}{\to}CpdI \\ CpdI+S\overset{k_{1}}{\to}CpdII+S^{\cdot+} \\ \begin{matrix} CpdII+S\overset{k_{2}}{\to}E_{\mathrm{red}}+S^{\cdot+} \end{matrix} \end{aligned}$ (S39)

$S^{\cdot+} + S^{\cdot+}\overset{k_{\mathrm{dis}}}{\to}S+S_{\mathrm{ox}}$

The limiting apparent bimolecular rate constants were calculated as $k_{\lim}=\frac{k_{1}k_{2}}{k_{1}+k_{2}}$. The initial concentrations of MB varied from 0.23 to 1.3 µM for CIP, and the initial concentration of the enzyme was 27 nM. Experimental results are presented in Table S13 and Figure S22. The initial concentrations of MB varied from 0.18 to 1.4 µM for HRP, and the initial concentration of the enzyme was 30.8 nM. Experimental results are presented in Table S13 and Figure S22.

Table S13. Apparent bimolecular rate constants of MB oxidation measured at different temperatures.

| *t*, °C | Apparent bimolecular rate  constant (CIP), M^-1^s^-1^ | *t*, °C | Apparent bimolecular rate  constant (HRP), M^-1^s^-1^ |
| --- | --- | --- | --- |
| 10 | (1.23±0.01) × 10^8^ | 10 | (1.01±0.03) × 10^8^ |
| 15 | (1.56±0.03) × 10^8^ | 15 | (1.20±0.06) × 10^8^ |
| 20 | (2.0±0.2) × 10^8^ | 20 | (1.70±0.05) × 10^8^ |
| 25 | (2.66±0.03) × 10^8^ | 25 | (2.01±0.04) × 10^8^ |
| 30 | (3.2±0.2) × 10^8^ | 30 | (2.40±0.06) × 10^8^ |


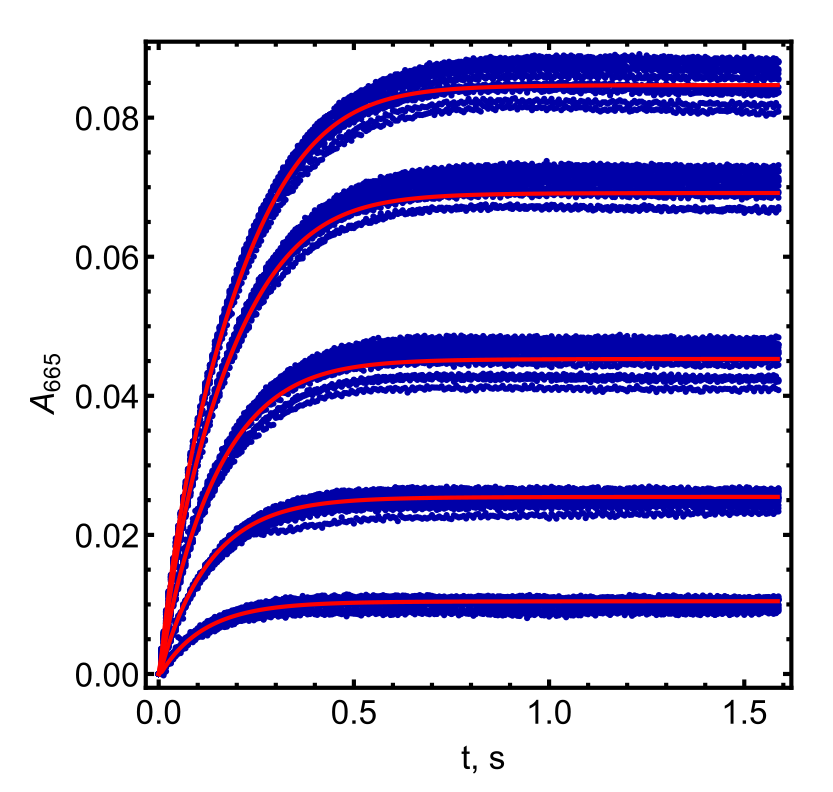

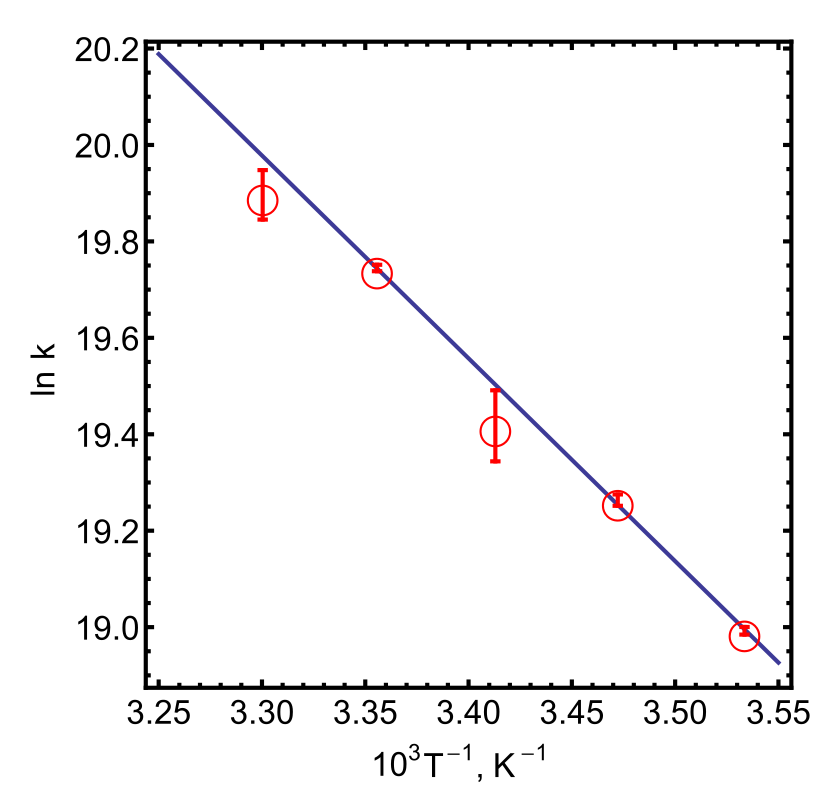

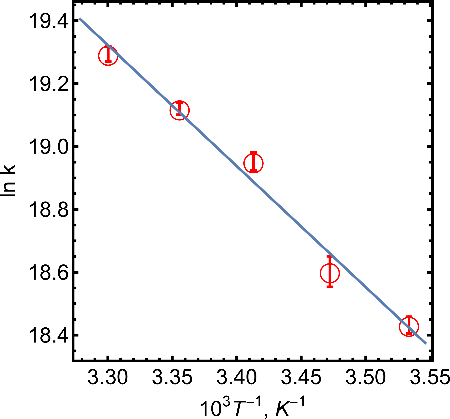


Figure S22. Kinetic curves of MB oxidation (25 °C) (*left*) and the logarithmic correlation of apparent reaction rate constants with temperature for CIP (*middle*) and HRP (*right*).

From the experimental results, the free activation energy is calculated as $\text{8.4±0.4 kcal}\text{/}\text{mol}$ and $\text{7.6±0.6 kcal}\text{/}\text{mol}$, and the pre-exponential factors were found to be $\text{5±6×}\text{10}^{\text{15}}\text{M}^{\text{-1}}\text{s}^{\text{-1}}$ and $\text{8±14×}\text{10}^{\text{13}}\text{M}^{\text{-1}}\text{s}^{\text{-1}}$for CIP and HRP, respectively.

CV experiments for determination of the MB reduction potential were carried out using a clean glassy carbon electrode at a MB concentration of 0.5 mM. CV measurements were performed in 50 mM phosphate buffer solution at pH 7.00, 25 °C (Figure S23).


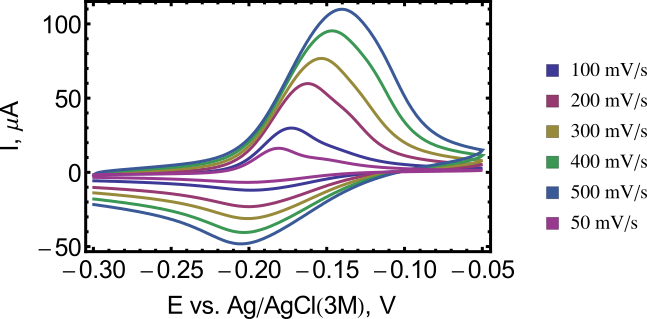


Figure S23. CVs of MB on the glassy carbon working electrode (potential sweep rates are presented in the right panel).

The two-electron reduction potential was estimated to be $\text{- 0.180±0.007 V vs. Ag}\text{/}\text{AgCl(3M)}$.

## 7.11. Activation energy of TH oxidation and its reduction potential

The reaction scheme of TH oxidation is postulated as:

$\begin{aligned} E_{\mathrm{red}}+H_{2}O_{2}\overset{k_{H_{2}O_{2}}}{\to}CpdI \\ CpdI+S\overset{k_{1}}{\to}CpdII+S^{\cdot+} \\ \begin{matrix} CpdII+S\overset{k_{2}}{\to}E_{\mathrm{red}}+S^{\cdot+} \end{matrix} \end{aligned}$ (S40)

$S^{\cdot+} + S^{\cdot+}\overset{k_{dis}}{\to}S+S_{\mathrm{ox}}$

The limiting apparent bimolecular rate constants were calculated as $k_{\lim}=\frac{k_{1}k_{2}}{k_{1}+k_{2}}$. The initial concentrations of TH varied from 0.097 to 0.7 µM for CIP, and the initial concentration of the enzyme was 29 nM. Experimental results are presented in Table S14 and Figure S24. The initial concentrations of TH varied from 0.09 to 0.68 µM for HRP, and the initial concentration of the enzyme was 41 nM. Experimental results are presented in Table S14 and Figure S24.

Table S14. Apparent bimolecular rate constants of TH oxidation measured at different temperatures.

| *t*, °C |  | Apparent bimolecular rate  constant (CIP), M^-1^s^-1^ | *t*, °C | Apparent bimolecular rate  constant (HRP), M^-1^s^-1^ |
| --- | --- | --- | --- | --- |
| 10 |  | (2.9±0.2) × 10^8^ | 10 | (1.60±0.04) × 10^8^ |
| 15 |  | (3.33±0.05) × 10^8^ | 15 | (1.84±0.09) × 10^8^ |
| 20 |  | (4.05±0.06) × 10^8^ | 20 | (2.30±0.09) × 10^8^ |
| 25 |  | (4.80±0.07) × 10^8^ | 25 | (2.80±0.05) × 10^8^ |
| 30 |  | (5.70±0.07) × 10^8^ | 30.3 | (3.4±0.1) × 10^8^ |


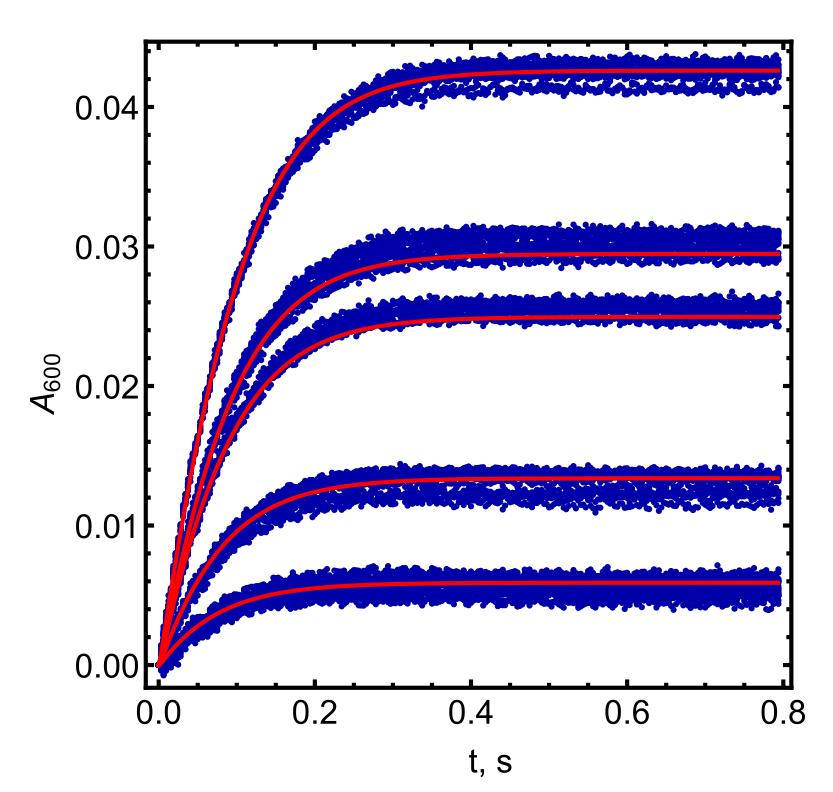

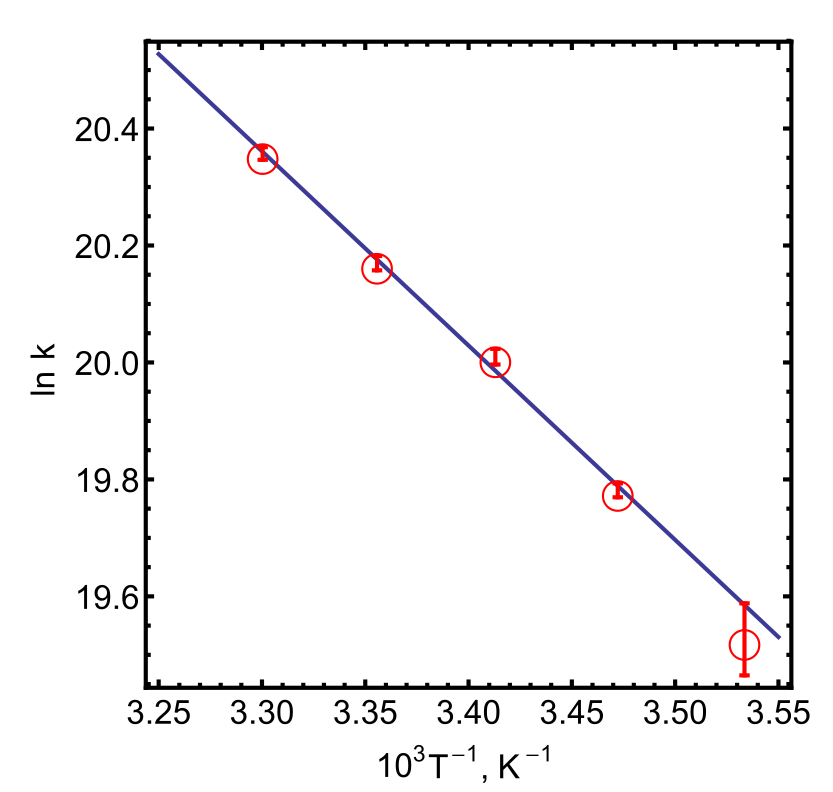

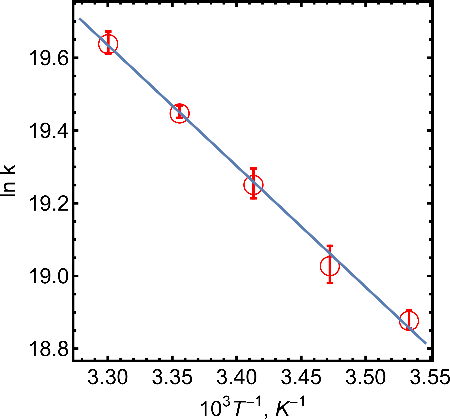


Figure S24. Kinetic curves of TH oxidation (25 °C) (*left*) and the logarithmic correlation of apparent reaction rate constants with temperature for CIP (*middle*) and HRP (*right*).

From the experimental results, the free activation energy is calculated as $\text{5.9±0.8 kcal}\text{/}\text{mol}$ and $\text{6.6±0.2 kcal}\text{/}\text{mol}$, and the pre-exponential factors were found to be $\text{4±9×}\text{10}^{\text{13}}\text{M}^{\text{-1}}\text{s}^{\text{-1}}$ and $\text{2±1×}\text{10}^{\text{13}}\text{M}^{\text{-1}}\text{s}^{\text{-1}}$for CIP and HRP, respectively.

CV experiments for determination of the TH reduction potential were carried out using a clean glassy carbon electrode at a TH concentration of 0.5 mM. CV measurements were performed in 50 mM phosphate buffer solution at pH 7.00, 25 °C (Figure S25).


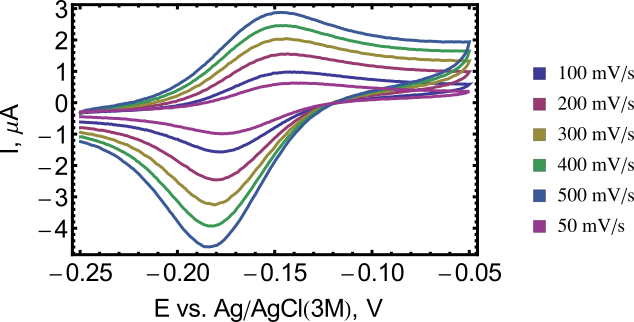


Figure S25. CVs of TH on the gold working electrode (potential sweep rates are presented in the right panel).

The two-electron reduction potential was estimated to be$\text{ 0.161±0.003 V vs. Ag}\text{/}\text{AgCl(3M)}$.

## 7.12. Activation energy of DCPIP oxidation and its reduction potential

The reaction scheme of DCPIP oxidation is postulated as:

$\begin{aligned} E_{\mathrm{red}}+H_{2}O_{2}\overset{k_{H_{2}O_{2}}}{\to}CpdI \\ CpdI+S\overset{k_{1}}{\to}CpdII+S^{\cdot+} \\ \begin{matrix} CpdII+S\overset{k_{2}}{\to}E_{\mathrm{red}}+S^{\cdot+} \end{matrix} \end{aligned}$

$S^{\cdot+} + S^{\cdot+}\overset{k_{\mathrm{dis}}}{\to}S+S_{\mathrm{ox}}$ (S41)

$\begin{aligned} CpdI+S_{\mathrm{ox}}\overset{k_{3}}{\to}CpdII+{S_{ox}}^{\cdot+} \\ \begin{matrix} CpdII+S_{\mathrm{ox}}\overset{k_{4}}{\to}E_{\mathrm{red}}+{S_{ox}}^{\cdot+} \end{matrix} \end{aligned}$

The limiting apparent bimolecular rate constants were calculated as $k_{\lim,1}=\frac{k_{1}k_{2}}{k_{1}+k_{2}}$ and $k_{\lim,2}=\frac{k_{3}k_{4}}{k_{3}+k_{4}}$. The initial concentrations of DCPIP varied from 0.37 to 3 µM for CIP, and the initial concentration of the enzyme was 29 nM. Experimental results are presented in Table S15 and Figure S26. The initial concentrations of DCPIP varied from 0.3 to 4.3 µM for HRP, and the initial concentration of HRP was 79 nM. Experimental results are presented in Table S15 and Figure S26.

Table S15. Apparent bimolecular rate constants of TMPD oxidation measured at different temperatures.

| *t*, °C | *CIP* | | *t*, °C | *HRP* | |
| --- | --- | --- | --- | --- | --- |
|  | $k_{\lim,1}$, M^-1^s^-1^ | $k_{\lim,2},$M^-1^s^-1^ |  | $k_{\lim,1}$, M^-1^s^-1^ | $k_{\lim,2},$M^-1^s^-1^ |
| 10 | (1.7±0.1) × 10^8^ | (8.8±0.5) × 10^6^ | 10 | (1.07±0.02) × 10^8^ | (1.28±0.02) × 10^6^ |
| 15.2 | (2.4±0.3) × 10^8^ | (9.7±0.6) × 10^6^ | 14.9 | (1.4±0.2) × 10^8^ | (1.41±0.04) × 10^6^ |
| 20 | (3.0±0.6) × 10^8^ | (1.1±0.1) × 10^7^ | 19.9 | (1.9±0.5) × 10^8^ | (1.68±0.07) × 10^6^ |
| 25 | (3.8±0.8) × 10^7^ | (1.3±0.2) × 10^7^ | 25 | (2.1±0.3) × 10^8^ | (1.93±0.05) × 10^6^ |
| 30 | (4.2±0.2) × 10^7^ | (1.30±0.04) × 10^7^ | 30 | (2.8±0.2) × 10^8^ | (1.98±0.09) × 10^6^ |


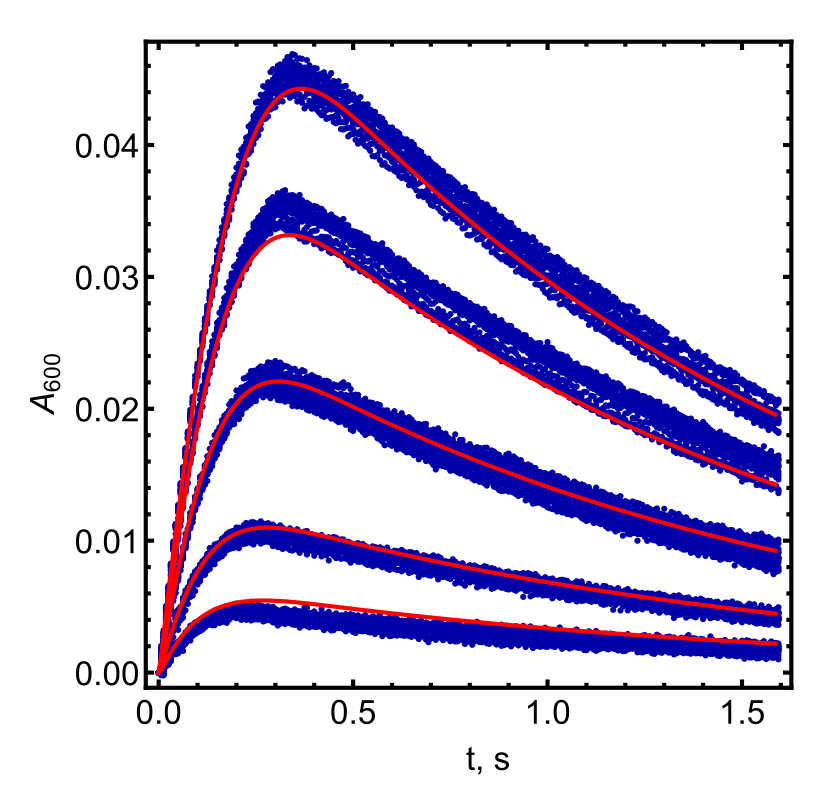

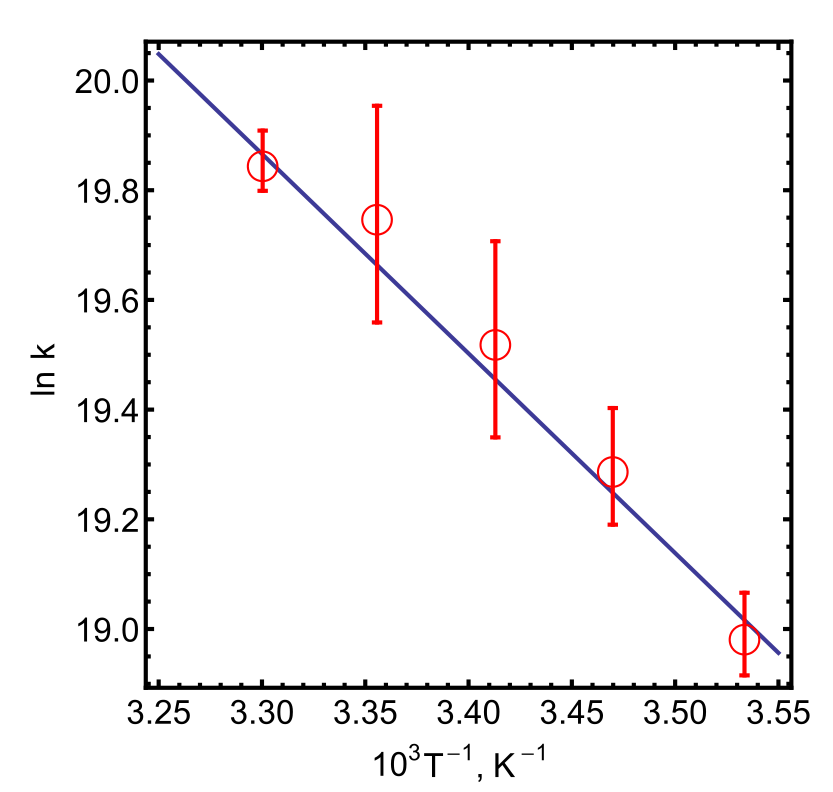

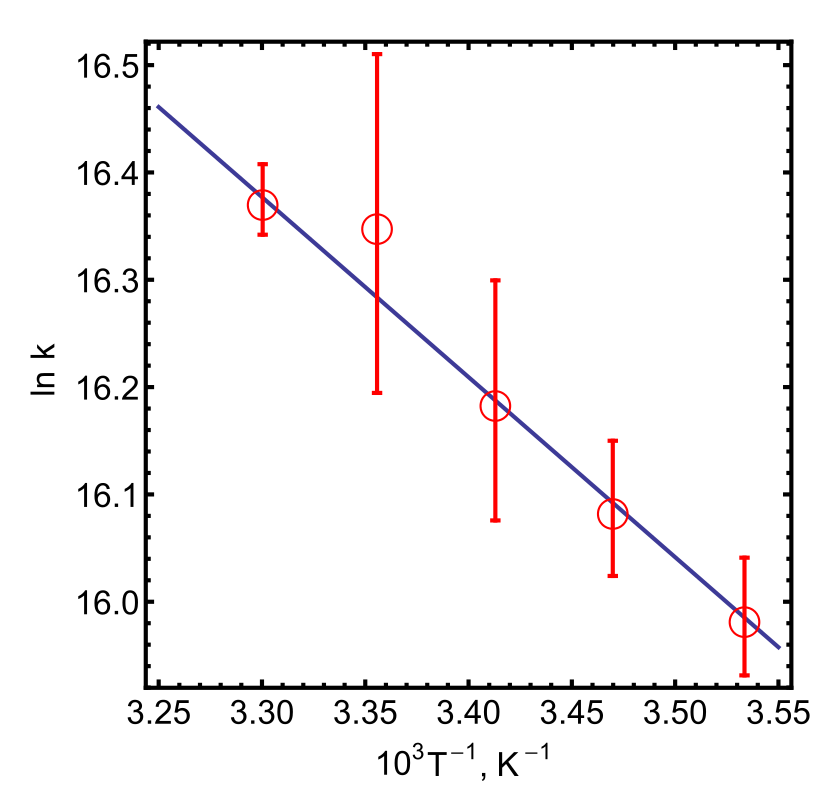

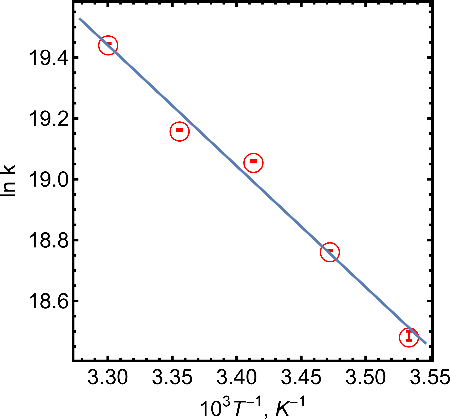

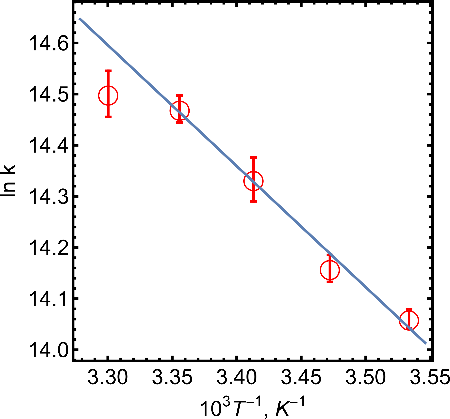


Figure S26. Kinetic curves of DCPIP oxidation (25 °C) (*top*) and the logarithmic correlation of apparent reaction rate constants $k_{\lim,1}$(*left*) and $k_{\lim,2}$ (*right*) with temperature for CIP (*top graphs*) and for HRP (*bottom graphs*).

In the case of CIP, from the experimental results, the free activation energy for fully reduced CpdII was calculated to be $\text{7.4±1.2kcal}\text{/}\text{mol}$, and the pre-exponential factor was found to be $\text{7±53×}\text{10}^{\text{13}}\text{M}^{\text{-1}}\text{s}^{\text{-1}}$. For fully oxidized CpdII, the estimated free activation energy was calculated to be $\text{3.3±0.3 kcal}\text{/}\text{mol}$, and the pre-exponential factor was found to be $\text{3±6×}\text{10}^{\text{9}}\text{M}^{\text{-1}}\text{s}^{\text{-1}}$. In the case of HRP, from the experimental results, the free activation energy for fully reduced CpdII was calculated to be $\text{7.9±0.6 kcal}\text{/}\text{mol}$, and the pre-exponential factor was found to be $\text{2±4×}\text{10}^{\text{14}}\text{M}^{\text{-1}}\text{s}^{\text{-1}}$. For fully oxidized CpdII, the estimated free activation energy was calculated to be $\text{4.7±0.9 kcal}\text{/}\text{mol}$, and the pre-exponential factor was found to be $\text{5±3×}\text{10}^{\text{9}}\text{M}^{\text{-1}}\text{s}^{\text{-1}}$.

CV experiments for determination of the DCPIP reduction potentials were carried out by using a clean glassy carbon electrode at a DCPIP concentration of 1 mM. CV measurements were performed in 50 mM phosphate buffer solution at pH 7.00, 25 °C (Figure S27).


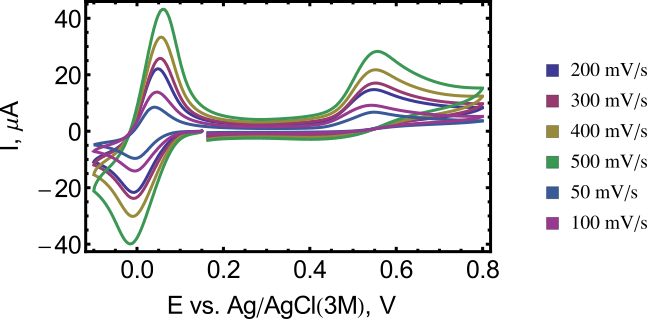


Figure S27. CVs of DCPIP on the glassy carbon working electrode (potential sweep rates are presented in the right panel).

The reduction potential of the first redox couple (two-electron process) was estimated to be $\text{0.021±0.003 V vs. Ag}\text{/}\text{AgCl(3M)}$. The reduction potential of the second redox couple (one-electron process) was estimated to be $\text{0.526±0.003V vs. Ag}\text{/}\text{AgCl(3M)}$ (the value was estimated by subtracting 30 mV from oxidation peak potential).^11^

## 7.13. Activation energy of VB oxidation and its reduction potential

The reaction scheme of VB oxidation is postulated as:

$\begin{aligned} E_{\mathrm{red}}+H_{2}O_{2}\overset{k_{H_{2}O_{2}}}{\to}CpdI \\ CpdI+S\overset{k_{1}}{\to}CpdII+S^{\cdot+} \\ \begin{matrix} CpdII+S\overset{k_{2}}{\to}E_{\mathrm{red}}+S^{\cdot+} \end{matrix} \end{aligned}$ (S42)

$S^{\cdot+} + S^{\cdot+}\overset{k_{\mathrm{dis}}}{\to}S+S_{\mathrm{ox}}$

The limiting apparent bimolecular rate constants were calculated as $k_{\lim}=\frac{k_{1}k_{2}}{k_{1}+k_{2}}$. The initial concentrations of VB varied from 4.5 to 30 µM for CIP, and the initial concentration of the enzyme was 26 nM. Experimental results are presented in Table S16 and Figure S28. The initial concentrations of VB varied from 1.4 to 9.3 µM for HRP, and the initial concentration of the enzyme was 59.5 nM. Experimental results are presented in Table S16 and Figure S28.

Table S16. Apparent bimolecular rate constants of VB oxidation measured at different temperatures.

| *t*, °C |  | Apparent bimolecular rate  constant (CIP), M^-1^s^-1^ | *t*, °C | Apparent bimolecular rate  constant (HRP), M^-1^s^-1^ |
| --- | --- | --- | --- | --- |
| 10 |  | (1.31±0.01) × 10^7^ | 9.9 | (6.60±0.06) × 10^7^ |
| 15 |  | (1.59±0.02) × 10^7^ | 15 | (8.7±0.2) × 10^7^ |
| 20 |  | (1.99±0.02) × 10^7^ | 19.9 | (1.04±0.02) × 10^8^ |
| 25 |  | (2.56±0.03) × 10^7^ | 25.1 | (1.23±0.05) × 10^8^ |
|  |  |  | 30.2 | (1.37±0.07) × 10^8^ |


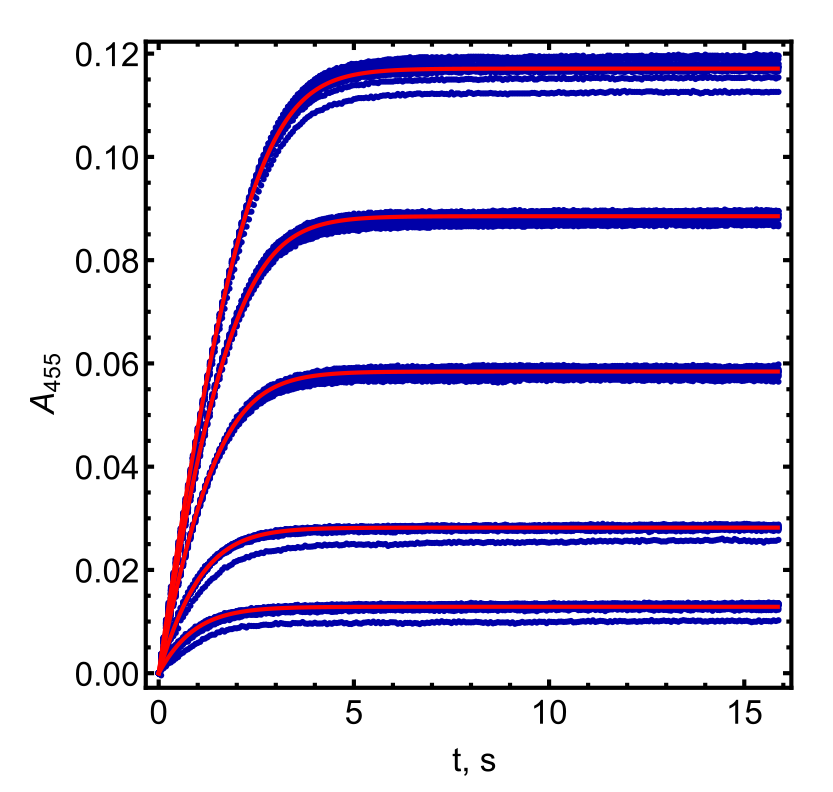

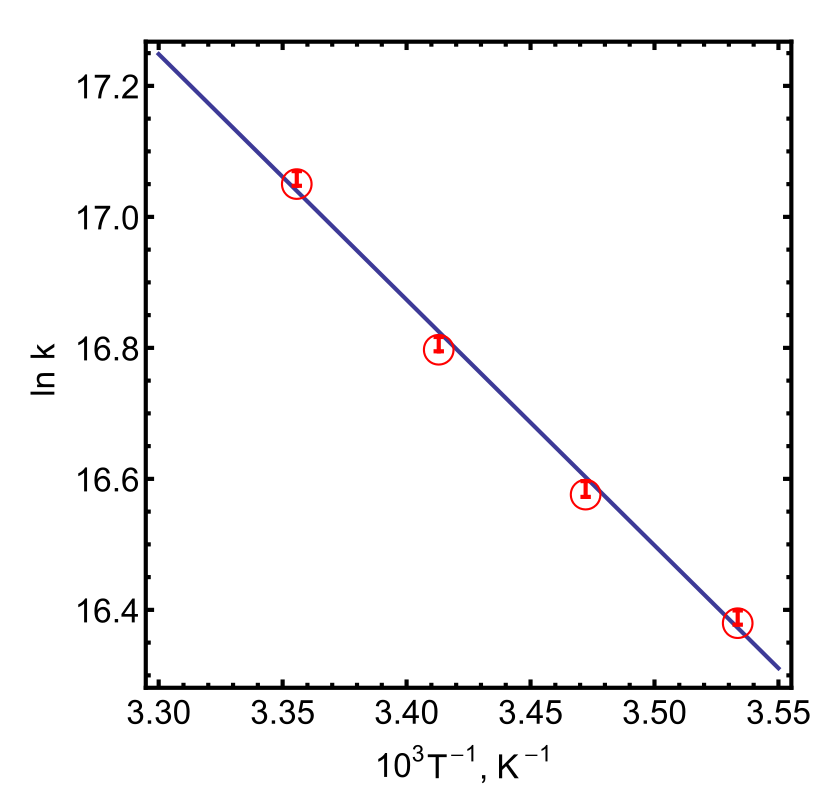

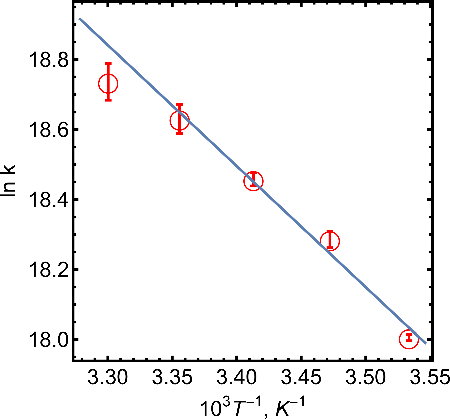


Figure S28. Kinetic curves of VB oxidation (t – 25 °C) (*left*) and the logarithmic correlation of apparent reaction rate constants with temperature for CIP (*middle*) and HRP (*right*).

From the experimental results, the free activation energies were calculated to be $\text{7.2±1.6 kcal}\text{/}\text{mol}$ $\text{6.9±0.5 kcal}\text{/}\text{mol}$, and the pre-exponential factors were found to be $\text{7±50×}\text{10}^{\text{12}}\text{ M}^{\text{-1}}\text{s}^{\text{-1}}$ and $\text{1±2×}\text{10}^{\text{13}}\text{ M}^{\text{-1}}\text{s}^{\text{-1}}$ for CIP and HRP, respectively.

CV experiments for determination of the VB reduction potential were carried out by using a clean glassy carbon electrode at a VB concentration of 1 mM. CV measurements were performed in 50 mM phosphate buffer solution at pH 7.00, 25 °C (Figure S29).


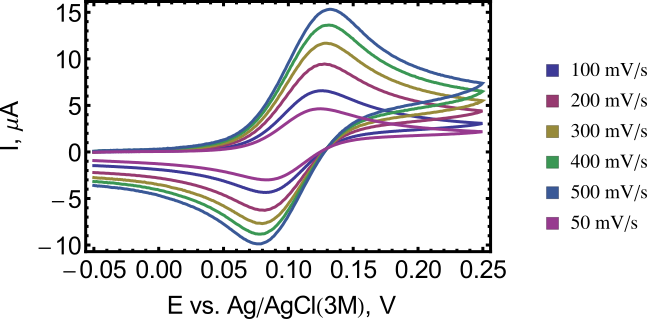


Figure S29. CVs of VB on the glassy carbon working electrode (potential sweep rates are presented in the right panel).

The two-electron reduction potential was estimated to be $\text{0.105±0.001 V vs. Ag}\text{/}\text{AgCl(3M)}$.

**8. Quantum computations**

Table S17. Optimized structures of the compounds.

| **Compound** | **Reduced** | **Oxidized** |
| --- | --- | --- |
| ABTS | 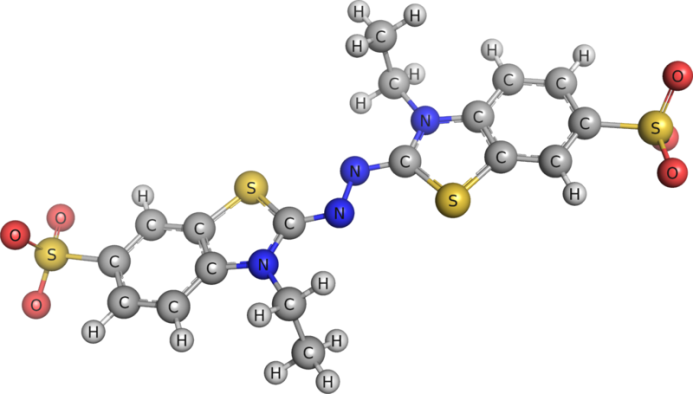  Di-anion | 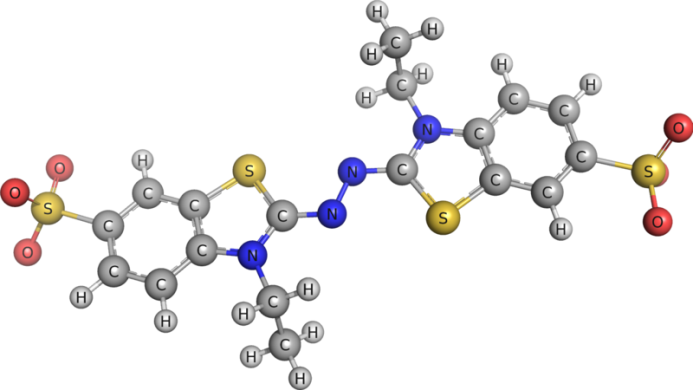  Radical anion |
| AMB | 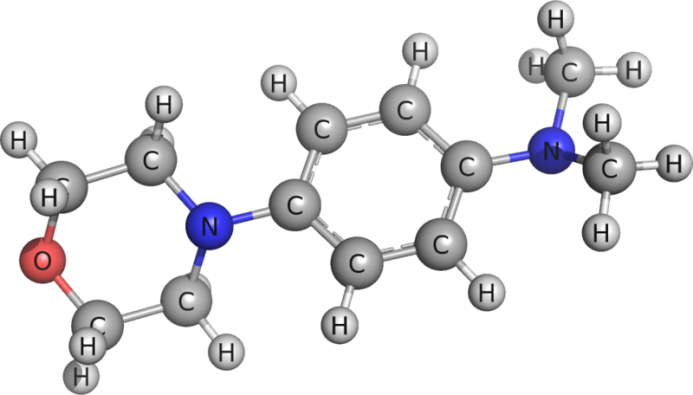  Neutral molecule | 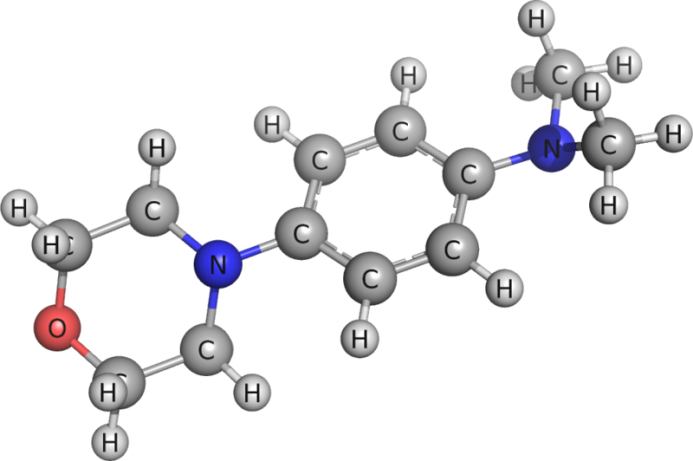  Radical cation |
| CPZ | 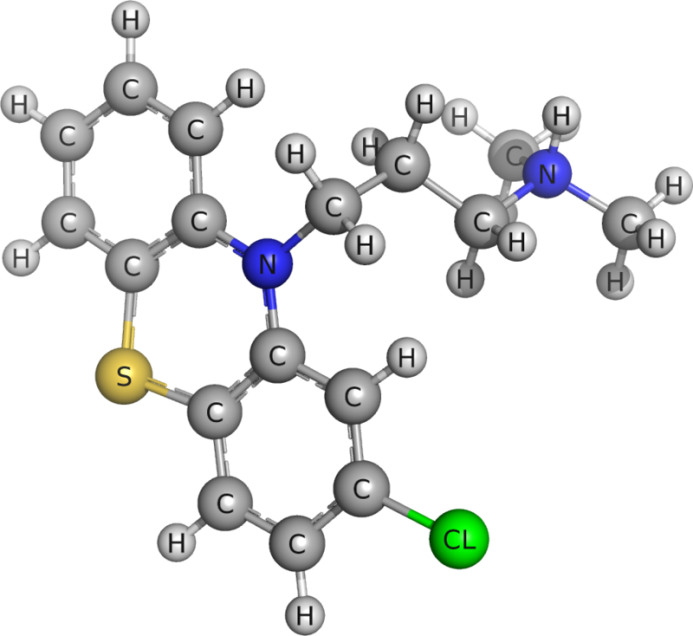  Cation | 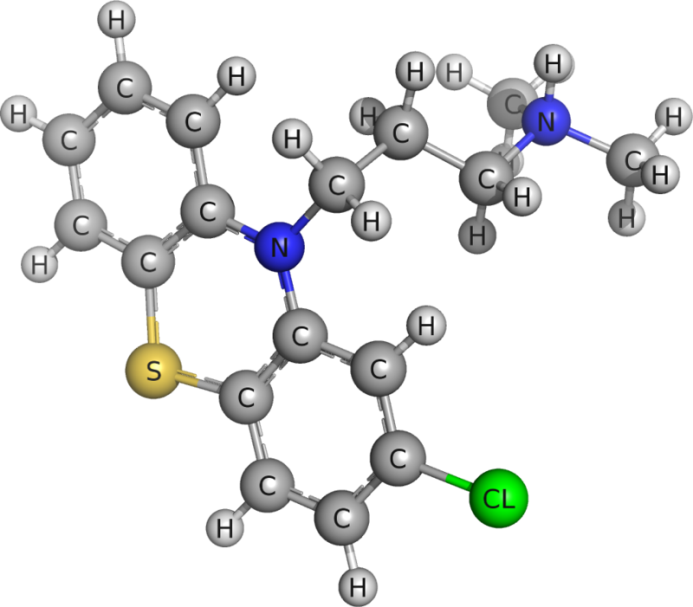  Radical di-cation |
| DCPIP(I)  second electron reduction | 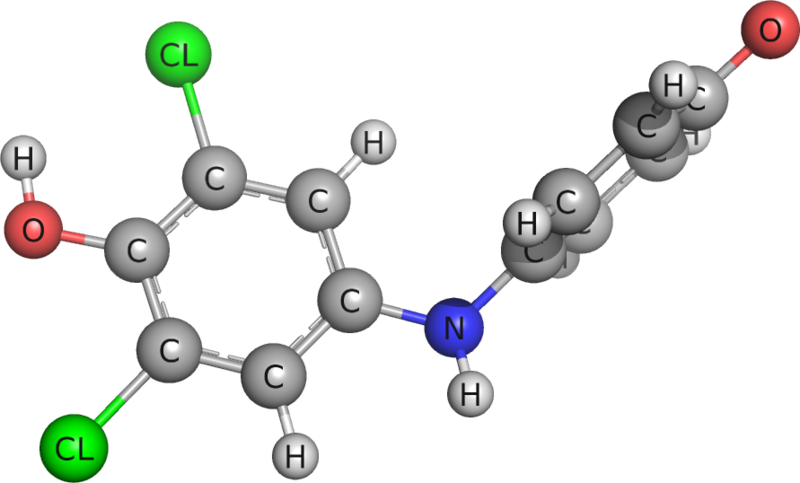  Anion | 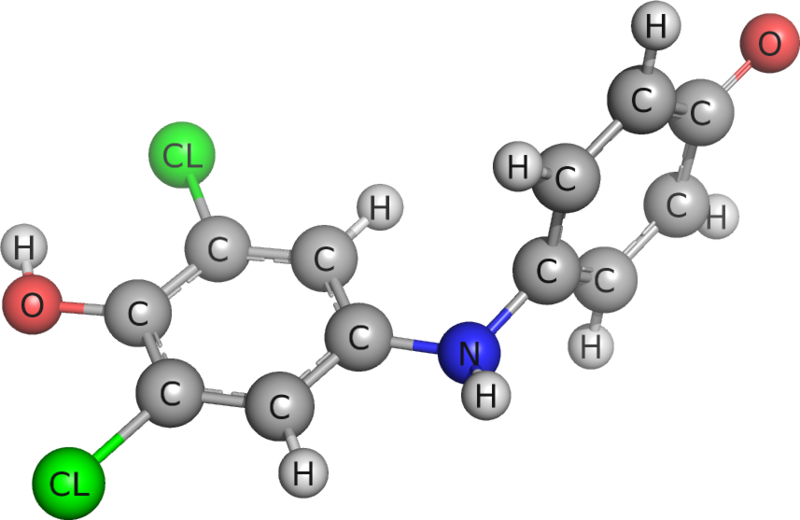  Radical |
| first electron reduction | 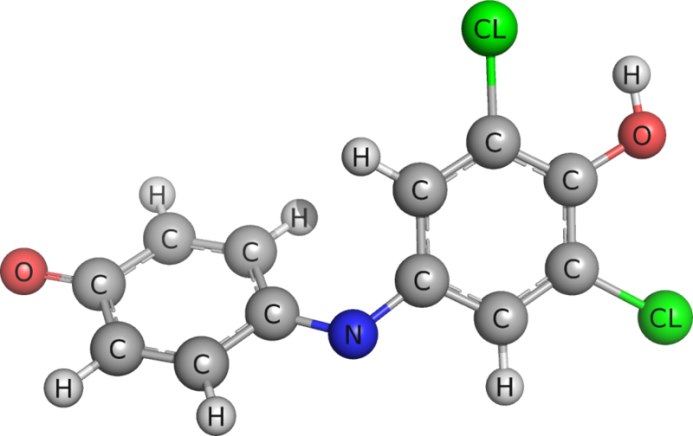  Radical anion | 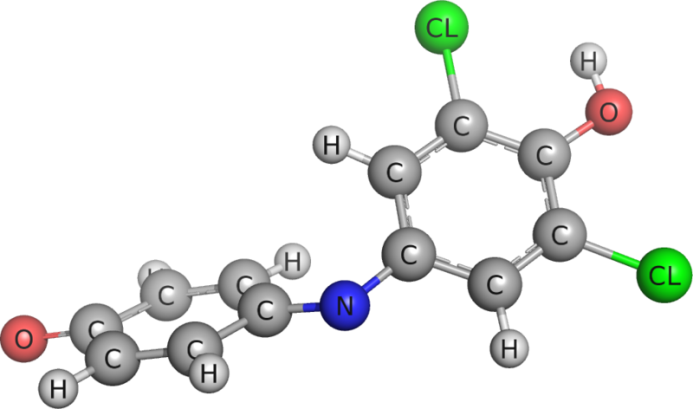  Anion |
| DCPIP(II) | 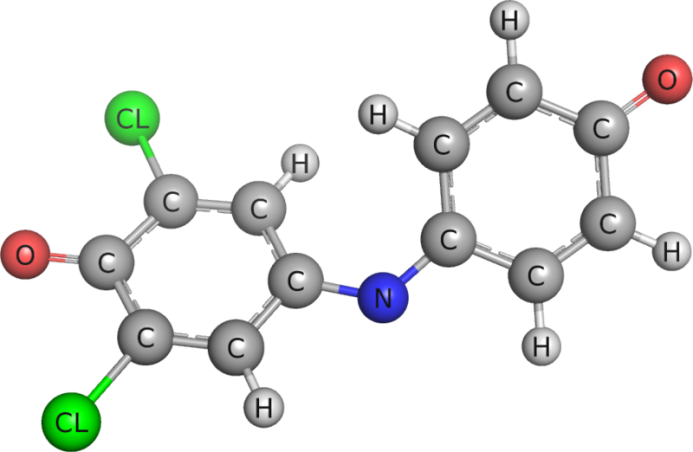  Anion | 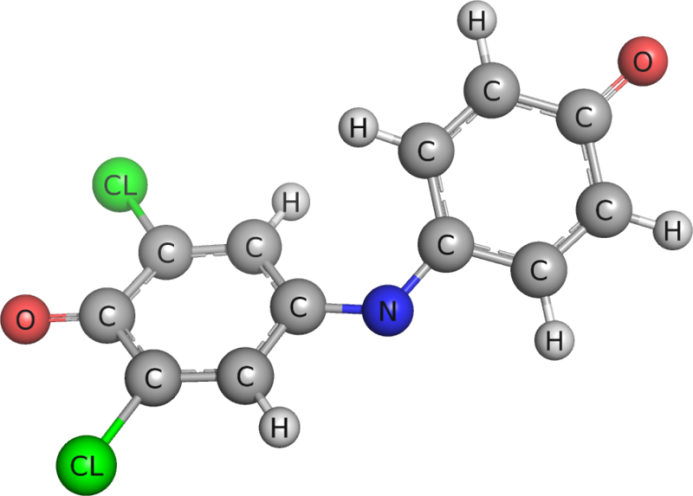  Radical |
| DMB | 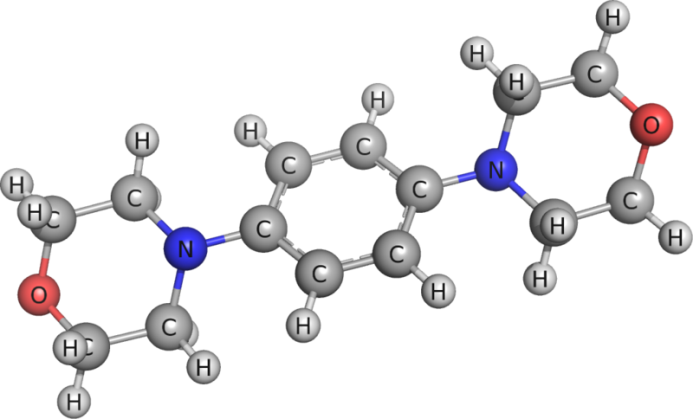  Neutral molecule | 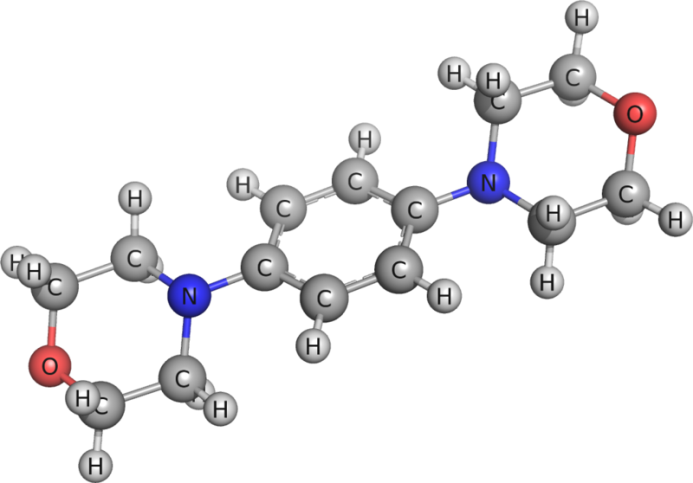  Radical cation |
| HEPX | 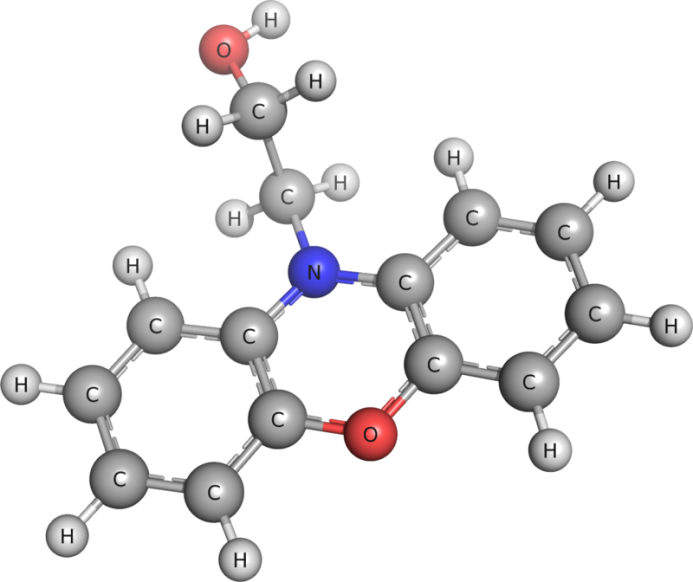  Neutral molecule | 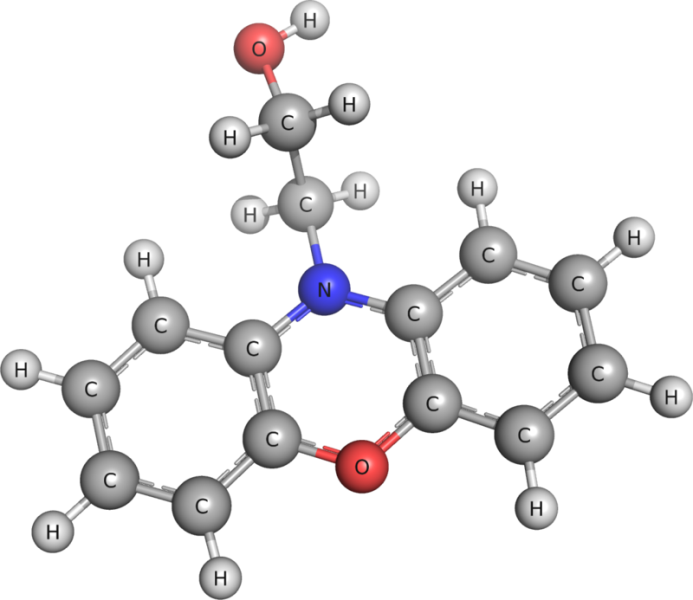  Radical cation |
| MB  (fully reduced) | 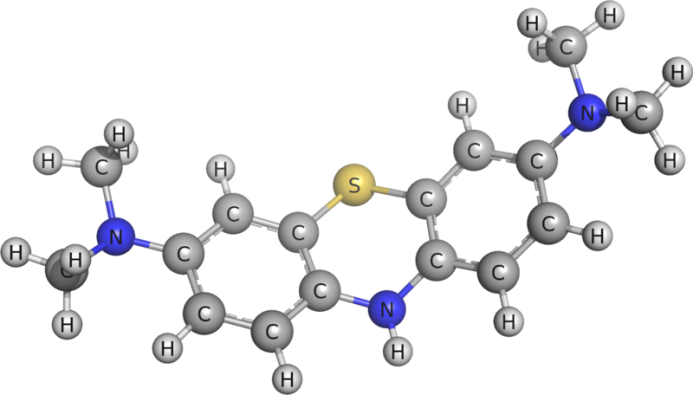  Neutral molecule | 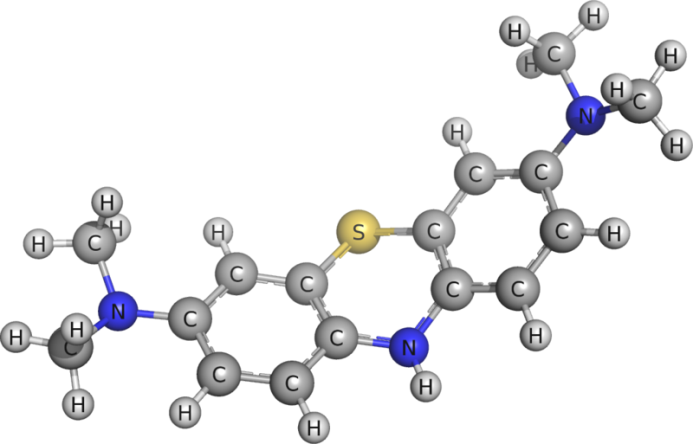  Radical cation |
| MB  (fully oxidized) | 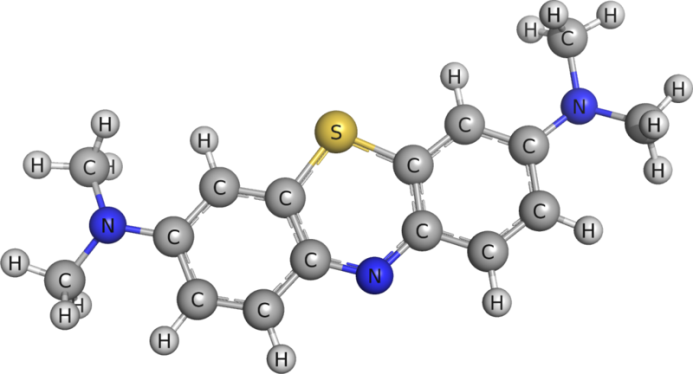  Radical | 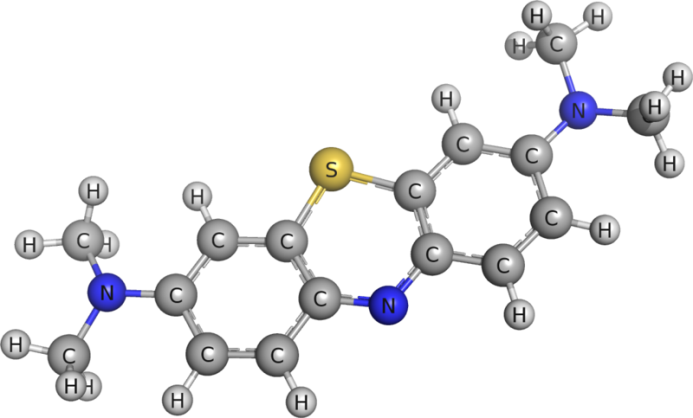  Cation |
| PPSA | 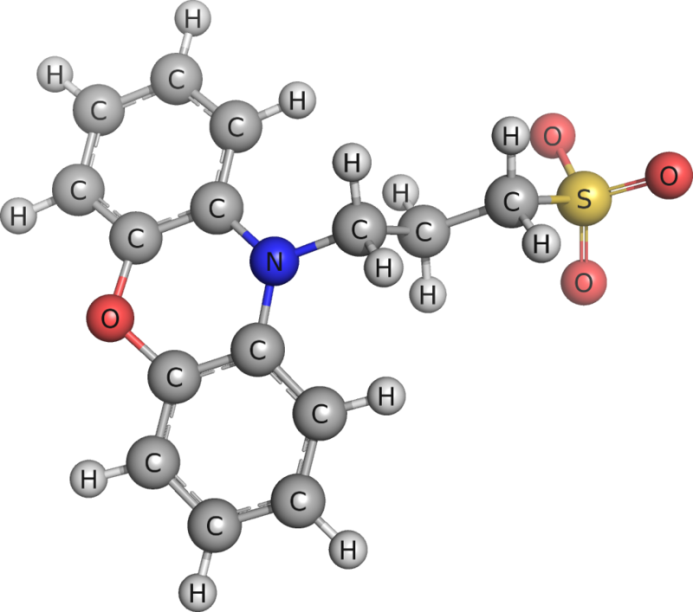  Anion | 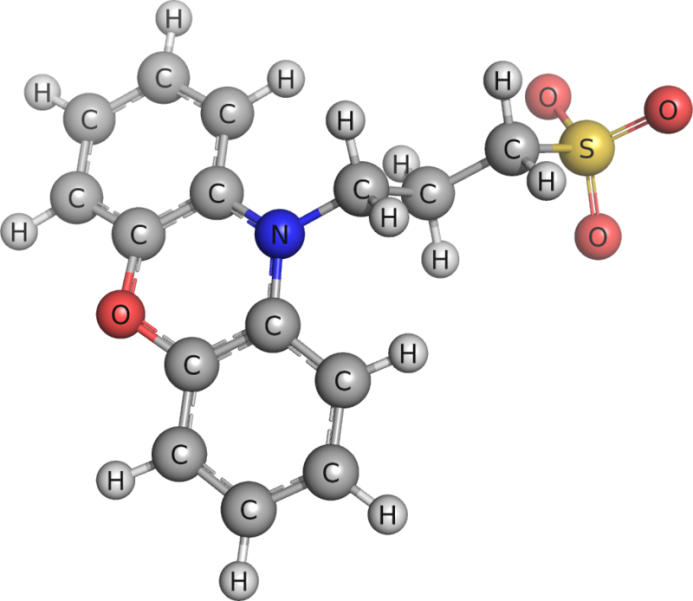  Radical |
| PZ | 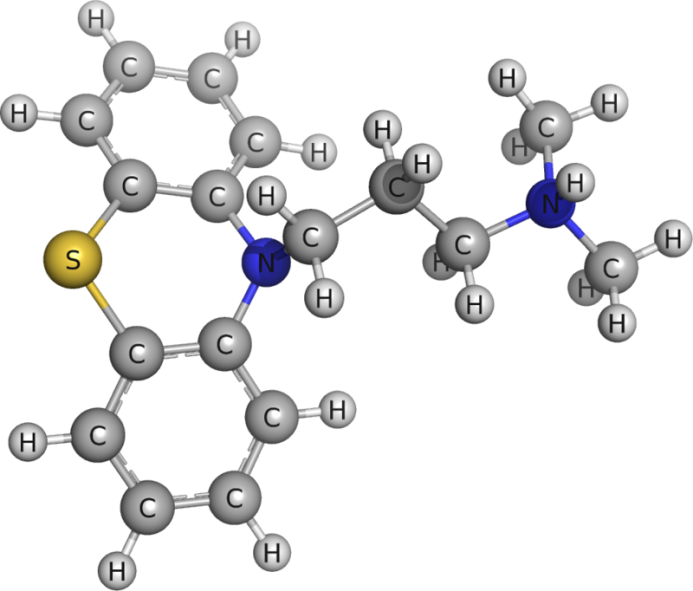  Cation | 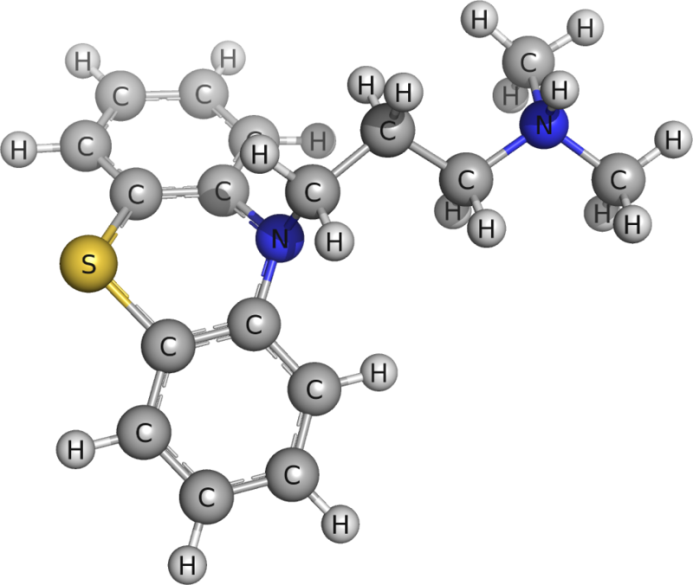  Radical di-cation |
| TH  (fully reduced) | 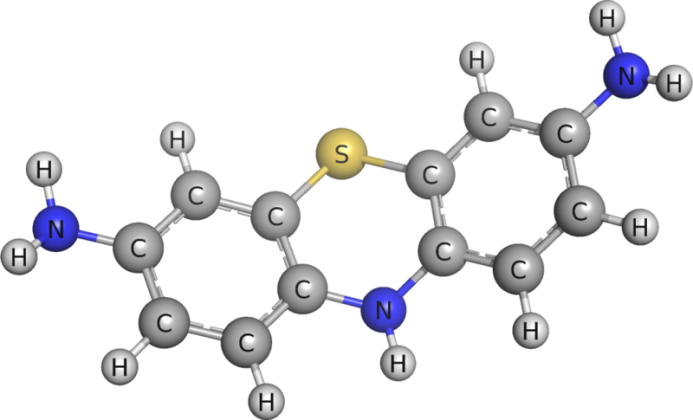  Neutral molecule | 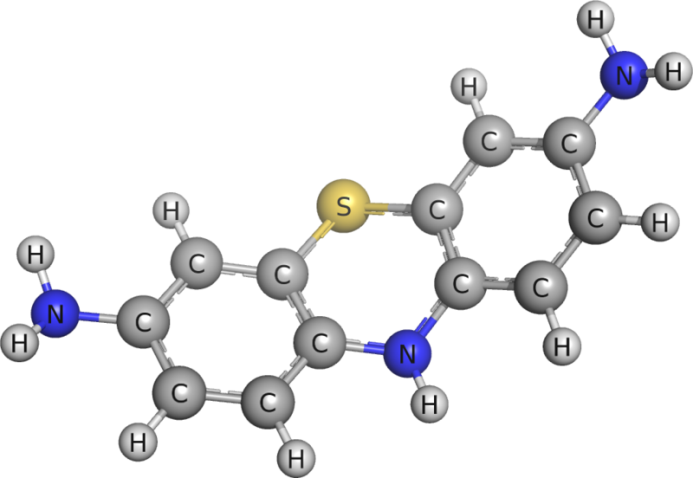  Radical cation |
| TH  (fully oxidized) | 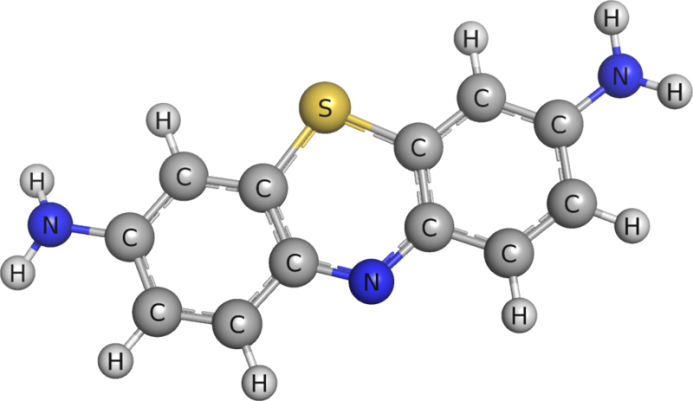  Radical | 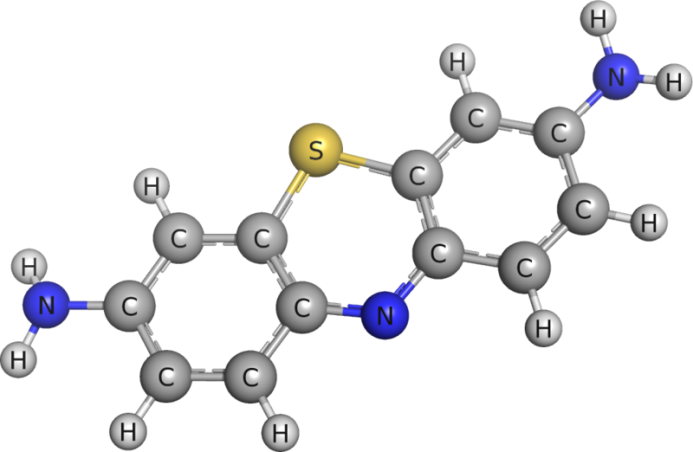  Cation |
| TMPD | 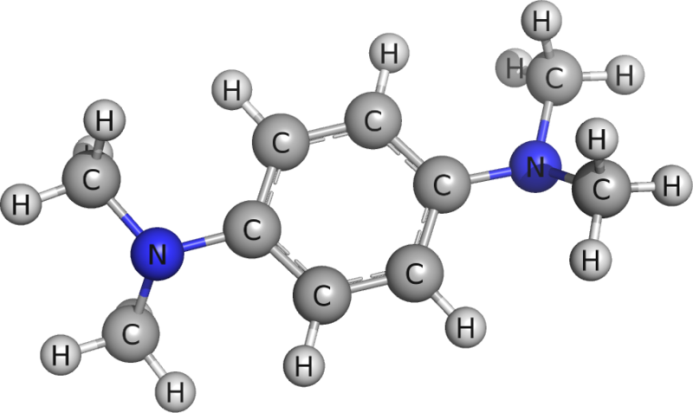  Neutral molecule | 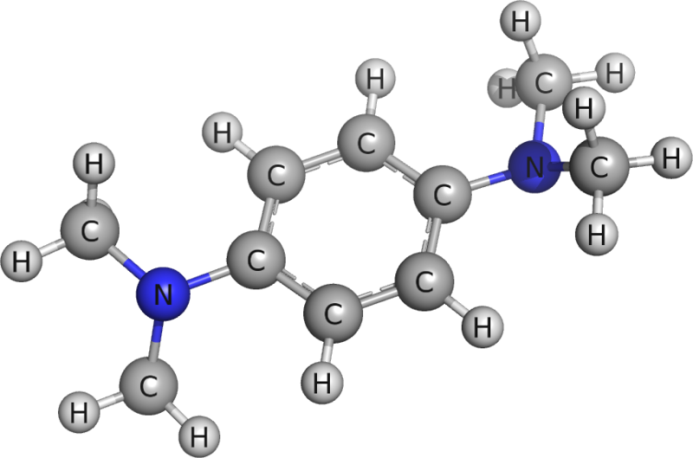  Radical cation |
| VB  (fully reduced) | 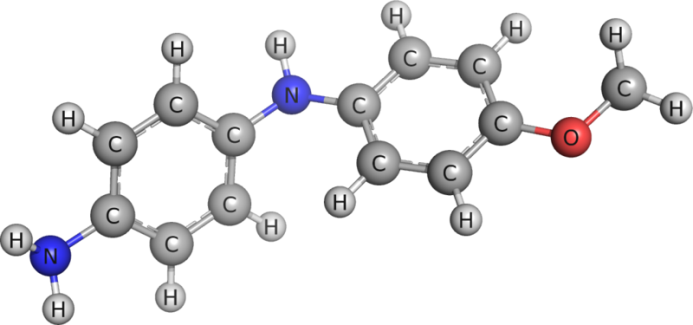  Neutral molecule | 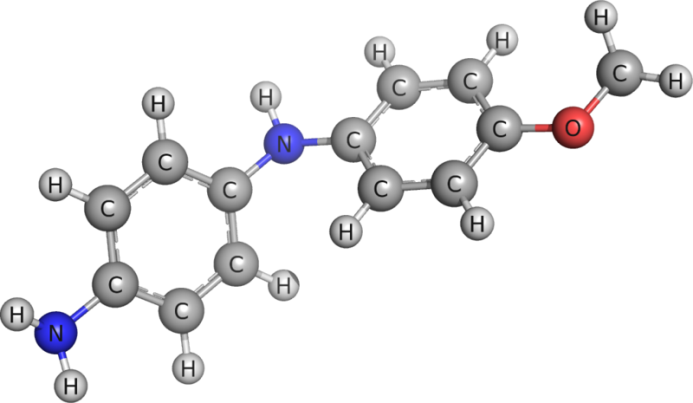  Radical cation |
| VB  (fully oxidized) | 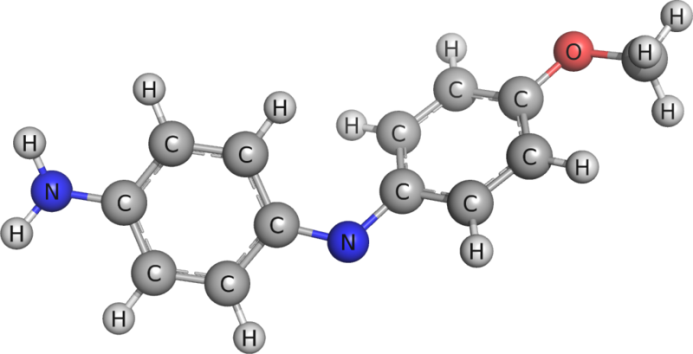  Radical | 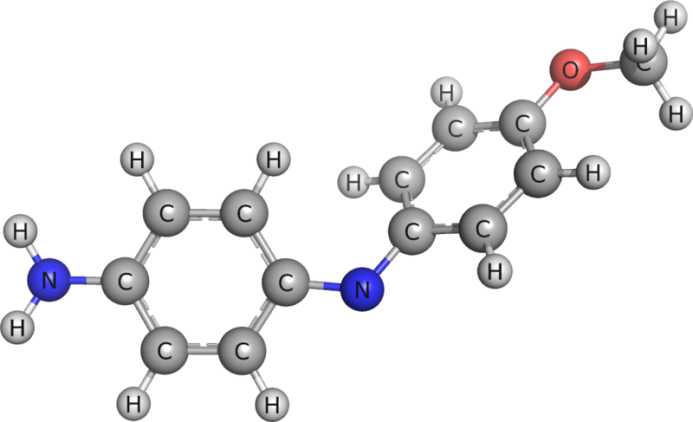  Cation |

| Table S18. Coordinates (X, Y and Z) of optimized ABTS structures, expressed in Å. | | | | | | | |
| --- | --- | --- | --- | --- | --- | --- | --- |
|  |  | Neutral |  |  | Radical cation | | |
| N | 0.49748 | 0.17467 | -0.45526 | C | 2.61439 | 2.54828 | -2.14672 |
| N | -0.49728 | -0.17533 | 0.45475 | C | 2.59708 | 1.02483 | -2.03994 |
| C | 1.68513 | 0.11441 | 0.04858 | N | 2.71456 | 0.55198 | -0.63948 |
| C | -1.68497 | -0.11488 | -0.04896 | C | 1.63462 | 0.26341 | 0.13098 |
| S | 2.06884 | -0.35219 | 1.73335 | S | 2.0763 | -0.21449 | 1.76497 |
| S | -2.06889 | 0.35218 | -1.73356 | C | 3.7758 | -0.0063 | 1.35887 |
| C | 3.79212 | -0.09637 | 1.41053 | C | 4.8798 | -0.2391 | 2.18043 |
| C | -3.79215 | 0.09642 | -1.41057 | C | 6.14313 | -0.03085 | 1.63475 |
| C | 4.84552 | -0.25206 | 2.30225 | C | 6.30849 | 0.41312 | 0.30928 |
| C | -4.84565 | 0.25238 | -2.30211 | C | 5.20868 | 0.64655 | -0.50544 |
| C | 6.14288 | 0.01075 | 1.84643 | C | 3.93046 | 0.42108 | 0.02577 |
| C | -6.14297 | -0.01046 | -1.84618 | S | 7.62576 | -0.40695 | 2.60384 |
| C | 6.37372 | 0.41566 | 0.52654 | O | 7.15739 | -0.73507 | 3.98433 |
| C | -6.37366 | -0.41562 | -0.52635 | O | 8.47794 | 0.81919 | 2.54966 |
| C | 5.31131 | 0.57078 | -0.36675 | O | 8.25353 | -1.56965 | 1.89539 |
| C | -5.31114 | -0.571 | 0.36677 | N | 0.40243 | 0.31722 | -0.35979 |
| C | 4.00907 | 0.30985 | 0.07868 | N | -0.55412 | 0.04158 | 0.52629 |
| C | -4.00894 | -0.31008 | -0.07878 | C | -1.7629 | 0.01248 | -0.0308 |
| N | 2.83638 | 0.39617 | -0.66484 | S | -2.07679 | 0.22328 | -1.74708 |
| N | -2.83615 | -0.39667 | 0.66456 | C | -3.79503 | 0.01597 | -1.44615 |
| C | 2.78745 | 0.84888 | -2.06474 | C | -4.82398 | 0.07734 | -2.38244 |
| C | -2.78706 | -0.84992 | 2.06429 | C | -6.12728 | -0.09673 | -1.9244 |
| C | 2.80495 | 2.37012 | -2.2103 | C | -6.40094 | -0.33355 | -0.56552 |
| C | -2.80463 | -2.37121 | 2.20926 | C | -5.37119 | -0.39631 | 0.36836 |
| S | 7.53429 | -0.24954 | 2.96251 | C | -4.05502 | -0.21157 | -0.08102 |
| S | -7.53451 | 0.25013 | -2.96205 | N | -2.89151 | -0.20393 | 0.68936 |
| O | 8.49884 | 0.87241 | 2.7263 | C | -2.87363 | -0.44928 | 2.15222 |
| O | -8.49912 | -0.87177 | -2.72583 | C | -2.90627 | -1.93635 | 2.49848 |
| O | 8.12516 | -1.58509 | 2.59038 | S | -7.48202 | 0.09072 | -3.1126 |
| O | -8.12514 | 1.58571 | -2.58973 | O | -8.43933 | -1.03442 | -2.87117 |
| O | 6.96456 | -0.2441 | 4.34724 | O | -8.08607 | 1.42983 | -2.79956 |
| O | -6.96498 | 0.2448 | -4.34687 | O | -6.82592 | 0.02955 | -4.45319 |
| H | 4.67601 | -0.56133 | 3.32755 | H | 2.5454 | 2.82625 | -3.2033 |
| H | -4.67625 | 0.56191 | -3.32735 | H | 1.76325 | 2.99005 | -1.61978 |
| H | 7.38386 | 0.61873 | 0.18637 | H | 3.53539 | 2.97786 | -1.74105 |
| H | -7.38377 | -0.6187 | -0.1861 | H | 3.41628 | 0.56656 | -2.59935 |
| H | 5.50644 | 0.89544 | -1.38277 | H | 1.66834 | 0.61388 | -2.43388 |
| H | -5.50617 | -0.89588 | 1.38274 | H | 4.76739 | -0.58018 | 3.20342 |
| H | 1.87709 | 0.43145 | -2.49706 | H | 7.30519 | 0.57214 | -0.08881 |
| H | -1.87661 | -0.4327 | 2.49663 | H | 5.3516 | 0.9887 | -1.52415 |
| H | 3.63223 | 0.39048 | -2.5885 | H | -4.62997 | 0.25998 | -3.43274 |
| H | -3.63173 | -0.39166 | 2.58834 | H | -7.42461 | -0.46684 | -0.23188 |
| H | 1.93767 | 2.82117 | -1.71869 | H | -5.59408 | -0.57558 | 1.41392 |
| H | -1.93745 | -2.82212 | 1.71736 | H | -1.97085 | 0.02243 | 2.53876 |
| H | 2.77053 | 2.63195 | -3.27333 | H | -3.72658 | 0.08716 | 2.57654 |
| H | -2.7701 | -2.63346 | 3.27218 | H | -2.03507 | -2.45522 | 2.08756 |
| H | 3.7115 | 2.81004 | -1.78225 | H | -2.88753 | -2.04264 | 3.58791 |
| H | -3.71127 | -2.81091 | 1.78115 | H | -3.81017 | -2.42693 | 2.12493 |

| Table S19. Coordinates (X, Y and Z) of optimized AMB structures, expressed in Å. | | | | | | | |
| --- | --- | --- | --- | --- | --- | --- | --- |
|  |  | Neutral |  |  | Radical cation | | |
| C | 4.43614 | 0.17521 | 0.93526 | C | 4.441 | -0.59541 | 0.68879 |
| N | 3.63775 | -0.0463 | -0.2814 | N | 3.57627 | 0.26628 | -0.12881 |
| C | 4.16884 | 0.74214 | -1.39293 | C | 4.21229 | 1.21314 | -1.05365 |
| C | 2.22921 | 0.02337 | -0.09009 | C | 2.2271 | 0.16908 | -0.04989 |
| C | 1.38242 | 0.79496 | -0.90049 | C | 1.36833 | 0.9326 | -0.90076 |
| C | -0.00979 | 0.78837 | -0.72568 | C | 0.00123 | 0.83905 | -0.80691 |
| C | -0.61785 | 0.03339 | 0.28639 | C | -0.63324 | -0.02297 | 0.14325 |
| C | 0.23532 | -0.73308 | 1.10955 | C | 0.23091 | -0.79954 | 0.97944 |
| C | 1.61112 | -0.74587 | 0.92155 | C | 1.59705 | -0.70545 | 0.88986 |
| N | -2.02261 | 0.04276 | 0.5364 | N | -1.99362 | -0.09637 | 0.24783 |
| C | -2.65061 | -1.29612 | 0.5432 | C | -2.65487 | -1.24707 | 0.89882 |
| C | -4.09863 | -1.21159 | 1.00431 | C | -4.0482 | -0.89368 | 1.40925 |
| O | -4.85524 | -0.31365 | 0.18343 | O | -4.85404 | -0.31935 | 0.381 |
| C | -4.26479 | 0.99175 | 0.18997 | C | -4.25311 | 0.89398 | -0.0687 |
| C | -2.81788 | 0.95798 | -0.29039 | C | -2.87099 | 0.65542 | -0.67203 |
| H | 4.11855 | -0.4809 | 1.74603 | H | 4.23176 | -1.65256 | 0.49617 |
| H | 5.48476 | -0.05166 | 0.72383 | H | 5.48062 | -0.40476 | 0.42952 |
| H | 4.37096 | 1.21813 | 1.28809 | H | 4.31223 | -0.38612 | 1.75619 |
| H | 5.23891 | 0.53972 | -1.48476 | H | 5.28174 | 1.24038 | -0.85249 |
| H | 3.69489 | 0.44915 | -2.33349 | H | 4.06397 | 0.91251 | -2.09721 |
| H | 4.04124 | 1.82958 | -1.25633 | H | 3.81973 | 2.22379 | -0.90687 |
| H | 1.78589 | 1.41497 | -1.69265 | H | 1.78441 | 1.58579 | -1.65832 |
| H | -0.60272 | 1.39721 | -1.39884 | H | -0.58961 | 1.41777 | -1.50467 |
| H | -0.17978 | -1.32476 | 1.92032 | H | -0.17343 | -1.47448 | 1.72203 |
| H | 2.21228 | -1.37543 | 1.57019 | H | 2.19669 | -1.3029 | 1.56534 |
| H | -2.61118 | -1.74428 | -0.46528 | H | -2.72288 | -2.06766 | 0.17116 |
| H | -2.11021 | -1.9616 | 1.21967 | H | -2.06839 | -1.59526 | 1.74736 |
| H | -4.57742 | -2.19084 | 0.91954 | H | -4.54751 | -1.80669 | 1.74226 |
| H | -4.15158 | -0.88175 | 2.05279 | H | -3.98543 | -0.19889 | 2.2598 |
| H | -4.86823 | 1.60809 | -0.48173 | H | -4.9061 | 1.31578 | -0.83624 |
| H | -4.32271 | 1.4159 | 1.20385 | H | -4.19457 | 1.60281 | 0.77011 |
| H | -2.79842 | 0.66627 | -1.35546 | H | -2.96242 | 0.10397 | -1.61801 |
| H | -2.40816 | 1.97021 | -0.21113 | H | -2.43767 | 1.63389 | -0.88228 |

| Table S20. Coordinates (X, Y and Z) of optimized CPZ structures, expressed in Å. | | | | | | | |
| --- | --- | --- | --- | --- | --- | --- | --- |
|  |  | Cation |  | Radical di-cation | | | |
| C | 4.49021 | -0.97995 | 0.08122 | C | 4.21248 | -1.59502 | -0.65141 |
| N | 3.64588 | 0.04674 | 0.79346 | N | 3.45539 | -0.37506 | -0.19066 |
| C | 3.888 | 1.42399 | 0.2335 | C | 3.40748 | 0.65008 | -1.29504 |
| C | 2.18288 | -0.36457 | 0.75053 | C | 2.07162 | -0.76373 | 0.31384 |
| C | 1.24766 | 0.53552 | 1.55423 | C | 1.37756 | 0.39017 | 1.04708 |
| C | -0.17337 | -0.04666 | 1.61376 | C | 0.0415 | 0.01661 | 1.72944 |
| N | -0.87417 | -0.09027 | 0.29461 | N | -1.16659 | 0.09802 | 0.86397 |
| C | -1.47373 | 1.15922 | -0.09261 | C | -1.7171 | 1.37442 | 0.67605 |
| C | -0.71936 | 2.10091 | -0.80468 | C | -1.08595 | 2.52706 | 1.21096 |
| C | -1.25385 | 3.34975 | -1.13132 | C | -1.62143 | 3.792 | 1.03081 |
| C | -2.57011 | 3.66006 | -0.77552 | C | -2.81424 | 3.98041 | 0.31123 |
| C | -3.3539 | 2.71334 | -0.11249 | C | -3.45714 | 2.87963 | -0.22157 |
| C | -2.80609 | 1.47202 | 0.23405 | C | -2.92466 | 1.58784 | -0.04779 |
| S | -3.81769 | 0.27498 | 1.09113 | S | -3.85976 | 0.31018 | -0.7576 |
| C | -3.08353 | -1.21831 | 0.44803 | C | -2.91887 | -1.10266 | -0.40641 |
| C | -3.86553 | -2.36989 | 0.29516 | C | -3.45101 | -2.31133 | -0.89651 |
| C | -3.29344 | -3.55803 | -0.1619 | C | -2.81138 | -3.51522 | -0.68034 |
| C | -1.93847 | -3.56518 | -0.4916 | C | -1.60416 | -3.49645 | 0.03559 |
| Cl | -1.20262 | -5.05193 | -1.09424 | Cl | -0.77115 | -5.00479 | 0.33612 |
| C | -1.1513 | -2.42217 | -0.37192 | C | -1.05298 | -2.32381 | 0.52075 |
| C | -1.71611 | -1.23936 | 0.12238 | C | -1.70054 | -1.08016 | 0.32975 |
| H | 5.53827 | -0.69278 | 0.16534 | H | 5.22631 | -1.30015 | -0.92459 |
| H | 4.33284 | -1.9533 | 0.54625 | H | 4.24109 | -2.33004 | 0.15305 |
| H | 4.19598 | -1.00971 | -0.96909 | H | 3.70212 | -2.0146 | -1.52041 |
| H | 3.9444 | 0.07123 | 1.7742 | H | 3.98299 | 0.03748 | 0.5938 |
| H | 3.35463 | 2.16103 | 0.83196 | H | 2.93197 | 1.56353 | -0.94212 |
| H | 3.54303 | 1.45197 | -0.80203 | H | 2.85245 | 0.23496 | -2.13806 |
| H | 4.95632 | 1.63536 | 0.27473 | H | 4.43069 | 0.87548 | -1.59733 |
| H | 1.90773 | -0.39412 | -0.30635 | H | 1.52032 | -1.11272 | -0.56395 |
| H | 2.15571 | -1.38469 | 1.14283 | H | 2.22959 | -1.60586 | 0.99273 |
| H | 1.20904 | 1.54862 | 1.14337 | H | 1.21722 | 1.23523 | 0.37379 |
| H | 1.59732 | 0.62128 | 2.5907 | H | 2.03503 | 0.73144 | 1.85592 |
| H | -0.75921 | 0.53701 | 2.33642 | H | -0.13617 | 0.67985 | 2.57566 |
| H | -0.13256 | -1.07139 | 1.99498 | H | 0.08138 | -0.96997 | 2.18377 |
| H | 0.28997 | 1.84334 | -1.10995 | H | -0.16102 | 2.45846 | 1.76328 |
| H | -0.64817 | 4.0713 | -1.67213 | H | -1.10164 | 4.64535 | 1.45415 |
| H | -2.99628 | 4.62511 | -1.03444 | H | -3.22653 | 4.97454 | 0.1707 |
| H | -4.3886 | 2.93676 | 0.13321 | H | -4.37969 | 3.00193 | -0.78259 |
| H | -4.92486 | -2.3477 | 0.5358 | H | -4.38764 | -2.28974 | -1.44637 |
| H | -3.89605 | -4.45348 | -0.26964 | H | -3.22725 | -4.44389 | -1.05448 |
| H | -0.10395 | -2.43922 | -0.65252 | H | -0.11953 | -2.39895 | 1.05182 |

| Table S21. Coordinates (X, Y and Z) of optimized DCPIP(I) (2^nd^ electron reduction) structures, expressed in Å. | | | | | | | |
| --- | --- | --- | --- | --- | --- | --- | --- |
|  |  | Anion |  |  |  | Radical |  |
| Cl | -4.22164 | -1.7434 | 0.0869 | Cl | -3.64697 | 2.3058 | -0.4415 |
| C | -2.70428 | -0.86148 | -0.03228 | C | -2.44489 | 1.09703 | -0.03501 |
| C | -1.51987 | -1.57597 | -0.16988 | C | -1.1706 | 1.50098 | 0.3515 |
| C | -0.28664 | -0.9011 | -0.26624 | C | -0.21019 | 0.5376 | 0.67863 |
| N | 0.89436 | -1.62608 | -0.45155 | N | 1.08711 | 0.9541 | 1.08011 |
| C | 2.18281 | -1.0224 | -0.21742 | C | 2.27392 | 0.54673 | 0.53451 |
| C | 2.96404 | -0.57806 | -1.29235 | C | 3.489 | 0.93786 | 1.17589 |
| C | 4.21892 | -0.00375 | -1.08448 | C | 4.70608 | 0.58728 | 0.65167 |
| C | 4.77686 | 0.14812 | 0.2235 | C | 4.80815 | -0.1847 | -0.57056 |
| O | 5.96694 | 0.66367 | 0.42105 | O | 5.93584 | -0.50434 | -1.07341 |
| C | 3.94895 | -0.30328 | 1.30177 | C | 3.56016 | -0.56727 | -1.19695 |
| C | 2.69512 | -0.87149 | 1.08091 | C | 2.34268 | -0.21413 | -0.66946 |
| C | -0.29181 | 0.50368 | -0.21936 | C | -0.53903 | -0.82225 | 0.63854 |
| C | -1.49636 | 1.1877 | -0.08289 | C | -1.8154 | -1.20152 | 0.23753 |
| Cl | -1.47205 | 2.95197 | -0.02274 | Cl | -2.24107 | -2.90684 | 0.2018 |
| C | -2.73304 | 0.53977 | 0.01842 | C | -2.79126 | -0.26044 | -0.12945 |
| O | -3.93727 | 1.18717 | 0.16467 | O | -4.04292 | -0.58954 | -0.55047 |
| H | -1.5555 | -2.66067 | -0.20285 | H | -0.9226 | 2.55692 | 0.37779 |
| H | 0.8273 | -2.59444 | -0.15387 | H | 1.1441 | 1.60802 | 1.85447 |
| H | 2.58257 | -0.68735 | -2.3053 | H | 3.43654 | 1.51599 | 2.09556 |
| H | 4.79889 | 0.31515 | -1.94671 | H | 5.62649 | 0.88441 | 1.14561 |
| H | 4.31911 | -0.2001 | 2.3192 | H | 3.61013 | -1.13533 | -2.12108 |
| H | 2.09871 | -1.20337 | 1.92943 | H | 1.429 | -0.49574 | -1.18229 |
| H | 0.63628 | 1.05797 | -0.28568 | H | 0.18245 | -1.57514 | 0.93617 |
| H | -3.79876 | 2.14768 | 0.21332 | H | -4.13585 | -1.55083 | -0.66899 |

| Table S22. Coordinates (X, Y and Z) of optimized DCPIP(I) (1^st^ electron reduction) structures, expressed in Å. | | | | | | | |
| --- | --- | --- | --- | --- | --- | --- | --- |
| Radical anion | | | |  |  | Anion |  |
| Cl | 3.88042 | -2.14094 | -0.3374 | Cl | -3.75134 | 2.27868 | -0.2139 |
| C | 2.54678 | -1.03398 | -0.03531 | C | -2.47647 | 1.10247 | 0.03641 |
| C | 1.28444 | -1.54666 | 0.23126 | C | -1.20178 | 1.5337 | 0.3713 |
| C | 0.18277 | -0.68921 | 0.45863 | C | -0.15986 | 0.60154 | 0.53598 |
| N | -1.03414 | -1.25919 | 0.79276 | N | 1.09299 | 1.09753 | 0.90535 |
| C | -2.21102 | -0.73675 | 0.38067 | C | 2.2144 | 0.64909 | 0.41392 |
| C | -3.40842 | -1.20821 | 1.0192 | C | 3.44296 | 1.13671 | 1.04713 |
| C | -4.65859 | -0.76058 | 0.66101 | C | 4.66083 | 0.69152 | 0.67026 |
| C | -4.84725 | 0.19269 | -0.40489 | C | 4.80287 | -0.26516 | -0.43609 |
| O | -6.01728 | 0.60485 | -0.75672 | O | 5.91984 | -0.68225 | -0.79553 |
| C | -3.64756 | 0.63126 | -1.07329 | C | 3.57305 | -0.68751 | -1.12897 |
| C | -2.39562 | 0.19398 | -0.69993 | C | 2.35542 | -0.25904 | -0.73137 |
| C | 0.43228 | 0.70443 | 0.44784 | C | -0.45026 | -0.77301 | 0.43956 |
| C | 1.70957 | 1.18754 | 0.19364 | C | -1.74499 | -1.18138 | 0.14132 |
| Cl | 1.99453 | 2.9325 | 0.20695 | Cl | -2.11119 | -2.89902 | 0.03928 |
| C | 2.79996 | 0.34542 | -0.06794 | C | -2.78378 | -0.26448 | -0.08857 |
| O | 4.06944 | 0.79036 | -0.33673 | O | -4.0576 | -0.62083 | -0.40132 |
| H | 1.13163 | -2.62018 | 0.25345 | H | -0.99697 | 2.59286 | 0.48431 |
| H | -3.29545 | -1.93349 | 1.82128 | H | 3.32454 | 1.8383 | 1.86756 |
| H | -5.54238 | -1.13021 | 1.17577 | H | 5.57089 | 1.02095 | 1.16252 |
| H | -3.75751 | 1.32048 | -1.90748 | H | 3.69321 | -1.34417 | -1.98541 |
| H | -1.53079 | 0.53207 | -1.26256 | H | 1.46753 | -0.53851 | -1.28774 |
| H | -0.36311 | 1.40383 | 0.67699 | H | 0.31207 | -1.51379 | 0.65248 |
| H | 4.08962 | 1.7618 | -0.3469 | H | -4.15278 | -1.58738 | -0.46049 |

| Table S23. Coordinates (X, Y and Z) of optimized DCPIP(II) structures, expressed in Å. | | | | | | | |
| --- | --- | --- | --- | --- | --- | --- | --- |
|  |  | Anion |  |  |  | Radical |  |
| O | 4.85158 | 0.54516 | 0.91221 | O | 4.73009 | 0.31285 | 1.04928 |
| C | 3.69626 | 0.30634 | 0.45656 | C | 3.61022 | 0.17533 | 0.52582 |
| C | 2.53415 | 0.22122 | 1.33302 | C | 2.42144 | -0.12861 | 1.34168 |
| C | 1.28015 | -0.00401 | 0.84644 | C | 1.19945 | -0.25684 | 0.77843 |
| C | 1.04853 | -0.19613 | -0.57033 | C | 1.02343 | -0.09805 | -0.66469 |
| C | 2.21318 | -0.21129 | -1.42759 | C | 2.20597 | 0.12547 | -1.48951 |
| C | 3.46354 | 0.05752 | -0.95719 | C | 3.42702 | 0.27684 | -0.93026 |
| N | -0.11859 | -0.43536 | -1.16641 | N | -0.12806 | -0.20216 | -1.28322 |
| C | -1.35503 | -0.23166 | -0.64417 | C | -1.3624 | -0.11235 | -0.73388 |
| C | -1.70372 | 0.6855 | 0.39656 | C | -1.69445 | 0.83682 | 0.28675 |
| C | -3.00896 | 0.81749 | 0.79865 | C | -2.97585 | 0.91529 | 0.75205 |
| Cl | -3.41126 | 2.0201 | 2.02423 | Cl | -3.39456 | 2.07257 | 1.98238 |
| C | -4.11804 | 0.04763 | 0.25142 | C | -4.06377 | 0.05874 | 0.2509 |
| O | -5.31232 | 0.17524 | 0.63689 | O | -5.2305 | 0.13927 | 0.67012 |
| C | -3.70757 | -0.84622 | -0.81777 | C | -3.65837 | -0.88349 | -0.80412 |
| Cl | -4.96489 | -1.81443 | -1.58183 | Cl | -4.89275 | -1.93739 | -1.43205 |
| C | -2.4187 | -0.9493 | -1.26992 | C | -2.38884 | -0.93697 | -1.29678 |
| H | 2.70884 | 0.31269 | 2.4017 | H | 2.57612 | -0.26178 | 2.40812 |
| H | 0.4557 | -0.12363 | 1.54132 | H | 0.33938 | -0.53115 | 1.3806 |
| H | 2.04651 | -0.40525 | -2.48345 | H | 2.05933 | 0.20069 | -2.56271 |
| H | 4.32155 | 0.08308 | -1.62324 | H | 4.31052 | 0.47819 | -1.52876 |
| H | -0.95282 | 1.34305 | 0.81823 | H | -0.93337 | 1.51612 | 0.65581 |
| H | -2.17245 | -1.61423 | -2.09129 | H | -2.12327 | -1.61802 | -2.09805 |

| Table S24. Coordinates (X, Y and Z) of optimized DMB structures, expressed in Å. | | | | | | | |
| --- | --- | --- | --- | --- | --- | --- | --- |
|  |  | Neutral |  | Radical cation | | | |
| C | 0.85075 | -1.35971 | 0.16208 | C | 0.71277 | -1.37615 | -0.22692 |
| C | -0.4478 | -1.57198 | 0.62956 | C | -0.61593 | -1.5508 | 0.07289 |
| C | -1.3636 | -0.51545 | 0.78111 | C | -1.39326 | -0.51226 | 0.67182 |
| C | -0.90729 | 0.77242 | 0.439 | C | -0.70682 | 0.70456 | 0.97354 |
| C | 0.391 | 0.98399 | -0.02766 | C | 0.62353 | 0.87566 | 0.6854 |
| C | 1.30404 | -0.07293 | -0.18441 | C | 1.39506 | -0.14933 | 0.05018 |
| N | 2.59813 | 0.17716 | -0.72675 | N | 2.70245 | 0.03955 | -0.28402 |
| C | 3.43549 | -0.99771 | -0.99273 | C | 3.6095 | -1.06227 | -0.66102 |
| C | 4.68615 | -0.59093 | -1.76454 | C | 4.58283 | -0.62878 | -1.75484 |
| O | 5.44193 | 0.40274 | -1.05954 | O | 5.31124 | 0.53814 | -1.37226 |
| C | 4.63682 | 1.56229 | -0.80823 | C | 4.41112 | 1.61959 | -1.13309 |
| C | 3.38425 | 1.20792 | -0.01915 | C | 3.42557 | 1.29506 | -0.01076 |
| N | -2.66572 | -0.76092 | 1.30637 | N | -2.71814 | -0.67703 | 0.95731 |
| C | -3.5264 | 0.41636 | 1.47546 | C | -3.64372 | 0.47002 | 1.0406 |
| C | -4.8109 | 0.04334 | 2.2055 | C | -4.81317 | 0.18601 | 1.9754 |
| O | -5.52582 | -0.99234 | 1.51946 | O | -5.49102 | -1.01676 | 1.60735 |
| C | -4.70339 | -2.15707 | 1.37958 | C | -4.59373 | -2.12424 | 1.68349 |
| C | -3.41419 | -1.84286 | 0.63276 | C | -3.40355 | -1.96474 | 0.73771 |
| H | 1.50677 | -2.21924 | 0.08665 | H | 1.22687 | -2.19394 | -0.7114 |
| H | -0.73093 | -2.58274 | 0.90659 | H | -1.07342 | -2.49189 | -0.20457 |
| H | -1.56144 | 1.63301 | 0.51169 | H | -1.21973 | 1.50957 | 1.48244 |
| H | 0.67634 | 1.99433 | -0.30531 | H | 1.08268 | 1.80636 | 0.9911 |
| H | 2.87636 | -1.71394 | -1.60278 | H | 3.05033 | -1.92284 | -1.01997 |
| H | 3.7371 | -1.50612 | -0.06006 | H | 4.16491 | -1.37325 | 0.23435 |
| H | 5.34929 | -1.45072 | -1.89196 | H | 5.31264 | -1.42465 | -1.92107 |
| H | 4.41424 | -0.20643 | -2.75884 | H | 4.0475 | -0.44142 | -2.69705 |
| H | 5.25992 | 2.2557 | -0.23694 | H | 5.01387 | 2.48229 | -0.839 |
| H | 4.36705 | 2.03506 | -1.7644 | H | 3.87348 | 1.86777 | -2.05989 |
| H | 2.78892 | 2.11445 | 0.10836 | H | 2.73039 | 2.12672 | 0.07653 |
| H | 3.67091 | 0.85322 | 0.98648 | H | 3.96242 | 1.19877 | 0.94266 |
| H | -3.78464 | 0.87486 | 0.50484 | H | -4.01806 | 0.6791 | 0.02837 |
| H | -3.00379 | 1.16728 | 2.07589 | H | -3.12533 | 1.36032 | 1.39054 |
| H | -5.48191 | 0.90497 | 2.24982 | H | -5.538 | 1.00015 | 1.90249 |
| H | -4.5902 | -0.28317 | 3.23273 | H | -4.46972 | 0.11447 | 3.01752 |
| H | -4.47963 | -2.56882 | 2.37508 | H | -4.24609 | -2.24509 | 2.72 |
| H | -5.29182 | -2.88972 | 0.82073 | H | -5.15686 | -3.01541 | 1.39734 |
| H | -2.81223 | -2.75288 | 0.59697 | H | -2.71589 | -2.78785 | 0.93629 |
| H | -3.65057 | -1.55788 | -0.40731 | H | -3.73793 | -2.02767 | -0.30677 |

| Table S25. Coordinates (X, Y and Z) of optimized HEPX structures, expressed in Å. | | | | | | | |
| --- | --- | --- | --- | --- | --- | --- | --- |
|  |  | Neutral |  | Radical cation | | | |
| N | -0.02001 | 0.45745 | -0.31922 | N | -0.01283 | 0.45222 | -0.27309 |
| C | -1.23966 | -0.21103 | -0.10033 | C | -1.23014 | -0.20542 | -0.11784 |
| C | -2.4739 | 0.44431 | 0.0346 | C | -2.47783 | 0.45769 | -0.12716 |
| C | -3.65919 | -0.28247 | 0.21447 | C | -3.65192 | -0.26568 | 0.00477 |
| C | -3.63325 | -1.6744 | 0.28888 | C | -3.62692 | -1.66782 | 0.14682 |
| C | -2.40737 | -2.34348 | 0.17021 | C | -2.4152 | -2.3421 | 0.1741 |
| C | -1.24136 | -1.61852 | -0.03005 | C | -1.23267 | -1.61314 | 0.05333 |
| O | -0.06645 | -2.34358 | -0.21641 | O | -0.06617 | -2.31468 | 0.11657 |
| C | 1.13529 | -1.65838 | -0.04621 | C | 1.12503 | -1.65321 | 0.07915 |
| C | 2.27941 | -2.42156 | 0.13712 | C | 2.27708 | -2.42516 | 0.22366 |
| C | 3.52701 | -1.79296 | 0.25236 | C | 3.51515 | -1.8012 | 0.17847 |
| C | 3.59586 | -0.40199 | 0.19121 | C | 3.59712 | -0.40789 | -0.01413 |
| C | 2.43357 | 0.36232 | 0.02031 | C | 2.45321 | 0.35965 | -0.165 |
| C | 1.17856 | -0.25186 | -0.11169 | C | 1.17954 | -0.2501 | -0.12395 |
| C | -0.00113 | 1.88455 | -0.62964 | C | 0.00721 | 1.89484 | -0.58439 |
| C | 0.02861 | 2.81368 | 0.59708 | C | 0.03124 | 2.78562 | 0.67709 |
| O | -0.09977 | 4.18802 | 0.20081 | O | -0.13071 | 4.15323 | 0.30783 |
| H | -2.52567 | 1.52607 | -0.00688 | H | -2.5265 | 1.53354 | -0.22311 |
| H | -4.59885 | 0.25506 | 0.30515 | H | -4.60092 | 0.26072 | 0.00105 |
| H | -4.54835 | -2.24049 | 0.43516 | H | -4.55472 | -2.22254 | 0.24376 |
| H | -2.34563 | -3.42688 | 0.21369 | H | -2.35561 | -3.41839 | 0.29203 |
| H | 2.18641 | -3.50307 | 0.17396 | H | 2.17621 | -3.49535 | 0.36975 |
| H | 4.4236 | -2.3906 | 0.38711 | H | 4.41774 | -2.39211 | 0.2956 |
| H | 4.55196 | 0.10574 | 0.28142 | H | 4.56796 | 0.07645 | -0.0384 |
| H | 2.51977 | 1.44217 | -0.00854 | H | 2.55497 | 1.42856 | -0.29688 |
| H | 0.86432 | 2.08289 | -1.27186 | H | 0.87035 | 2.09431 | -1.22112 |
| H | -0.88297 | 2.11597 | -1.23426 | H | -0.86996 | 2.12305 | -1.18886 |
| H | -0.82151 | 2.61143 | 1.25467 | H | -0.80996 | 2.53687 | 1.33005 |
| H | 0.94081 | 2.66816 | 1.18776 | H | 0.95299 | 2.63555 | 1.25007 |
| H | 0.72501 | 4.48931 | -0.21174 | H | 0.71411 | 4.52851 | 0.01424 |

| Table S26. Coordinates (X, Y and Z) of optimized MB (fully reduced) structures, expressed in Å. | | | | | | | |
| --- | --- | --- | --- | --- | --- | --- | --- |
|  |  | Neutral |  |  |  | Radical |  |
| N | 0.05447 | -0.37968 | -2.13328 | N | 0.08693 | -0.90996 | -1.54488 |
| C | -1.1814 | -0.32218 | -1.44153 | C | -1.17996 | -0.61851 | -1.09036 |
| C | -2.22014 | -1.21803 | -1.71593 | C | -2.29636 | -1.22223 | -1.71135 |
| C | -3.45069 | -1.12967 | -1.06248 | C | -3.58138 | -0.9578 | -1.2923 |
| C | -3.68979 | -0.14513 | -0.07829 | C | -3.83077 | -0.05921 | -0.2093 |
| C | -2.62161 | 0.7279 | 0.22522 | C | -2.70583 | 0.54278 | 0.41321 |
| C | -1.40798 | 0.65611 | -0.46265 | C | -1.41247 | 0.27312 | -0.0146 |
| S | -0.1383 | 1.87533 | -0.13653 | S | -0.09207 | 1.09836 | 0.83153 |
| C | 1.30913 | 0.86629 | -0.4396 | C | 1.35525 | 0.50295 | -0.00035 |
| C | 2.48414 | 1.11949 | 0.27548 | C | 2.58221 | 0.98226 | 0.44424 |
| C | 3.67917 | 0.42368 | -0.0078 | C | 3.79869 | 0.57017 | -0.15659 |
| C | 3.61455 | -0.57573 | -1.00515 | C | 3.7138 | -0.38493 | -1.21366 |
| C | 2.42796 | -0.84815 | -1.68511 | C | 2.49491 | -0.85661 | -1.65186 |
| C | 1.25489 | -0.12982 | -1.42394 | C | 1.28552 | -0.42169 | -1.06886 |
| N | 4.89083 | 0.73335 | 0.6478 | N | 5.00602 | 1.07633 | 0.25068 |
| N | -4.9466 | -0.00732 | 0.54853 | N | -5.10079 | 0.21046 | 0.20835 |
| C | 5.86018 | -0.36243 | 0.76429 | C | 6.25109 | 0.45766 | -0.21235 |
| C | 4.78482 | 1.52286 | 1.8772 | C | 5.07796 | 1.8897 | 1.46561 |
| C | -5.83773 | -1.16929 | 0.49046 | C | -6.24973 | -0.40984 | -0.45403 |
| C | -4.95262 | 0.64874 | 1.86027 | C | -5.3341 | 1.10936 | 1.33933 |
| H | 0.11948 | -1.18633 | -2.74615 | H | 0.14127 | -1.52739 | -2.35214 |
| H | -2.07337 | -1.99715 | -2.46114 | H | -2.1333 | -1.90883 | -2.53826 |
| H | -4.21744 | -1.84702 | -1.3269 | H | -4.40043 | -1.44911 | -1.80108 |
| H | -2.73025 | 1.48747 | 0.98955 | H | -2.84587 | 1.22927 | 1.23929 |
| H | 2.45335 | 1.87937 | 1.04657 | H | 2.59068 | 1.684 | 1.26929 |
| H | 4.48998 | -1.15982 | -1.26185 | H | 4.61043 | -0.75964 | -1.68927 |
| H | 2.42031 | -1.6342 | -2.43769 | H | 2.47017 | -1.58092 | -2.46157 |
| H | 6.19838 | -0.69951 | -0.21812 | H | 6.3103 | 0.45764 | -1.304 |
| H | 5.45872 | -1.22914 | 1.31376 | H | 6.3605 | -0.57296 | 0.15231 |
| H | 6.74334 | 0.00288 | 1.29272 | H | 7.09412 | 1.04198 | 0.15637 |
| H | 4.34475 | 2.50341 | 1.67775 | H | 4.42939 | 2.76745 | 1.38901 |
| H | 5.78804 | 1.69986 | 2.27036 | H | 6.09819 | 2.25255 | 1.59089 |
| H | 4.19071 | 1.02227 | 2.65955 | H | 4.80348 | 1.31731 | 2.36263 |
| H | -5.4058 | -2.06452 | 0.96706 | H | -6.24781 | -1.50005 | -0.33156 |
| H | -6.09822 | -1.41514 | -0.5422 | H | -6.27226 | -0.17242 | -1.52375 |
| H | -6.77012 | -0.92256 | 1.00225 | H | -7.16669 | -0.02423 | -0.01066 |
| H | -4.32096 | 0.12981 | 2.59949 | H | -4.83207 | 0.7507 | 2.24601 |
| H | -4.62199 | 1.68756 | 1.78552 | H | -4.99031 | 2.12844 | 1.12261 |
| H | -5.97679 | 0.67089 | 2.23781 | H | -6.40253 | 1.15298 | 1.54686 |

| Table S27. Coordinates (X, Y and Z) of optimized MB (fully oxidized) structures, expressed in Å. | | | | | | | |
| --- | --- | --- | --- | --- | --- | --- | --- |
|  |  | Radical |  |  |  | Cation |  |
| C | -5.40464 | 0.98828 | 0.76719 | C | -5.39857 | 0.91226 | 0.87333 |
| N | -5.07998 | -0.35083 | 0.27147 | N | -5.04921 | -0.27429 | 0.08315 |
| C | -6.16342 | -1.01938 | -0.45398 | C | -6.13511 | -1.13722 | -0.40122 |
| C | -3.769 | -0.61453 | -0.10532 | C | -3.7581 | -0.55751 | -0.19887 |
| C | -2.70415 | 0.22723 | 0.28968 | C | -2.70265 | 0.28529 | 0.25023 |
| C | -1.38053 | -0.06881 | -0.03506 | C | -1.38281 | -0.01724 | -0.03126 |
| C | -1.02936 | -1.22164 | -0.79694 | C | -1.02479 | -1.18322 | -0.79756 |
| C | -2.11829 | -2.04239 | -1.19995 | C | -2.10213 | -2.01369 | -1.24362 |
| C | -3.43153 | -1.76159 | -0.87893 | C | -3.40775 | -1.72563 | -0.96376 |
| N | 0.22646 | -1.60072 | -1.17251 | N | 0.22003 | -1.55192 | -1.12557 |
| C | 1.326 | -0.86627 | -0.83395 | C | 1.31554 | -0.86623 | -0.7772 |
| C | 2.59655 | -1.34316 | -1.25755 | C | 2.58122 | -1.3819 | -1.20315 |
| C | 3.7788 | -0.68733 | -0.97807 | C | 3.75924 | -0.75586 | -0.91258 |
| C | 3.78529 | 0.53123 | -0.24122 | C | 3.77455 | 0.47064 | -0.15739 |
| N | 4.96442 | 1.22496 | 0.00481 | N | 4.93775 | 1.10017 | 0.11939 |
| C | 4.95524 | 2.26714 | 1.03326 | C | 4.94707 | 2.36184 | 0.86904 |
| C | 6.23423 | 0.50639 | -0.13374 | C | 6.22346 | 0.52871 | -0.30472 |
| C | 2.53487 | 1.01615 | 0.20593 | C | 2.52767 | 0.99784 | 0.28427 |
| C | 1.34637 | 0.34571 | -0.08316 | C | 1.34017 | 0.35387 | -0.01126 |
| S | -0.14657 | 1.06166 | 0.54724 | S | -0.14732 | 1.06289 | 0.58519 |
| H | -4.83369 | 1.22442 | 1.66946 | H | -4.9218 | 0.88308 | 1.85928 |
| H | -5.21999 | 1.77186 | 0.01611 | H | -5.10747 | 1.83543 | 0.35903 |
| H | -6.46029 | 1.01858 | 1.04102 | H | -6.4763 | 0.9365 | 1.02406 |
| H | -6.19768 | -0.73516 | -1.51683 | H | -6.15462 | -1.17653 | -1.49554 |
| H | -7.11482 | -0.74724 | 0.0059 | H | -7.08589 | -0.73087 | -0.06144 |
| H | -6.07383 | -2.10576 | -0.38201 | H | -6.0438 | -2.15344 | -0.00396 |
| H | -2.90599 | 1.12599 | 0.86065 | H | -2.9312 | 1.18 | 0.81541 |
| H | -1.88657 | -2.92455 | -1.79051 | H | -1.84687 | -2.89725 | -1.8202 |
| H | -4.20224 | -2.43655 | -1.22967 | H | -4.18105 | -2.39185 | -1.32333 |
| H | 2.61697 | -2.27151 | -1.82139 | H | 2.57199 | -2.30206 | -1.77865 |
| H | 4.705 | -1.1223 | -1.33273 | H | 4.68619 | -1.18866 | -1.26584 |
| H | 4.24083 | 3.0577 | 0.78781 | H | 4.34771 | 3.12637 | 0.36339 |
| H | 4.71444 | 1.87105 | 2.03187 | H | 4.57125 | 2.22288 | 1.8893 |
| H | 5.94157 | 2.73187 | 1.0766 | H | 5.96996 | 2.7291 | 0.9354 |
| H | 7.05505 | 1.19828 | 0.06153 | H | 7.02983 | 1.16519 | 0.0557 |
| H | 6.31821 | -0.3374 | 0.56796 | H | 6.37103 | -0.47117 | 0.11641 |
| H | 6.36774 | 0.13201 | -1.1518 | H | 6.29632 | 0.47664 | -1.39643 |
| H | 2.48149 | 1.92698 | 0.79082 | H | 2.50024 | 1.91088 | 0.8659 |

| Table S28. Coordinates (X, Y and Z) of optimized PPSA structures, expressed in Å. | | | | | | | |
| --- | --- | --- | --- | --- | --- | --- | --- |
|  |  | Anion |  |  |  | Radical |  |
| O | 4.93145 | 0.56426 | -0.88331 | O | 4.90502 | 0.58885 | -0.89896 |
| S | 4.70031 | -0.84719 | -0.42661 | S | 4.69108 | -0.8304 | -0.46162 |
| O | 4.34444 | -1.77985 | -1.54953 | O | 4.31021 | -1.74385 | -1.58983 |
| O | 5.85109 | -1.37081 | 0.38812 | O | 5.84925 | -1.36141 | 0.33218 |
| C | 3.27072 | -0.81109 | 0.69702 | C | 3.28044 | -0.81173 | 0.68986 |
| C | 1.963 | -0.41895 | 0.00915 | C | 1.96364 | -0.44632 | 0.00328 |
| C | 0.8132 | -0.3518 | 1.03425 | C | 0.82049 | -0.36275 | 1.03069 |
| N | -0.50111 | -0.05081 | 0.46675 | N | -0.4756 | -0.05179 | 0.38589 |
| C | -1.27601 | -1.07577 | -0.11151 | C | -1.28306 | -1.0802 | -0.08952 |
| C | -0.83657 | -2.40053 | -0.26168 | C | -0.92753 | -2.44616 | -0.00973 |
| C | -1.67567 | -3.3793 | -0.81083 | C | -1.78066 | -3.41878 | -0.50487 |
| C | -2.96181 | -3.05313 | -1.23756 | C | -3.01085 | -3.06817 | -1.09651 |
| C | -3.41224 | -1.73272 | -1.10553 | C | -3.38113 | -1.73521 | -1.19538 |
| C | -2.5819 | -0.77328 | -0.54499 | C | -2.52152 | -0.75562 | -0.70055 |
| O | -3.10828 | 0.50493 | -0.36569 | O | -2.92765 | 0.53907 | -0.82305 |
| C | -2.18917 | 1.54386 | -0.22805 | C | -2.11789 | 1.54662 | -0.39236 |
| C | -2.64187 | 2.83228 | -0.47198 | C | -2.58709 | 2.84802 | -0.56777 |
| C | -1.78436 | 3.92212 | -0.27127 | C | -1.79646 | 3.90976 | -0.15383 |
| C | -0.47835 | 3.69019 | 0.15672 | C | -0.53666 | 3.6673 | 0.42853 |
| C | -0.02224 | 2.38401 | 0.3813 | C | -0.07236 | 2.37454 | 0.60926 |
| C | -0.87462 | 1.2825 | 0.20592 | C | -0.86377 | 1.27284 | 0.21214 |
| H | 3.54724 | -0.10845 | 1.48999 | H | 3.55715 | -0.09323 | 1.46775 |
| H | 3.21674 | -1.81408 | 1.13226 | H | 3.24817 | -1.80946 | 1.13749 |
| H | 1.7227 | -1.14736 | -0.77082 | H | 1.72306 | -1.19559 | -0.7572 |
| H | 2.07242 | 0.54991 | -0.48751 | H | 2.05734 | 0.51426 | -0.51291 |
| H | 1.02654 | 0.40489 | 1.79544 | H | 1.01024 | 0.40523 | 1.7792 |
| H | 0.73023 | -1.2991 | 1.57649 | H | 0.70255 | -1.29378 | 1.58495 |
| H | 0.16108 | -2.68598 | 0.04923 | H | 0.01266 | -2.74605 | 0.43178 |
| H | -1.30645 | -4.3966 | -0.90481 | H | -1.48886 | -4.46222 | -0.43955 |
| H | -3.61367 | -3.80806 | -1.66674 | H | -3.67287 | -3.84018 | -1.47644 |
| H | -4.41121 | -1.44205 | -1.41744 | H | -4.31788 | -1.43185 | -1.65066 |
| H | -3.66753 | 2.97352 | -0.80002 | H | -3.55985 | 2.99692 | -1.02482 |
| H | -2.14065 | 4.9322 | -0.44897 | H | -2.14914 | 4.92849 | -0.28348 |
| H | 0.20387 | 4.52053 | 0.31479 | H | 0.08493 | 4.5013 | 0.73958 |
| H | 1.00153 | 2.23962 | 0.7051 | H | 0.90539 | 2.22937 | 1.04923 |

| Table S29. Coordinates (X, Y and Z) of optimized PZ structures, expressed in Å. | | | | | | | |
| --- | --- | --- | --- | --- | --- | --- | --- |
|  |  | Cation |  | Radical di-cation | | | |
| C | 4.47312 | -1.02448 | 0.00555 | C | 3.76388 | -1.57675 | -0.92542 |
| N | 3.6257 | -0.00169 | 0.71868 | N | 3.3488 | -0.43195 | -0.02773 |
| C | 3.86652 | 1.37851 | 0.16511 | C | 3.63064 | 0.88423 | -0.71002 |
| C | 2.16321 | -0.41219 | 0.66921 | C | 1.89271 | -0.59777 | 0.3851 |
| C | 1.23992 | 0.45143 | 1.52445 | C | 1.40744 | 0.50194 | 1.33528 |
| C | -0.18695 | -0.11842 | 1.55919 | C | 0.03082 | 0.24712 | 1.98972 |
| N | -0.89324 | -0.09103 | 0.24441 | N | -1.14248 | 0.19109 | 1.06746 |
| C | -1.48427 | 1.18059 | -0.07411 | C | -1.66157 | 1.41715 | 0.62195 |
| C | -0.72458 | 2.15637 | -0.73362 | C | -1.03398 | 2.64165 | 0.96622 |
| C | -1.24938 | 3.42649 | -0.98701 | C | -1.53779 | 3.85783 | 0.53806 |
| C | -2.56259 | 3.72634 | -0.61188 | C | -2.69784 | 3.92521 | -0.25345 |
| C | -3.35169 | 2.74959 | -0.00083 | C | -3.3433 | 2.75339 | -0.60065 |
| C | -2.81418 | 1.4855 | 0.27206 | C | -2.84201 | 1.50819 | -0.17195 |
| S | -3.83348 | 0.25105 | 1.06193 | S | -3.77909 | 0.13062 | -0.65682 |
| C | -3.10886 | -1.21396 | 0.34077 | C | -2.82638 | -1.20788 | -0.09603 |
| C | -3.90609 | -2.34601 | 0.12653 | C | -3.31619 | -2.47796 | -0.45909 |
| C | -3.33582 | -3.51189 | -0.38877 | C | -2.66067 | -3.62615 | -0.05694 |
| C | -1.97689 | -3.53762 | -0.7181 | C | -1.49804 | -3.50877 | 0.72442 |
| C | -1.19219 | -2.39734 | -0.53017 | C | -1.00567 | -2.26702 | 1.08802 |
| C | -1.74141 | -1.22947 | 0.01581 | C | -1.64561 | -1.06464 | 0.69116 |
| H | 5.52012 | -0.73261 | 0.08678 | H | 4.83774 | -1.51066 | -1.10343 |
| H | 4.32147 | -1.99825 | 0.4714 | H | 3.53145 | -2.52264 | -0.43538 |
| H | 4.17672 | -1.05629 | -1.0441 | H | 3.22159 | -1.4933 | -1.86842 |
| H | 3.92252 | 0.02154 | 1.70013 | H | 3.93778 | -0.44964 | 0.81423 |
| H | 3.32816 | 2.1114 | 0.76425 | H | 3.42359 | 1.70715 | -0.02736 |
| H | 3.52537 | 1.4093 | -0.87154 | H | 3.0178 | 0.96432 | -1.60934 |
| H | 4.93383 | 1.59348 | 0.21119 | H | 4.68895 | 0.90921 | -0.97347 |
| H | 1.87772 | -0.39155 | -0.38509 | H | 1.31746 | -0.63019 | -0.54356 |
| H | 2.13717 | -1.4498 | 1.01277 | H | 1.84003 | -1.58179 | 0.85343 |
| H | 1.21282 | 1.48726 | 1.17384 | H | 1.40078 | 1.4649 | 0.82191 |
| H | 1.59777 | 0.47309 | 2.56212 | H | 2.10108 | 0.59345 | 2.17962 |
| H | -0.76198 | 0.43365 | 2.31536 | H | -0.16669 | 1.03839 | 2.71461 |
| H | -0.15433 | -1.16168 | 1.88779 | H | 0.05039 | -0.66337 | 2.58131 |
| H | 0.28229 | 1.90843 | -1.05455 | H | -0.14214 | 2.66331 | 1.57295 |
| H | -0.6385 | 4.17251 | -1.4871 | H | -1.01777 | 4.76598 | 0.82579 |
| H | -2.98217 | 4.7079 | -0.81346 | H | -3.07951 | 4.88476 | -0.5867 |
| H | -4.38437 | 2.96681 | 0.25901 | H | -4.2474 | 2.78095 | -1.20369 |
| H | -4.96577 | -2.3172 | 0.3663 | H | -4.21975 | -2.54461 | -1.05961 |
| H | -3.95656 | -4.38963 | -0.54405 | H | -3.03647 | -4.60447 | -0.33688 |
| H | -0.14051 | -2.40633 | -0.80154 | H | -0.11338 | -2.24741 | 1.69291 |
| H | -1.53015 | -4.4379 | -1.13035 | H | -0.97098 | -4.39698 | 1.05745 |

| Table S30. Coordinates (X, Y and Z) of optimized TH (fully reduced) structures, expressed in Å. | | | | | | | |
| --- | --- | --- | --- | --- | --- | --- | --- |
|  |  | Neutral |  |  |  | Radical |  |
| N | -4.89495 | 0.97268 | 0.82386 | N | -5.08262 | 0.79916 | 0.21568 |
| C | -3.66597 | 0.36644 | 0.50272 | C | -3.8221 | 0.27637 | 0.16098 |
| C | -3.46363 | -1.00875 | 0.70747 | C | -3.59802 | -1.10328 | 0.41795 |
| C | -2.25951 | -1.61393 | 0.34521 | C | -2.32897 | -1.6337 | 0.33099 |
| C | -1.23855 | -0.88128 | -0.27354 | C | -1.22179 | -0.82483 | -0.01189 |
| N | -0.03963 | -1.50147 | -0.70179 | N | 0.03134 | -1.39415 | -0.08916 |
| C | 1.18986 | -0.81078 | -0.5669 | C | 1.23042 | -0.75998 | -0.3301 |
| C | 2.3669 | -1.48104 | -0.21055 | C | 2.42764 | -1.51203 | -0.31607 |
| C | 3.58633 | -0.80712 | -0.13118 | C | 3.64947 | -0.90927 | -0.51753 |
| C | 3.65968 | 0.57331 | -0.37993 | C | 3.73605 | 0.49193 | -0.74101 |
| C | 2.4759 | 1.25482 | -0.70984 | C | 2.54276 | 1.24378 | -0.77732 |
| C | 1.26328 | 0.56835 | -0.81966 | C | 1.30919 | 0.63348 | -0.58337 |
| S | -0.2037 | 1.43716 | -1.36663 | S | -0.13022 | 1.65216 | -0.71043 |
| C | -1.44486 | 0.49067 | -0.48957 | C | -1.44013 | 0.5526 | -0.26264 |
| C | -2.63168 | 1.11118 | -0.08823 | C | -2.72082 | 1.08753 | -0.17994 |
| N | 4.89292 | 1.2527 | -0.36232 | N | 4.94384 | 1.0928 | -0.94289 |
| H | -4.83821 | 1.96967 | 0.99728 | H | -5.21238 | 1.80008 | 0.16574 |
| H | -5.40714 | 0.51386 | 1.56946 | H | -5.83422 | 0.27247 | 0.63998 |
| H | -4.24398 | -1.60801 | 1.17019 | H | -4.43397 | -1.74466 | 0.68047 |
| H | -2.11815 | -2.67616 | 0.5337 | H | -2.17084 | -2.69059 | 0.53174 |
| H | 0.02542 | -2.47624 | -0.42582 | H | 0.07476 | -2.39765 | 0.05528 |
| H | 2.33256 | -2.54771 | 0.00126 | H | 2.37865 | -2.58267 | -0.13674 |
| H | 4.48459 | -1.36003 | 0.13244 | H | 4.55461 | -1.50912 | -0.49919 |
| H | 2.50406 | 2.3254 | -0.89973 | H | 2.59223 | 2.31355 | -0.96494 |
| H | -2.75851 | 2.17944 | -0.24981 | H | -2.87724 | 2.14597 | -0.37357 |
| H | 5.61216 | 0.80313 | 0.19407 | H | 5.80415 | 0.58365 | -0.78661 |
| H | 4.8273 | 2.237 | -0.12285 | H | 5.0091 | 2.10139 | -0.93774 |
| Table S31. Coordinates (X, Y and Z) of optimized TH (fully oxidized) structures, expressed in Å. | | | | | | | |
|  |  | Radical |  |  |  | Cation |  |
| N | -5.10068 | 0.53492 | 0.50515 | N | -5.05521 | 0.52636 | 0.41998 |
| C | -3.81508 | 0.14152 | 0.15622 | C | -3.79435 | 0.13633 | 0.14404 |
| C | -3.60507 | -1.03232 | -0.60658 | C | -3.57464 | -1.03312 | -0.65557 |
| C | -2.32497 | -1.44303 | -0.91969 | C | -2.30423 | -1.43778 | -0.94579 |
| C | -1.16718 | -0.73248 | -0.49632 | C | -1.15681 | -0.71957 | -0.47609 |
| N | 0.04533 | -1.22633 | -0.88209 | N | 0.04468 | -1.1915 | -0.82946 |
| C | 1.21408 | -0.61883 | -0.52563 | C | 1.2035 | -0.62106 | -0.48577 |
| C | 2.42416 | -1.20378 | -0.99306 | C | 2.40618 | -1.2458 | -0.95383 |
| C | 3.66804 | -0.69182 | -0.68154 | C | 3.63967 | -0.73366 | -0.67433 |
| C | 3.78499 | 0.45937 | 0.13412 | C | 3.76416 | 0.46398 | 0.10402 |
| N | 5.02948 | 1.0045 | 0.41556 | N | 4.98462 | 0.96659 | 0.36967 |
| C | 2.60876 | 1.06867 | 0.60349 | C | 2.59661 | 1.10368 | 0.58538 |
| C | 1.3541 | 0.54941 | 0.28116 | C | 1.34777 | 0.57762 | 0.30432 |
| S | -0.05906 | 1.40351 | 0.91982 | S | -0.05926 | 1.4028 | 0.93836 |
| C | -1.40053 | 0.43512 | 0.28905 | C | -1.39581 | 0.45007 | 0.33155 |
| C | -2.69293 | 0.86132 | 0.60006 | C | -2.68316 | 0.86025 | 0.63685 |
| H | -5.85264 | 0.16029 | -0.05968 | H | -5.84447 | 0.08399 | -0.03145 |
| H | -5.23878 | 1.51489 | 0.72652 | H | -5.25453 | 1.36638 | 0.94886 |
| H | -4.45981 | -1.60946 | -0.95027 | H | -4.43008 | -1.59233 | -1.02267 |
| H | -2.16819 | -2.33723 | -1.51601 | H | -2.1214 | -2.32092 | -1.54906 |
| H | 2.34096 | -2.09178 | -1.61276 | H | 2.30058 | -2.14673 | -1.54963 |
| H | 4.56478 | -1.1762 | -1.06024 | H | 4.53681 | -1.22432 | -1.0413 |
| H | 5.10178 | 1.58902 | 1.24037 | H | 5.1063 | 1.82133 | 0.89964 |
| H | 5.83104 | 0.39698 | 0.29465 | H | 5.82652 | 0.51267 | 0.03639 |
| H | 2.68187 | 1.96046 | 1.22276 | H | 2.69556 | 2.00951 | 1.17789 |
| H | -2.83911 | 1.76003 | 1.19584 | H | -2.85293 | 1.74918 | 1.23904 |

| Table S32. Coordinates (X, Y and Z) of optimized TMPD structures, expressed in Å. | | | | | | | |
| --- | --- | --- | --- | --- | --- | --- | --- |
|  |  | Neutral |  |  |  | Radical |  |
| C | 3.3847 | 1.3009 | -0.85948 | C | 3.48186 | 1.36137 | -0.38922 |
| N | 2.78141 | -0.01769 | -0.67186 | N | 2.76305 | 0.08055 | -0.36635 |
| C | 3.63969 | -0.85525 | 0.18198 | C | 3.53492 | -1.15366 | -0.55616 |
| C | 1.40292 | -0.02136 | -0.31012 | C | 1.41958 | 0.04107 | -0.18763 |
| C | 0.7075 | -1.24612 | -0.19306 | C | 0.71382 | -1.20089 | -0.10454 |
| C | -0.64512 | -1.29125 | 0.11759 | C | -0.64659 | -1.24013 | 0.075 |
| C | -1.40021 | -0.11359 | 0.3148 | C | -1.4194 | -0.04082 | 0.18933 |
| C | -0.71455 | 1.10348 | 0.18947 | C | -0.71356 | 1.2011 | 0.10674 |
| C | 0.65784 | 1.1487 | -0.10144 | C | 0.64679 | 1.24036 | -0.07305 |
| N | -2.78033 | -0.20379 | 0.66234 | N | -2.76293 | -0.08026 | 0.36737 |
| C | -3.58797 | -0.99837 | -0.27849 | C | -3.48265 | -1.36061 | 0.38505 |
| C | -3.44385 | 1.06649 | 0.9547 | C | -3.53459 | 1.15363 | 0.55987 |
| H | 2.83406 | 1.8721 | -1.61156 | H | 3.22337 | 1.94332 | -1.28088 |
| H | 4.40691 | 1.17065 | -1.22511 | H | 4.55324 | 1.16803 | -0.40657 |
| H | 3.4329 | 1.89484 | 0.06967 | H | 3.26016 | 1.94943 | 0.50604 |
| H | 4.62695 | -0.9502 | -0.27955 | H | 4.56179 | -0.89664 | -0.81116 |
| H | 3.23159 | -1.86016 | 0.29137 | H | 3.12551 | -1.74874 | -1.37819 |
| H | 3.76728 | -0.42316 | 1.18906 | H | 3.54892 | -1.75938 | 0.35704 |
| H | 1.22949 | -2.18364 | -0.3607 | H | 1.24898 | -2.14036 | -0.16955 |
| H | -1.12008 | -2.26301 | 0.21614 | H | -1.12701 | -2.2079 | 0.1439 |
| H | -1.23173 | 2.04662 | 0.32462 | H | -1.24869 | 2.14057 | 0.17189 |
| H | 1.12602 | 2.124 | -0.16878 | H | 1.12719 | 2.20817 | -0.14179 |
| H | -4.58667 | -1.14898 | 0.14151 | H | -4.55395 | -1.16649 | 0.39754 |
| H | -3.14942 | -1.98313 | -0.44349 | H | -3.22876 | -1.94426 | 1.27694 |
| H | -3.6937 | -0.49892 | -1.25637 | H | -3.2572 | -1.94736 | -0.5101 |
| H | -3.51758 | 1.73223 | 0.07754 | H | -3.5514 | 1.75995 | -0.35289 |
| H | -2.92096 | 1.59725 | 1.75472 | H | -3.1231 | 1.74832 | 1.38108 |
| H | -4.4595 | 0.85973 | 1.3028 | H | -4.56062 | 0.89608 | 0.81768 |

| Table S33. Coordinates (X, Y and Z) of optimized VB (fully reduced) structures, expressed in Å. | | | | | | | |
| --- | --- | --- | --- | --- | --- | --- | --- |
|  |  | Neutral |  |  |  | Radical |  |
| C | 5.70842 | -0.38906 | 0.47595 | C | 5.7259 | -0.1015 | -0.17768 |
| O | 4.50786 | -1.14603 | 0.30083 | O | 4.60033 | -0.97468 | 0.01297 |
| C | 3.30468 | -0.46039 | 0.28322 | C | 3.36799 | -0.42088 | 0.20101 |
| C | 3.17963 | 0.92591 | 0.42786 | C | 3.11458 | 0.96001 | 0.20926 |
| C | 1.91328 | 1.51918 | 0.3892 | C | 1.81456 | 1.41946 | 0.40535 |
| C | 0.74842 | 0.75418 | 0.21375 | C | 0.75417 | 0.5185 | 0.59209 |
| N | -0.49981 | 1.41428 | 0.15671 | N | -0.53328 | 1.04438 | 0.81298 |
| C | -1.75035 | 0.7355 | 0.13423 | C | -1.75401 | 0.53947 | 0.46112 |
| C | -2.12198 | -0.06008 | -0.96078 | C | -1.92515 | -0.52873 | -0.46441 |
| C | -3.35641 | -0.70718 | -0.99147 | C | -3.18636 | -0.97442 | -0.78255 |
| C | -4.27521 | -0.56066 | 0.06392 | C | -4.34385 | -0.37664 | -0.20351 |
| N | -5.54513 | -1.15924 | 0.00123 | N | -5.57679 | -0.82807 | -0.51386 |
| C | -3.91602 | 0.26022 | 1.14605 | C | -4.17241 | 0.71127 | 0.7022 |
| C | -2.66678 | 0.88299 | 1.18593 | C | -2.91351 | 1.15459 | 1.0192 |
| C | 0.89036 | -0.63959 | 0.08114 | C | 1.02003 | -0.86524 | 0.61408 |
| C | 2.1516 | -1.23411 | 0.10865 | C | 2.31231 | -1.32599 | 0.41413 |
| H | 5.85617 | 0.32146 | -0.34608 | H | 5.59582 | 0.51743 | -1.07159 |
| H | 5.70755 | 0.14496 | 1.43359 | H | 5.88431 | 0.52775 | 0.70417 |
| H | 6.52274 | -1.11548 | 0.47241 | H | 6.58574 | -0.75778 | -0.31452 |
| H | 4.04775 | 1.56028 | 0.56596 | H | 3.91011 | 1.6794 | 0.05943 |
| H | 1.83754 | 2.59928 | 0.49396 | H | 1.62311 | 2.48914 | 0.39732 |
| H | -0.52101 | 2.27646 | 0.68841 | H | -0.56062 | 1.93599 | 1.29951 |
| H | -1.44232 | -0.17031 | -1.80096 | H | -1.06664 | -0.97292 | -0.95343 |
| H | -3.61454 | -1.32204 | -1.85077 | H | -3.31139 | -1.77946 | -1.50081 |
| H | -5.59128 | -1.98734 | -0.58346 | H | -5.71167 | -1.5813 | -1.17834 |
| H | -5.97866 | -1.33141 | 0.90247 | H | -6.4148 | -0.38244 | -0.15663 |
| H | -4.60981 | 0.39988 | 1.97198 | H | -5.04405 | 1.18859 | 1.14051 |
| H | -2.40495 | 1.49286 | 2.04773 | H | -2.79236 | 1.98052 | 1.7147 |
| H | 0.01752 | -1.27074 | -0.04111 | H | 0.22996 | -1.57546 | 0.83012 |
| H | 2.24489 | -2.31127 | 0.00404 | H | 2.52625 | -2.38964 | 0.44331 |

| Table S34. Coordinates (X, Y and Z) of optimized VB (fully oxidized) structures, expressed in Å. | | | | | | | |
| --- | --- | --- | --- | --- | --- | --- | --- |
|  |  | Radical |  |  |  | Cation |  |
| C | 5.56286 | 0.21111 | -1.13971 | C | 5.57057 | 0.17832 | -1.07132 |
| O | 4.27743 | 0.37702 | -1.75349 | O | 4.28071 | 0.38308 | -1.68136 |
| C | 3.16191 | -0.00964 | -1.04877 | C | 3.16522 | -0.00061 | -1.02444 |
| C | 3.18841 | -0.55225 | 0.24431 | C | 3.165 | -0.6408 | 0.23155 |
| C | 1.99284 | -0.91101 | 0.86463 | C | 1.95848 | -1.01262 | 0.80026 |
| C | 0.73565 | -0.71637 | 0.24653 | C | 0.71801 | -0.6949 | 0.18666 |
| N | -0.38556 | -1.15237 | 0.93008 | N | -0.40429 | -1.08963 | 0.86167 |
| C | -1.60758 | -0.57447 | 0.78632 | C | -1.60027 | -0.53187 | 0.76219 |
| C | -1.87375 | 0.73347 | 0.27213 | C | -1.90491 | 0.7644 | 0.18567 |
| C | -3.15786 | 1.24295 | 0.2242 | C | -3.18292 | 1.23262 | 0.1487 |
| C | -4.26495 | 0.48538 | 0.67783 | C | -4.26333 | 0.44627 | 0.68242 |
| N | -5.54224 | 0.9864 | 0.5763 | N | -5.50999 | 0.90887 | 0.64062 |
| C | -4.0204 | -0.80278 | 1.21465 | C | -3.97871 | -0.81727 | 1.30261 |
| C | -2.73457 | -1.30106 | 1.28276 | C | -2.69493 | -1.26585 | 1.36292 |
| C | 0.74273 | -0.18682 | -1.07269 | C | 0.745 | -0.08473 | -1.10268 |
| C | 1.93101 | 0.14971 | -1.70722 | C | 1.94549 | 0.24235 | -1.69732 |
| H | 6.28718 | 0.58174 | -1.86602 | H | 6.2923 | 0.58533 | -1.77891 |
| H | 5.76838 | -0.84417 | -0.9284 | H | 5.76794 | -0.88697 | -0.91928 |
| H | 5.64012 | 0.80109 | -0.21969 | H | 5.63925 | 0.72264 | -0.12449 |
| H | 4.12291 | -0.70211 | 0.77253 | H | 4.0908 | -0.86283 | 0.74809 |
| H | 2.01733 | -1.34185 | 1.86118 | H | 1.94342 | -1.52265 | 1.75813 |
| H | -1.05134 | 1.36502 | -0.0442 | H | -1.09342 | 1.40071 | -0.14593 |
| H | -3.32079 | 2.24988 | -0.15341 | H | -3.39906 | 2.22071 | -0.24795 |
| H | -6.29197 | 0.54214 | 1.09127 | H | -6.28154 | 0.39375 | 1.05137 |
| H | -5.65828 | 1.97788 | 0.42337 | H | -5.74362 | 1.78386 | 0.18423 |
| H | -4.85274 | -1.39642 | 1.58508 | H | -4.7956 | -1.395 | 1.72501 |
| H | -2.55664 | -2.28306 | 1.71162 | H | -2.46045 | -2.21591 | 1.83229 |
| H | -0.18819 | -0.087 | -1.62079 | H | -0.17448 | 0.05078 | -1.66142 |
| H | 1.92303 | 0.53566 | -2.7225 | H | 1.97537 | 0.67297 | -2.69312 |

| Table S35. Parameters of reorganization energies of substrates used, calculated according to Eq. S7. | | | | | | | | | | |
| --- | --- | --- | --- | --- | --- | --- | --- | --- | --- | --- |
| Compound and its oxidation state | | Energy, Hartree | Zero-point energy, kcal/mol | E(A at A^+^), Hartree | E(A at A), Hartree | E(A^+^ at A), Hartree | E(A^+^ at A), Hartree | λ_1_, eV | λ_2_, eV | λ, kcal/mol |
| ABTS | Neutral | -2957.9958 | 228.700 | -2957.9871 | -2957.9958 | -2957.8255 | -2957.8234 | 0.238 | 0.059 | 3.420 |
|  | Radical cation | -2957.8234 | 229.791 |  |  |  |  |  |  |  |
| AMB | Neutral | -652.4551 | 195.042 | -652.4424 | -652.4551 | -652.2706 | -652.2935 | 0.346 | 0.623 | 11.168 |
|  | Radical cation | -652.2935 | 196.875 |  |  |  |  |  |  |  |
| CPZ | Cation | -1627.0114 | 233.621 | -1627.0029 | -1627.0114 | -1626.7985 | -1626.8283 | 0.232 | 0.812 | 12.039 |
|  | Radical di-cation | -1626.8283 | 234.398 |  |  |  |  |  |  |  |
| DCPIP(I), 2^nd^ electron reduction | Anion | -1587.3426 | 102.397 | -1587.3276 | -1587.3426 | -1587.1694 | -1587.1942 | 0.408 | 0.674 | 12.478 |
|  | Radical | -1587.1942 | 117.852 |  |  |  |  |  |  |  |
| DCPIP(I), 1^st^ electron reduction | Radical anion | -1586.7237 | 108.592 | -1586.7154 | -1586.7237 | -1586.5744 | -1586.5823 | 0.225 | 0.216 | 5.084 |
|  | Anion | -1586.5823 | 110.325 |  |  |  |  |  |  |  |
| DCPIP(II) | Anion | -1586.1328 | 102.310 | -1586.1265 | -1586.1328 | -1585.9450 | -1585.9506 | 0.153 | 0.173 | 3.765 |
|  | Radical | -1585.9506 | 102.086 |  |  |  |  |  |  |  |
| DMB | Neutral | -805.0177 | 224.359 | -805.0015 | -805.0177 | -804.8323 | -804.8513 | 0.439 | 0.519 | 11.036 |
|  | Radical cation | -804.8513 | 225.726 |  |  |  |  |  |  |  |
| HEPX | Neutral | -746.1122 | 163.440 | -746.1080 | -746.1122 | -745.9322 | -745.9379 | 0.113 | 0.156 | 3.104 |
|  | Radical cation | -745.9379 | 164.585 |  |  |  |  |  |  |  |
| MB (fully reduced) | Neutral | -1183.0739 | 218.201 | -1183.0624 | -1183.0739 | -1182.9042 | -1182.9218 | 0.313 | 0.478 | 9.123 |
|  | Radical | -1182.9218 | 219.262 |  |  |  |  |  |  |  |
| MB (fully oxidized) | Radical | -1182.4580 | 209.096 | -1182.4518 | -1182.4580 | -1182.3119 | -1182.3204 | 0.170 | 0.231 | 4.629 |
|  | Cation | -1182.3204 | 209.608 |  |  |  |  |  |  |  |
| PPSA | Anion | -1333.4541 | 182.412 | -1333.4483 | -1333.4541 | -1333.2787 | -1333.2814 | 0.156 | 0.073 | 2.647 |
|  | Radical | -1333.2814 | 184.107 |  |  |  |  |  |  |  |
| PZ | Cation | -1167.4805 | 240.251 | -1167.4716 | -1167.4805 | -1167.2715 | -1167.3004 | 0.241 | 0.784 | 11.820 |
|  | Radical di-cation | -1167.3004 | 241.303 |  |  |  |  |  |  |  |
| TH (fully reduced) | Neutral | -1025.9687 | 142.795 | -1025.9569 | -1025.9687 | -1025.7968 | -1025.8132 | 0.321 | 0.446 | 8.851 |
|  | Radical | -1025.8132 | 144.258 |  |  |  |  |  |  |  |
| TH (fully oxidized) | Radical | -1025.3520 | 134.041 | -1025.3452 | -1025.3520 | -1025.2027 | -1025.2115 | 0.187 | 0.240 | 4.916 |
|  | Cation | -1025.2115 | 133.696 |  |  |  |  |  |  |  |
| TMPD | Neutral | -499.8920 | 165.781 | -499.8805 | -499.8920 | -499.7104 | -499.7361 | 0.311 | 0.702 | 11.678 |
|  | Radical | -499.7361 | 167.622 |  |  |  |  |  |  |  |
| VB (fully reduced) | Neutral | -688.1665 | 166.811 | -688.1574 | -688.1665 | -687.9881 | -688.0050 | 0.245 | 0.461 | 8.149 |
|  | Radical | -688.0050 | 168.149 |  |  |  |  |  |  |  |
| VB (fully oxidized) | Radical | -687.5394 | 157.548 | -687.5334 | -687.5394 | -687.3774 | -687.3853 | 0.163 | 0.215 | 4.353 |
|  | Cation | -687.3853 | 160.224 |  |  |  |  |  |  |  |

# 9. Docking studies and calculations of solvent accessible surface areas

Docking studies were performed using an AutoDock Vina software. Structure of *Coprinopsis cinerea* peroxidase PDBID:1h3j was cleaned from co-crystalized molecules and waters. Iron-oxygen distance was reduced to reflect CpdII state. All molecules were docked near the active center (heme) using 30x30x30 Å docking box. All 20 conformations were docked in various orientations near heme and all conformations exhibited very similar ratio (±10%) of the solvent-accessible surface (SAS) of a substrate in docked enzyme-substrate complexes with SAS of free substrate. Therefore, the ratio was estimated using the best docked structures by the AutoDock Vina score for each of the substrate.

| Table S36. The best docked structures of compounds used in the active center of CIP. | |
| --- | --- |
| 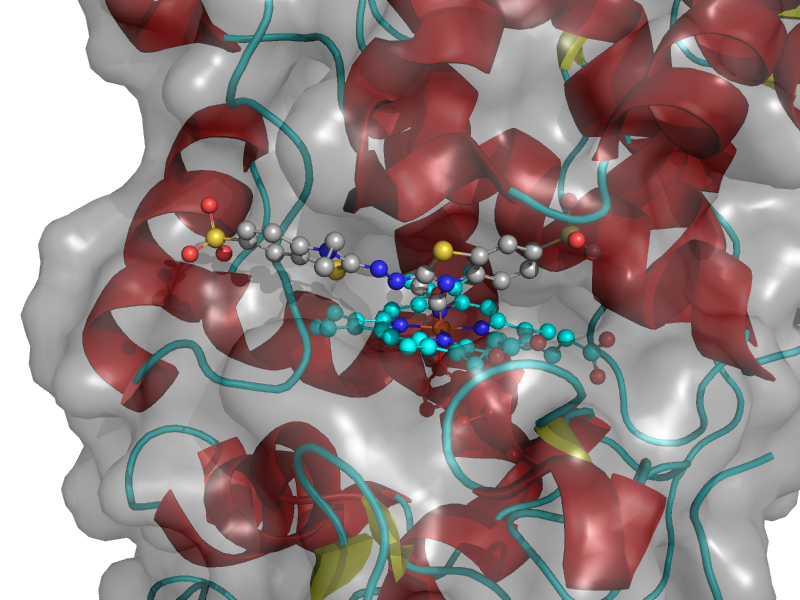  ABTS | 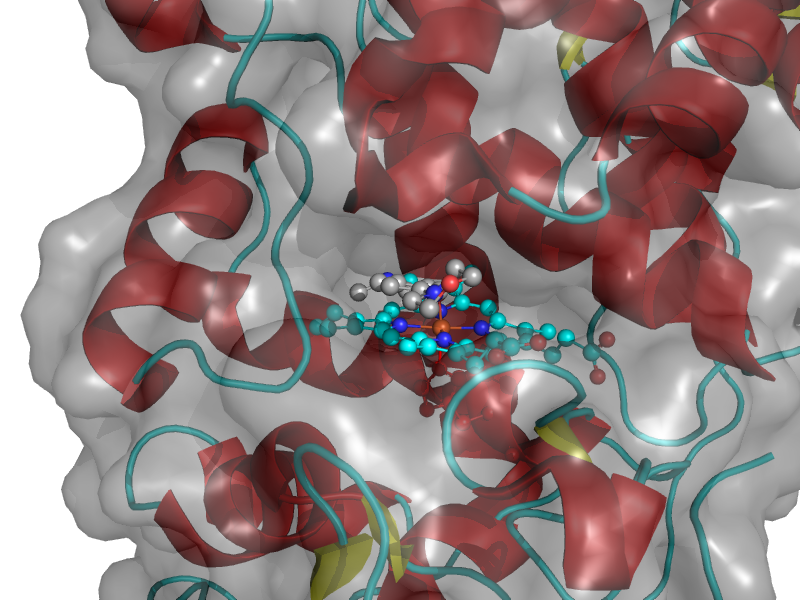  AMB |
| 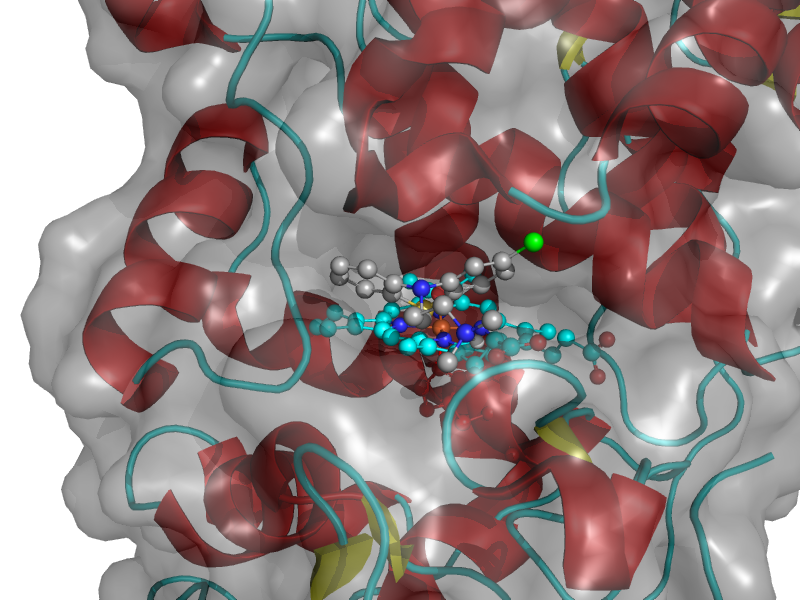  CPZ | 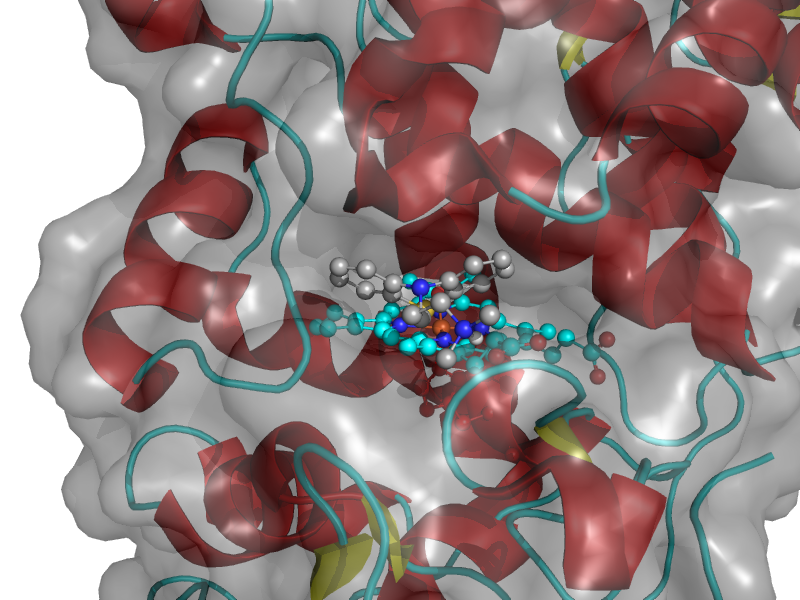  PZ |
| 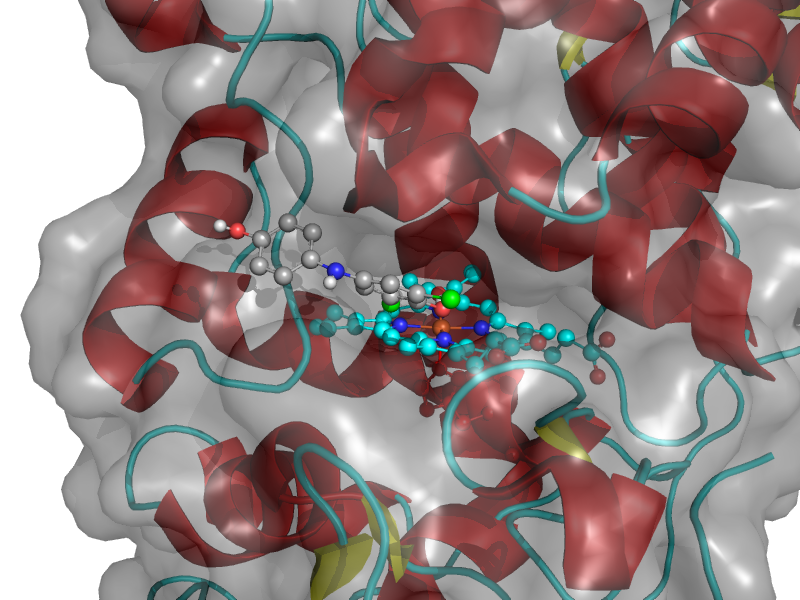  DCPIP(I) | 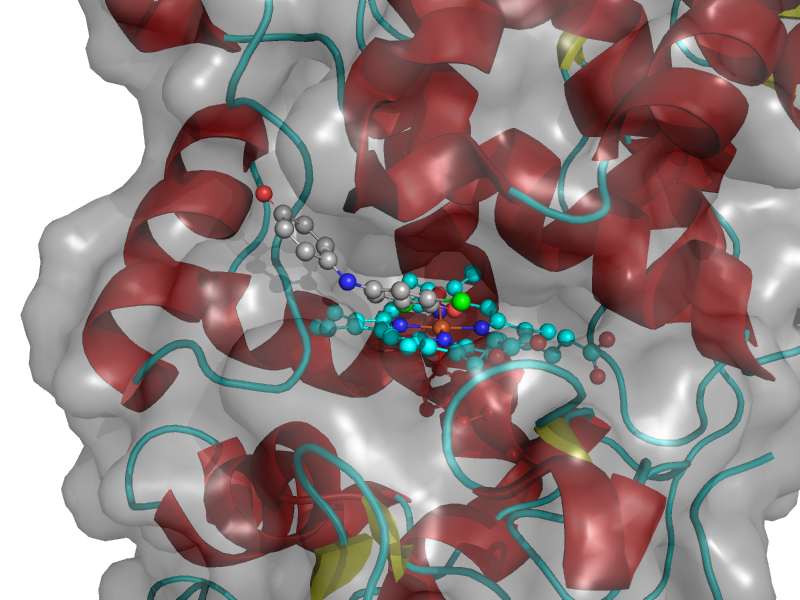  DCPIP(II) |
| 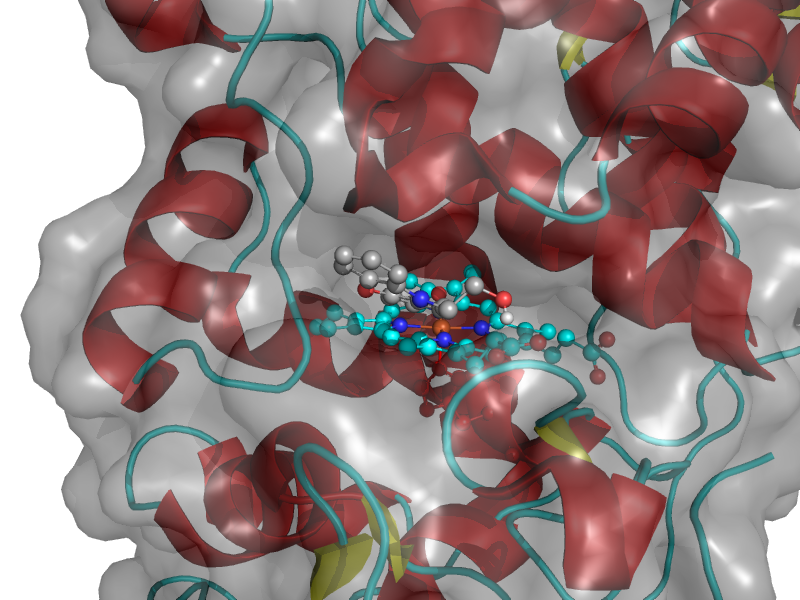  HEPX | PPSA |
| MB (fully reduced) | TH (fully reduced) |
| DMB | TMPD |
| VB |  |

Table S37. Docking scores of structures bound to CIP (Table S36) and relative solvent-accessible surface areas (*θ*).

| **Substrate** | **Docking score/affinity,**  **kcal/mol** | | ***θ*** | |
| --- | --- | --- | --- | --- |
| ABTS |  | -7.6 |  | 0.43 |
| AMB |  | -5.2 |  | 0.36 |
| CPZ |  | -5.8 |  | 0.41 |
| DCPIP(I) |  | -6.2 |  | 0.36 |
| DCPIP(II) |  | -6.7 |  | 0.39 |
| DMB |  | -5.6 |  | 0.37 |
| HEPX |  | -6.6 |  | 0.35 |
| MB |  | -6.4 |  | 0.41 |
| PPSA |  | -6.9 |  | 0.37 |
| PZ | | -5.8 | | 0.39 |
| TH | | -6.1 | | 0.36 |
| TMPD | | -4.8 | | 0.36 |
| VB | | -5.6 | | 0.38 |

**10. Derivation of the apparent bimolecular rate constant**

The ET followed by PT (and *vice versa*) can be described by two reaction schemes presented below:

$\begin{aligned} \mathrm{CpdII}+S\underset{\to}{k_{\mathrm{ET}}}\mathrm{CpdIIS} \\ \mathrm{CpdIIS}\underset{\to}{k_{\mathrm{PT}}}E_{\mathrm{red}}+S^{\cdot+} \end{aligned}$ $\begin{aligned} \mathrm{CpdII}\underset{\to}{k_{\mathrm{PT}}}\mathrm{CpdI}I^{'} \\ CpdII'+S\underset{\to}{k_{\mathrm{PT}}}E_{\mathrm{red}}+S^{\cdot+} \end{aligned}$ (S43)

Assuming that CpdI reduction to CpdII and *E*_red_ oxidation to CpdI do not limit the rate of the reaction, it can be described as:

$\begin{aligned} E+S\underset{\to}{k_{\mathrm{ET}}}\mathrm{ES} \\ \mathrm{ES}\underset{\to}{k_{\mathrm{PT}}}E+S^{\cdot+} \end{aligned}$ $\begin{aligned} E\underset{\to}{k_{\mathrm{PT}}}E` \\ E`+S\underset{\to}{k_{\mathrm{ET}}}E+S^{\cdot+} \end{aligned}$ (S44)

These schemes can be converted to the systems of differential equations:

$\left\{ \begin{aligned} [E]'=-k_{\mathrm{ET}}[E][S]+k_{\mathrm{PT}}[ES] \\ [ES]'=k_{\mathrm{ET}}[E][S]-k_{\mathrm{PT}}[ES] \end{aligned} \right. \left\{ \begin{aligned} [E]'=k_{\mathrm{ET}}[E'][S]-k_{\mathrm{PT}}[E] \\ [E']'=-k_{\mathrm{ET}}[E'][S]+k_{\mathrm{PT}}[E] \end{aligned} \right.$ (S45)

with additional mass balance equations for an enzyme:

${[E}_{0}]=[ES]+[E] {[E}_{0}]=[E']+[E]$ (S46)

Assuming quasi-steady state conditions, both equations will result in the same substrate oxidation rate equation:

$v=\frac{d[S^{\cdot+}]}{dt}=\frac{[E_{0}]{[S]k}_{\mathrm{ET}}k_{\mathrm{PT}}}{k_{\mathrm{PT}}+[S]k_{ET}}$ (S47)

Therefore, apparent bimolecular rate constants could be calculated using the following equation:

$k_{2}=\frac{k_{\mathrm{ET}}k_{\mathrm{PT}}}{k_{\mathrm{PT}}+[S]k_{\mathrm{ET}}}$ (S48)

# 11. Ыubstrates’ pKa and their relevance to the oxidation *via* radical pathway

As mentioned in the manuscript, 9 substrates are oxidized via one-electron oxidation pathway to the radical cations and do not donate proton for the overall reaction. The data are summarized in Table S38. The most important factor to conclude whether a proton is involved in the oxidation of substrates is pKa values. The measurements of kinetics were carried out in at pH 7, therefore, pKa values should be substantially different from pH to dismiss proton involvement in the reaction. It can be seen from Table S38 than only TMPD’s pH value is relatively close to 7. Thus, one could conclude that at least 18% of TMPD is protonated and could influence the reaction rate, but only if it is preferred substrate form of peroxidases. However, in such case the TMPD oxidation reaction rate should drop with increasing pH. In the reaction rate profile (Figure S30) an entirely different reaction rate profile is evident, which supports our conclusion that at pH 7 the TMPD oxidation proceeds via the radical pathway, without donation of proton to the reaction.

Table S38. pKa values of substrates used in oxidation experiments.

| Compound | pKa | Notes | References |
| --- | --- | --- | --- |
| ABTS | ~3 | Sulfonic group | [27,33] |
| AMB | 5.7 |  |  |
| CPZ | 9.15 | Amino group in sidechain | [34] |
| DCPIP | 5.6 | pKa of oxidized form, or which further oxidation follows radical pathway | [35] |
| DMB | 4.2 |  | [27] |
| HEPX | >13 | Hydroxyethyl group, pH independent redox potential |  |
| PPSA | <2 | Sulfonic group, pH-independent redox potential |  |
| PZ | 9.37 | Amino group in sidechain | [34] |
| TMPD | 6.35 |  | [36] |

Figure S30. TMPD oxidation reaction rate dependence on pH, performed in 50mM potassium phosphate buffer solution, in presence of 16 nM rCIP, 150 µM H_2_O_2_, and 9.5 µM TMPD. Reaction progress curves were fitted to the pseudo first order reaction and apparent first order reaction rate constant reported here.

**12. Standard reduction potentials of CIP and HRP**

Standard reduction potentials of CIP and HRP are published in the literature. However, there is a substantial obscurity about the process those potentials represent:

$E_{ox}+e^{-}\leftrightarrow E_{red}$ (S49)

$E_{ox}+e^{-}+H^{+}\leftrightarrow E_{red}$ (S50)

In this work, the published reduction potentials at pH 7 were used, as if they were given for the process illustrated in Equation S49. Despite different opinions in the literature there are many evidences that these reduction potentials represent the reaction presented in Equation S49. On the one hand, Farhangrazi et al. (Biochemistry, 1994, 33, 5647-5652) concluded that additional studies are required to understand how the proton takes part in the reaction. On the other hand, Hayashi and Yamazaki (J Biol Chem, 1979, 254, 9101-9106) suggested that compound II reduction potentials in a pH range of 7-8 are pH-independent and, therefore, in this pH range PT is not involved in the limiting step of the oxidation reaction. Also, it was shown that experimentally determined reduction potential of HRP allowed correct calculation of rate constants of ferrocyanide oxidation using Marcus theory of ET (Biochemistry, 1995, 34, 2866-2871). Moreover, M. Ayala et al. (Biochem Biophys Res Commun, 2007, 357, 804–808) showed that by using experimentally determined rate constants for a series of compounds and Marcus theory of ET it was possible to calculate standard reduction potentials of the enzymes, which were in agreement with values measured using other methods. Therefore, we believe that the literature supports the use of reported standard reduction potentials assuming “Oxidized + e- to Reduced-” process.

# Supporting references

[1] W.L.F. Armarego, C.L.L. Chai, Common Physical Techniques Used in Purification, in: W.L.F. Armarego, C.B.T.-P. of L.C. (Seventh E. Chai (Eds.), Purif. Lab. Chem., Butterworth-Heinemann, Boston, 2009: pp. 1–60. doi:10.1016/b978-1-85617-567-8.50009-3.

[2] A.G. Hildebrandt, I. Roots, Reduced nicotinamide adenine dinucleotide phosphate (NADPH)-dependent formation and breakdown of hydrogen peroxide during mixed function oxidation reactions in liver microsomes, Arch. Biochem. Biophys. 171 (1975) 385–397. doi:10.1016/0003-9861(75)90047-8.

[3] M.B. Andersen, Y. Hsuanyu, K.G. Welinder, P. Schneider, H.B. Dunford, N.Å. Frøystein, G.W. Francis, B. Karlsson, Spectral and Kinetic Properties of Oxidized Intermediates of Coprinus cinereus Peroxidase., Acta Chem. Scand. 45 (2008) 1080–1086. doi:10.3891/acta.chem.scand.45-1080.

[4] R.E. Childs, W.G. Bardsley, The steady-state kinetics of peroxidase with 2,2’-azino-di-(3-ethyl-benzthiazoline-6-sulphonic acid) as chromogen., Biochem. J. 145 (1975) 93–103. doi:10.1042/bj1450093.

[5] J. Kulys, T. Buch-Rasmussen, K. Bechgaard, V. Razumas, J. Kazlauskaite, J. Marcinkeviciene, J.B. Christensen, H.E. Hansen, Study of the new electron transfer mediators in glucose oxidase catalysis, J. Mol. Catal. 91 (1994) 407–420. doi:10.1016/0304-5102(94)00042-5.

[6] J. Kulys, K. Krikstopaitis, A. Ziemys, Kinetics and thermodynamics of peroxidase- and laccase-catalyzed oxidation of N-substituted phenothiazines and phenoxazines, J. Biol. Inorg. Chem. 5 (2000) 333–340. doi:10.1007/PL00010662.

[7] J. McD. Armstrong, The molar extinction coefficient of 2,6-dichlorophenol indophenol, BBA - Gen. Subj. 86 (1964) 194–197. doi:10.1016/0304-4165(64)90180-1.

[8] U. Nickel, M. Borchardt, M.R. Bapat, W. Jaenicke, 1- and 2-Electron Steps in the Oxidation of Substituted p-Phenylenediamines with Different Oxidants in Aqueous Solution I. The Reaction with Iodine, Berichte Der Bunsengesellschaft Für Phys. Chemie. 83 (1979) 877–884. doi:10.1002/bbpc.19790830902.

[9] L.M. Fischer, M. Tenje, A.R. Heiskanen, N. Masuda, J. Castillo, A. Bentien, J. Émneus, M.H. Jakobsen, A. Boisen, Gold cleaning methods for electrochemical detection applications, Microelectron. Eng. 86 (2009) 1282–1285. doi:10.1016/j.mee.2008.11.045.

[10] A. Bard, L. Faulkner, Allen J. Bard and Larry R. Faulkner, Electrochemical Methods: Fundamentals and Applications, New York: Wiley, 2001, 2002. doi:10.1023/A:1021637209564.

[11] A.K. Covington, M. Paabo, R.A. Robinson, R.G. Bates, Use of the Glass Electrode in Deuterium Oxide and the Relation between the Standardized pD (paD) Scale and the Operational pH in Heavy Water, Anal. Chem. 40 (1968) 700–706. doi:10.1021/ac60260a013.

[12] M.W. Schmidt, K.K. Baldridge, J.A. Boatz, S.T. Elbert, M.S. Gordon, J.H. Jensen, S. Koseki, N. Matsunaga, K.A. Nguyen, S. Su, T.L. Windus, M. Dupuis, J.A. Montgomery, General atomic and molecular electronic structure system, J. Comput. Chem. 14 (1993) 1347–1363. doi:10.1002/jcc.540141112.

[13] M.S. Gordon, M.W. Schmidt, Advances in electronic structure theory: GAMESS a decade later, in: C.E. Dykstra, G. Frenking, K.S. Kim, G.E. Scuseria (Eds.), Theory Appl. Comput. Chem., Elsevier, Amsterdam, 2005: pp. 1167–1189. doi:10.1016/B978-044451719-7/50084-6.

[14] W. Kohn, L.J. Sham, Self-consistent equations including exchange and correlation effects, Phys. Rev. 140 (1965). doi:10.1103/PhysRev.140.A1133.

[15] A.D. Becke, A new mixing of Hartree-Fock and local density-functional theories, J. Chem. Phys. 98 (1993) 1372–1377. doi:10.1063/1.464304.

[16] A. V. Marenich, C.J. Cramer, D.G. Truhlar, Universal solvation model based on solute electron density and on a continuum model of the solvent defined by the bulk dielectric constant and atomic surface tensions, J. Phys. Chem. B. 113 (2009) 6378–6396. doi:10.1021/jp810292n.

[17] J.S. Bader, C.M. Cortis, B.J. Berne, Solvation and reorganization energies in polarizable molecular and continuum solvents, J. Chem. Phys. 106 (1997) 2372–2387. doi:10.1063/1.473790.

[18] H. Reiss, A. Heller, The absolute potential of the standard hydrogen electrode: A new estimate, J. Phys. Chem. 89 (1985) 4207–4213. doi:10.1021/j100266a013.

[19] M. v. Smoluchowski, Grundriß der Koagulationskinetik kolloider Lösungen, Kolloid-Zeitschrift. 21 (1917) 98–104. doi:10.1007/BF01427232.

[20] A. Einstein, Über die von der molekularkinetischen Theorie der Wärme geforderte Bewegung von in ruhenden Flüssigkeiten suspendierten Teilchen [AdP 17, 549 (1905)], Ann. Phys. 14 (2005) 182–193. doi:10.1002/andp.200590005.

[21] W. Graebel, A. Paintal, Engineering Fluid Mechanics, 2001. doi:10.1115/1.1399677.

[22] K. Houborg, P. Harris, J. Petersen, P. Rowland, J.C.N. Poulsen, P. Schneider, J. Vind, S. Larsen, Impact of the physical and chemical environment on the molecular structure of Coprinus cinereus peroxidase, Acta Crystallogr. - Sect. D Biol. Crystallogr. 59 (2003) 989–996. doi:10.1107/S0907444903006772.

[23] G.I. Berglund, G.H. Carlsson, A.T. Smith, H. Szöke, A. Henriksen, J. Hajdu, The catalytic pathway of horseradish peroxidase at high resolution, Nature. 417 (2002) 463–468. doi:10.1038/417463a.

[24] L.P. Candeias, L.K. Folkes, M. Porssa, J. Parrick, P. Wardman, Rates of reaction of indoleacetic acids with horseradish peroxidase compound I and their dependence on the redox potentials, Biochemistry. 35 (1996) 102–108. doi:10.1021/bi9514424.

[25] L.P. Candeias, L.K. Folkes, P. Wardman, Factors controlling the substrate specificity of peroxidases: Kinetics and thermodynamics of the reaction of horseradish peroxidase compound I with phenols and indole-3-acetic acids, Biochemistry. 36 (1997) 7081–7085. doi:10.1021/bi970384e.

[26] L.K. Folkes, L.P. Candeias, Interpretation of the reactivity of peroxidase compounds I and II with phenols by the Marcus equation, FEBS Lett. 412 (1997) 305–308. doi:10.1016/S0014-5793(97)00792-8.

[27] K. Krikstopaitis, J. Kulys, A.H. Pedersen, P. Schneider, L. Barré, O. Hammerich, I. Søtofte, B. Långström, N-Substituted p-Phenylenediamines as Peroxidase and Laccase Substrates., Acta Chem. Scand. 52 (1998) 469–474. doi:10.3891/acta.chem.scand.52-0469.

[28] D.W. Hay, S.A. Martin, S. Ray, N.N. Lichtin, Disproportionation of semimethylene blue and oxidation of leucomethylene blue by methylene blue and by iron(III). Kinetics, equilibriums, and medium effects, J. Phys. Chem. 85 (2005) 1474–1479. doi:10.1021/j150611a005.

[29] L.E. Roy, E. Jakubikova, M. Graham Guthrie, E.R. Batista, Calculation of one-electron redox potentials revisited. Is it possible to calculate accurate potentials with density functional methods?, J. Phys. Chem. A. 113 (2009) 6745–6750. doi:10.1021/jp811388w.

[30] D. Borgis, J.T. Hynes, Curve crossing formulation for proton transfer reactions in solution, J. Phys. Chem. 100 (1996) 1118–1128. doi:10.1021/jp9522324.

[31] L. Piela, Exact Solutions–Our Beacons, in: L. Piela (Ed.), Ideas Quantum Chem., Second Edi, Elsevier, Oxford, 2013: pp. 159–229. doi:10.1016/b978-0-444-59436-5.00004-0.

[32] A. Farazdel, M. Dupuis, E. Clementi, A. Aviram, Electric Field Induced Intramolecular Electron Transfer in Spiro π-Electron Systems and Their Suitability as Molecular Electronic Devices. A Theoretical Study, J. Am. Chem. Soc. 112 (1990) 4206–4214. doi:10.1021/ja00167a016.

[33] S.L. Scott, W.J. Chen, A. Bakac, J.H. Espenson, Spectroscopic parameters, electrode potentials, acid ionization constants, and electron exchange rates of the 2,2′-azinobis(3-ethylbenzothiazoline-6-sulfonate) radicals and ions, J. Phys. Chem. 97 (1993) 6710–6714. doi:10.1021/j100127a022.

[34] U. Domańska, A. Pelczarska, A. Pobudkowska, Solubility and pK a determination of six structurally related phenothiazines, Int. J. Pharm. 421 (2011) 135–144. doi:10.1016/j.ijpharm.2011.09.040.

[35] H.A. Hamad, W.A. Sadik, M.M. Abd El-latif, A.B. Kashyout, M.Y. Feteha, Photocatalytic parameters and kinetic study for degradation of dichlorophenol-indophenol (DCPIP) dye using highly active mesoporous TiO2 nanoparticles, J. Environ. Sci. (China). 43 (2016) 26–39. doi:10.1016/j.jes.2015.05.033.

[36] A.N. Pankratov, I.M. Uchaeva, S.Y. Doronin, R.K. Chernova, Gaseous-phase proton affinity of anilines: A quantum chemical evaluation and discussion in view of aqueous basicity, J. Serbian Chem. Soc. 66 (2001) 161–172.

[37] Z.S. Farhangrazi, I. Yamazaki, L.S. Powers, B.R. Copeland, T. Nakayama, T. Amachi, Oxidation-Reduction Properties of Compounds I and II of Arthromyces ramosus Peroxidase, Biochemistry. 33 (1994) 5647–5652. doi:10.1021/bi00184a038.

[38] Y. Hayashi, I. Yamazaki, The oxidation-reduction potentials of compound I/compound II and compound II/ferric couples of horseradish peroxidases A2 and C., J. Biol. Chem. 254 (1979) 9101–9106.

[39] Z.S. Farhangrazi, M.E. Fossett, L.S. Powers, W.R. Ellis, Variable-Temperature Spectroelectrochemical Study of Horseradish Peroxidase, Biochemistry. 34 (1995) 2866–2871. doi:10.1021/bi00009a017.

[40] M. Ayala, R. Roman, R. Vazquez-Duhalt, A catalytic approach to estimate the redox potential of heme-peroxidases, Biochem. Biophys. Res. Commun. 357 (2007) 804–808. doi:10.1016/j.bbrc.2007.04.020.
